# A comprehensive framework for trans-ancestry pathway analysis using GWAS summary data from diverse populations

**DOI:** 10.1371/journal.pgen.1011322

**Published:** 2024-10-23

**Authors:** Sheng Fu, William Wheeler, Xiaoyu Wang, Xing Hua, Devika Godbole, Jubao Duan, Bin Zhu, Lu Deng, Fei Qin, Haoyu Zhang, Jianxin Shi, Kai Yu

**Affiliations:** 1 School of Statistics and Data Science, Nankai University, Tianjin, China; 2 Key Laboratory of Pure Mathematics and Combinatorics, Nankai University, Tianjin, China; 3 Information Management Services, Inc, Bethesda, Maryland, United States of America; 4 Division of Cancer Epidemiology and Genetics, National Cancer Institute, Bethesda, Maryland, United States of America; 5 Cancer Genomics Research Laboratory, Frederick National Laboratory for Cancer Research, Leidos Biomedical Research Inc, Rockville, Maryland, United States of America; 6 Center for Psychiatric Genetics, NorthShore University HealthSystem, Evanston, Illinois, United States of America; 7 Department of Psychiatry and Behavioral Neuroscience, University of Chicago, Chicago, Illinois, United States of America; The University of Chicago, UNITED STATES OF AMERICA

## Abstract

As more multi-ancestry GWAS summary data become available, we have developed a comprehensive trans-ancestry pathway analysis framework that effectively utilizes this diverse genetic information. Within this framework, we evaluated various strategies for integrating genetic data at different levels—SNP, gene, and pathway—from multiple ancestry groups. Through extensive simulation studies, we have identified robust strategies that demonstrate superior performance across diverse scenarios. Applying these methods, we analyzed 6,970 pathways for their association with schizophrenia, incorporating data from African, East Asian, and European populations. Our analysis identified over 200 pathways significantly associated with schizophrenia, even after excluding genes near genome-wide significant loci. This approach substantially enhances detection efficiency compared to traditional single-ancestry pathway analysis and the conventional approach that amalgamates single-ancestry pathway analysis results across different ancestry groups. Our framework provides a flexible and effective tool for leveraging the expanding pool of multi-ancestry GWAS summary data, thereby improving our ability to identify biologically relevant pathways that contribute to disease susceptibility.

## Author summary

Pathway analysis is a powerful tool used to understand genetic associations with diseases. Instead of looking at individual genetic markers (such as single nucleotide polymorphisms, SNPs), it examines the combined effects of multiple markers within biological pathways. This method is more effective for detecting subtle genetic influences on diseases that might be missed when looking at individual markers alone. Our study expands pathway analysis to include data from diverse ancestry groups, which is often overlooked in traditional single-ancestry genetic studies. We developed a comprehensive trans-ancestry pathway analysis framework to effectively utilize diverse genetic data. In our framework, we explore various strategies for integrating genetic data at different levels—SNP, gene, and pathway—from multiple ancestry groups. Through extensive simulations, we identified robust strategies that perform well in diverse scenarios. Applying these methods, we analyzed around 7,000 pathways for their association with schizophrenia, using data from African, East Asian, and European populations. Our analysis identified over 200 pathways significantly associated with schizophrenia, even after excluding genes near genome-wide significant loci. Our approach significantly improves detection efficiency compared to traditional single-ancestry pathway analysis and the conventional approach that amalgamates single-ancestry pathway analysis results across different ancestry groups. This framework offers a flexible and effective tool for leveraging the growing pool of multi-ancestry GWAS data, enhancing our ability to identify biologically relevant pathways contributing to disease susceptibility.

## Introduction

Genome-wide association studies (GWAS) have successfully identified tens of thousands of single nucleotide polymorphisms (SNPs) linked to complex traits [1–4]. Historically, these studies have predominantly focused on populations of European origin, which limits the generalizability of their findings across global populations and restricts the equitable distribution of health benefits [5,6]. By expanding GWAS to include multi-ancestry populations, we enhance not only the generalizability but also the identification and fine-mapping of disease loci. This expansion deepens our understanding of the interactions between genetic variants and environmental factors across diverse genetic backgrounds, providing comprehensive insights into disease manifestation [7–12].

As GWAS has expanded to encompass multi-ancestry populations, various trans-ancestry (TA) association procedures, focusing primarily on single-SNP analysis, have been developed [13–16]. Conducting TA analysis presents significant challenges due to inherent genetic architecture heterogeneity among ancestral populations, particularly concerning effect size variability [8,17]. This variability arises from the varying direct effects of functional SNPs, potentially influenced by differential environmental interactions, and the uneven marginal effects of tagging SNPs due to population-specific linkage disequilibrium (LD) patterns with the underlying functional variants. There are two general strategies for conducting trans-ancestry single-SNP analysis. The first employs meta-analysis techniques developed to address heterogeneity among studies [18–20]. The second strategy utilizes the global or local genetic differences among populations to model variations in effect sizes [13–16]. Within the second strategy, one approach models the SNP’s marginal effects in different populations as a linear function of key axes of genetic variation, identified through principal component analysis [14]. These axes represent the major directions of genetic variation, capturing the primary population structures that underpin the genetic diversity observed in the data. Another approach models the conditional effects of an SNP—after adjusting for the influence of all other SNPs—as following a joint normal distribution, maintaining a consistent correlation structure throughout the genome [21].

Pathway analysis—or gene set analysis—integrates subtle SNP-level association signals within pathways and has proven effective in identifying the global association between the entire pathway and the outcome [22–30]. This approach allows researchers to detect the cumulative effects of multiple SNPs within a pathway, rather than focusing solely on individual SNP-outcome associations, which are often too weak to be detectable by single-SNP analysis. Despite advancements in single-SNP TA-analysis, pathway analysis remains largely confined to single ancestry GWAS (SA-GWAS), with a notable gap in methodologies tailored for TA-GWAS.

In this report, we propose a suite of TA-pathway analysis approaches based on a flexible premise known as the Trans-Ancestry Gene Consistency (TAGC) assumption. This assumption posits that a specific subset of genes within a pathway is associated with the outcome across various ancestry groups, although the strength of their association may differ across populations due to genetic and environmental variations. The gene-outcome association refers to the overall association signal summarized over the genotyped common SNPs within the gene. This assumption is reasonable, considering that functional variants, especially common ones, are likely shared among diverse populations [8,17,31–33]. This assumption also underpins fine-mapping efforts using multiple ancestry GWAS [34–37]. Even when the functional variant is not directly genotyped, due to tagging SNPs, we would expect a gene containing that functional variant to consistently manifest its association with the outcome across different populations, provided each population has a sufficiently large sample size.

We validated the effectiveness of our methods through extensive simulation studies, which underscore the benefits of our approach across various disease risk models. Additionally, we demonstrated the advantages of our methods by assessing the association of 6,970 pathways with schizophrenia using TA-GWAS summary data from African, East Asian, and European populations.

## Material and methods

### Ethics statement

This study relied on secondary analysis of publicly available summary statistics. Ethical approval for this data was obtained by the primary researchers, and as such no further ethical approval was required for this study.

### Setting and notations

We analyze summary data from *L* single-ancestry GWAS (SA-GWAS), each including *n*^(*l*)^ subjects, *l* = 1,…*L*. For the *l*-th study, we consider summary data for *T* SNPs, represented as $\left\{({\hat{\beta }}_{i}^{\left(l\right)},\tau_{i}^{\left(l\right)}),i=1,\ldots ,T\right\}$. Here, ${\hat{\beta }}_{i}^{\left(l\right)}$ is the estimated coefficient for the association of the *i*-th SNP with the outcome, and $\tau_{i}^{\left(l\right)}$ is the standard error of this estimate. We denote the z-score for the summary data of the *i*-th SNP as $Z_{i}^{\left(l\right)}={\hat{\beta }}_{i}^{\left(l\right)}/\tau_{i}^{\left(l\right)}$, and denote the corresponding p-value as $p_{i}^{\left(l\right)}$. Differences in genotype platforms and filtering criteria across various SA-GWAS can result in missing SNP summary data in some studies. We consider a pathway consisting of *J* genes. In addition to SNP summary data, we assume the availability of reference genomes with individual-level genotype data for each ancestry group. The null hypothesis for the TA-pathway analysis posits that no SNP within the pathway is associated with the outcome across all ancestral populations considered in the study. This is analogous to the self-contained null hypothesis used in the SA-pathway analysis [28].

We assign SNPs to a gene if they are within 50 kb of the gene boundary. A SNP can be assigned to multiple genes. This distance-based SNP-gene assignment rule is commonly adopted by many GWAS analysis procedures [22–24,26,38–40], although other strategies can also be used. In the real data analysis, we will consider an alternative strategy. Our proposed procedures are flexible and can be used with any SNP-gene assignment strategy, as long as the user specifies the assignment.

**Fig 1:**
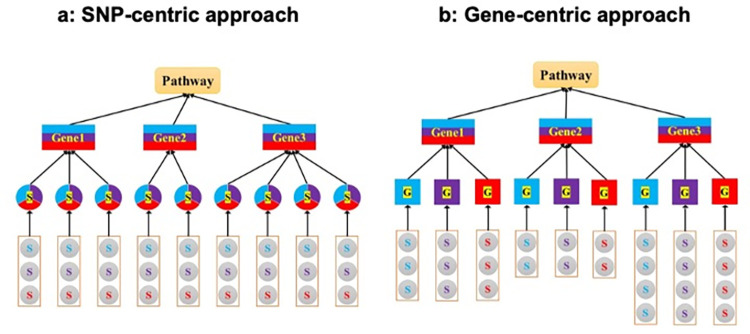
Strategic framework for trans-ancestry pathway analysis. This diagram illustrates two strategies employed in trans-ancestry pathway analysis using GWAS summary data from three distinct populations. The analyzed pathway includes three genes containing 2, 3, and 4 SNPs, respectively. Each population’s GWAS data is color-coded: blue, purple, and red. Trans-ancestry SNP-level and gene-level data are depicted with a mixture of these three colors. (a) SNP-centric approach: SNP-level summary data from the three GWAS (denoted as S) are consolidated to generate trans-ancestry SNP-level p-values. These p-values are then aggregated within each gene to obtain trans-ancestry gene-level p-values. Subsequently, these gene-level p-values are integrated across the genes in the pathway using the Adaptive Rank Truncated Product (ARTP) framework to assess pathway significance. (b) Gene-centric approach: From each GWAS, SNP-level summary data within each gene are consolidated to generate single-ancestry gene-level p-values (G). These p-values are then unified across the three GWAS to form the trans-ancestry gene-level p-value for each gene. Finally, these trans-ancestry gene-level p-values are combined across the pathway using the ARTP framework to determine overall pathway significance.

Our proposed TA-pathway analysis procedures build upon the Adaptive Rank Truncated Product (ARTP) method, a flexible, resampling-based approach initially developed for pathway analysis in SA-GWAS [29,41]. These procedures are categorized by the level at which trans-ancestry genetic data is integrated: SNP-centric, gene-centric, and pathway-centric approaches (Fig 1). In the SNP-centric approach, we consolidate single-ancestry SNP-level (SA-SNP) summary data from multiple SA-GWAS to generate trans-ancestry SNP-level (TA-SNP) summary statistics. These statistics are aggregated to derive trans-ancestry gene-level (TA-gene) summary statistics, which are then combined across the gene set for the pathway analysis using the ARTP framework. The gene-centric approach aggregates SA-SNP summary data within each gene from each SA-GWAS, producing single-ancestry gene-level (SA-gene) summary statistics. These statistics are subsequently unified across different SA-GWAS to form TA-gene summary statistics, using the ARTP framework for the pathway analysis. Finally, the pathway-centric approach integrates p-values from pathway analyses across each SA-GWAS. Below, we first summarize the ARTP method and then detail each of the proposed SNP-centric, gene-centric, and pathway-centric procedures.

### Summary of ARTP

ARTP is designed to aggregate association evidence across multiple correlated components when testing against a global null hypothesis, which asserts that no component is associated with the outcome. In various testing scenarios, these components may be individual SNPs within a gene for gene-based tests or distinct genes within a pathway for pathway-based tests. The ARTP method initiates by aggregating the strongest *c* association signals from all components under consideration, where *c* is a threshold selected from an ordered sequence of candidate values {*c*_*k*_,*k* = 1,…,*K*}, with *c*_1_<⋯<*c*_*K*_. ARTP uses a computationally efficient resampling to the empirical p-value for these aggregated association signals at each predetermined threshold. The pivotal statistic for the final test is the smallest p-value identified among these candidate thresholds (called the minP statistic). In the final step, ARTP repurposes the initially generated samples to evaluate the significance level of the minP statistic, ensuring that the testing procedure is accurately calibrated with a well-controlled Type I error rate.

Here is a summary of the ARTP algorithm:

1. Obtain the association p-value for each component with the outcome and compile them into a vector ***p***_0_ = (*p*_0,1_,*p*_0,2_,…,*p*_0,*q*_).
2. Use a resampling-based procedure to simulate *M* replicas of ***p***_0_ under the global null hypothesis, denoted as $p_{m}=(p_{m,1},p_{m,2},\ldots ,p_{m,q}),m=1,\ldots ,M$.
3. Arrange the elements in ***p***_0_ in ascending order, denoted as *p*_0,(*i*)_,*i* = 1,…,*q*. For each threshold *c*_*k*_, calculate the Negative Log Product (NLP) statistic as
$$w_{0,k}=-\sum_{i=1}^{c_{k}}\log p_{0,(i)},k=1,\ldots ,K.$$ (1)
4. Repeat Step 3 for each resampled ***p***_m_, obtaining their NLP statistics as *w*_*m*,*k*_,*m* = 1,…,*M*,*k* = 1,…*K*.
5. Estimate the empirical p-value for the observed NLP statistic *w*_0,*k*_ as
$$\xi_{0,k}=\frac{\#\{w_{m,k}\geq w_{0,k},m=1,\ldots ,M\}}{M+1},k=1,\ldots ,K.$$ (2)
6. Similarly, estimate the empirical p-value for the resampled NLP statistic *w*_*m*,*k*_ as
$$\xi_{m,k}=\frac{\#\{w_{m^{\prime },k}\geq w_{m,k},m^{\prime }\in \{0,..,M\},and\;m^{\prime }\neq m\}}{M+1},m=1,\ldots ,M,k=1,\ldots ,K.$$ (3)
7. Determine the minimum p-value (minP) statistic for the observed NLP p-values as $T_{0}=\underset{1\leq k\leq K}{\min}\xi_{0,k}$, and for the resampled NLP p-values as $T_{m}=\underset{1\leq k\leq K}{\min}\xi_{m,k},m=1,\ldots ,M$.
8. Finally, estimate the p-value for the minP statistic *T*_0_ as
$$\tau_{0}=\frac{\left\{T_{m}\geq T_{0},m=1,\ldots ,M\right\}}{M+1}$$ (4)

Next, we plan to expand ARTP to facilitate TA-pathway analysis.

### SNP-centric TA-pathway analysis procedures

In this SNP-centric TA-pathway analysis strategy, we begin by aggregating SNP-level summary data from various SA-GWAS to create TA-SNP summary statistics. We then employ the ARTP procedure to integrate these TA-SNP statistics for the final pathway analysis. Although this integration treats TA-SNP statistics as if they were derived from a single SA-GWAS, we have adjusted the resampling algorithm to accommodate the fact that these statistics are compiled from summary data over multiple SA-GWAS.

### Definition of TA-SNP summary statistic

The TA-SNP summary statistic for the *i*-th SNP, denoted as *s*_*i*_, can be constructed in various ways. One commonly employed method is through the inverse variance weighting (IVW) meta-analysis approach, designated as $s_{i}^{IVW}$. This statistic is defined as

$$s_{i}^{IVW}=\sum_{l=1}^{L}\omega_{i}^{\left(l\right)}z_{i}^{\left(l\right)},$$ (5)

where $\omega_{i}^{\left(l\right)}$ is the weight given to the *l-*th study, calculated by

$$\omega_{i}^{\left(l\right)}=\frac{1}{\tau_{i}^{\left(l\right)}\sqrt{\sum_{l=1}^{L}{\left(\frac{1}{\tau_{i}^{\left(l\right)}}\right)}^{2}}}.$$ (6)

In cases where $z_{i}^{\left(l\right)}$ is unavailable, $\omega_{i}^{\left(l\right)}$ defaults to zero. The optimal use of $s_{i}^{IVW}$ is predicated on the assumption of a consistent SNP effect across diverse ancestries. For SNPs exhibiting effect heterogeneity, an alternative summary statistic, $s_{i}^{max}$, is proposed:

$$s_{i}^{max}=\underset{1\leq l\leq L}{\max}\left|z_{i}^{\left(l\right)}\right|.$$ (7)

Since $z_{i}^{\left(l\right)}$ values are independent and normally distributed, we can derive the corresponding p-values for $s_{i}^{IVW}$ and $s_{i}^{max}$ analytically.

Beyond these, we explore methods for combining p-values, which are handy for integrating signals of varying strengths from diverse sources. On classical method is Fisher’s p-value combination. A more recent advancement is the weighted-Fisher (wFisher) method, which amalgamates p-values across studies by adjusting for disparities in sample size[42]. For the p-value $p_{i}^{\left(l\right)}$ associated with $z_{i}^{\left(l\right)}$, we define

$$s_{i}^{wFisher}=\sum_{l=1}^{L}G_{k_{l},2}^{-1}(p_{i}^{\left(l\right)}),$$ (8)

where $G_{k_{l},2}(x)$ denotes the cumulative distribution function of the gamma distribution with a shape parameter $k_{l}=L\frac{n_{l}}{n}$ and a scale parameter set to 2. Here, *n*_*l*_ is the sample size of the *l*-th study, *n* is the total sample size of all *L* studies. In the context of case-control studies, *n*_*l*_ is calculated as the harmonic mean of the number of cases and the number of controls. The resulting statistic, $s_{i}^{wFisher}$, follows a gamma distribution characterized by a shape parameter *L* and a scale parameter of 2.

### Steps for assessing TA-gene p-values

For a gene with *q* SNPs, we select a suitable TA-SNP summary statistic (e.g., *s*^IVW^, *s*^max^, or $s^{wFisher}$) to calculate (*s*_0,1_,*s*_0,2_,…,*s*_0,*q*_) from the observed z-scores $\left(z_{1}^{\left(l\right)},\ldots ,z_{q}^{\left(l\right)}\right)$,*l* = 1,…*L*, across *L* SA-GWAS. The resulting p-values, denoted as ***p***_0_ = (*p*_0,1_,*p*_0,2_,…,*p*_0,*q*_), are introduced as inputs in the initial step of the ARTP procedure. To compute NLP statistics, we choose a set of *K* predetermined SNP-level thresholds *c*_1_<⋯<*c*_*K*_, typically by letting *K* = 2 with thresholds at *c*_1_ = 1 and *c*_2_ = 2.

For the *l*-th SA-GWAS, we use a set of reference genomes, such as those from the 1000 Genomes Project [43], to construct the correlation matrix of SNP genotypes within a gene. This matrix is then used to estimate the variance-covariance matrix *V*^(*l*)^ of the z-score vector $\left(z_{1}^{\left(l\right)},\ldots ,z_{q}^{\left(l\right)}\right)$. Under the global null hypothesis, the z-score vector follows a multivariate normal distribution *N*(0,V^(*l*)^) [24,40]. From each population, we sample *M* replicates of this z-score vector according to (0,*V*^(*l*)^). By pooling these samples from *L* populations, we obtain *M* replicates of TA-SNP summary statistics (*s*_*m*,1_,…,s_*m*,*q*_).*m* = 1,…,*M*, and their corresponding p-values as $p_{m}=(p_{m,1},p_{m,2},\ldots ,p_{m,q}),m=1,\ldots ,M$. This is achieved in Step 2 of the ARTP procedure. Subsequent steps of ARTP utilize the outcomes of the two initial phases to determine the TA-gene p-value *τ*_0_. Furthermore, as in Step 8, the p-value for the resampled minP statistic *T*_*m*_ can be estimated as

$$\tau_{m}=\frac{\#\{\tau_{m^{\prime }}\geq \tau_{m},m^{\prime }\in \{0,\ldots ,M\},m^{\prime }\neq m\}}{M+1},m=1,\ldots ,M.$$ (9)

These values serve as simulated instances of *τ*_0_ under the null hypothesis, providing a basis for the pathway-level analysis.

The aforementioned procedure requires generating *M* samples of the z-score vector for each ancestral population based on their respective multivariate normal distributions—a process that can be computationally intensive, especially when performing TA-pathway analysis with three or more SA-GWAS. This process can be simplified when *s*^IVW^ is used to compute TA-SNP summary statistics, as their covariance matrix under the null hypothesis can be directly estimated. Specifically, for any two correlated SNPs *i* and *i*′, we have

$$cov(s_{i}^{IVW},s_{i^{\prime }}^{IVW})=\sum_{l=1}^{L}\omega_{i}^{\left(l\right)}\omega_{i^{\prime }}^{\left(l\right)}cov(z_{i}^{\left(l\right)},z_{i^{\prime }}^{\left(l\right)}),$$ (10)

where $cov(z_{i}^{\left(l\right)},z_{i^{'}}^{\left(l\right)})$ is estimated from the empirical correlation coefficient of their genotypes observed in the *l-*th population’s reference genomes. With this covariance matrix, we can directly generate TA-SNP summary statistics (*s*_*m*,1_,…,*s*_*m*,*q*_), *m* = 1,…,*M*, and proceed with the remaining steps of the ARTP procedure.

### Steps for assessing TA-pathway analysis p-value

The procedure outlined in the previous section is applied to each of the *J* genes within the pathway to obtain their TA-gene p-values, designated as *τ*_0,*j*_, *j* = 1,…,*J*. The corresponding resampled counterparts under the null distribution obtained by (9) are defined as *τ*_*m*,*j*_,*m* = 1,…,*M*, *j* = 1,…,*J*. To account for potential correlation between z-scores for SNPs across different genes within the *l*-th population, we jointly generate z-scores for SNPs in all correlated genes using a multivariate normal distribution *N*(0,*V*^(*l*)^), where *V*^(*l*)^ is the estimated variance-covariance matrix for these SNPs’ z-scores in the *l*-th population. This approach enables the concurrent assessment of TA-gene p-values *τ*_0,*j*_ for correlated genes and *τ*_*m*,*j*_ for the *m-*th generated z-score vector.

To apply the ARTP procedure for the final assessment of pathway-outcome association, we set ***p***_0_ = (*τ*_0,1_,*τ*_0,2_,…,*τ*_0,*J*_) in Step 1, and for Step 2, ***p***_*m*_ is set as (*τ*_*m*,1_,*τ*_*m*,2_,…,*τ*_*m*,*J*_) *m* = 1,…,*M*. In Step 3, we employ a set of *K*′ gene-level thresholds $d_{1}<\cdots <d_{K^{\prime }}$ to compute the NLP statistics. We recommend setting *K*′ = 10 and $d_{k}=kmax\left(1,\lceil \frac{J}{20}\rceil \right),k=1,\ldots ,10$. Subsequent steps are executed as prescribed within the ARTP framework to derive the final p-value for the TA-pathway analysis. The optimal threshold $d_{k^{\prime }}$, where $\xi_{0,k^{\prime }}=\underset{1\leq k\leq K^{\prime }}{\min}\xi_{0,k}$, identifies the subset of genes that collectively contribute the most significant pathway assocation signal. These genes may serve as valuable candidates for further research.

Depending on which TA-SNP statistic is used, the corresponding SNP-centric pathway analysis procedures are termed SNP-IVW, SNP-max, and SNP-wFisher. Each strategy is suited to specific scenarios: SNP-IVW is effective when different ancestry groups share functional SNPs with similar effects, while SNP-wFisher and SNP-max are tailored for situations involving different functional SNPs or varying effects across groups.

### Gene-centric TA-pathway analysis procedures

In this gene-centric strategy, we first obtain SA-gene p-values within each SA-GWAS using ARTP. For each gene, these p-values are then integrated across all SA-GWAS to compute the TA-gene p-values. These TA-gene p-values serve as the foundation for the subsequent TA-pathway analysis.

For the *l*-th SA-GWAS, we apply ARTP to obtain its SA-gene p-values $\tau_{0,j}^{\left(l\right)},j=1,\ldots ,J$, along with *M* simulated replica $\tau_{m,j}^{\left(l\right)},m=1,\ldots ,M,j=1,\ldots ,J$. To synthesize TA-gene p-values across various SA-GWAS, we consider the weighted Fisher’s method and the minimum p-value approach, with the latter using the smallest SA-gene p-value across populations for subsequent pathway analysis. Using the chosen method, we combine $\tau_{0,j}^{\left(1\right)},\ldots ,\tau_{0,j}^{\left(L\right)}$ for gene *j* and obtain its TA-gene p-value *τ*_0,*j*_. In parallel, we use the same method to synthesize $\left(\tau_{m,j}^{\left(1\right)},\ldots ,\tau_{m,j}^{\left(L\right)}\right)$ to form *τ*_*m*,*j*_, the TA-gene p-value for the *m*-th simulated replica, wiht *m* = 1,…,*M*.

For the final TA-pathway analysis, we employ the ARTP framework once more, initializing ***p***_0_ as (*τ*_0,1_,*τ*_0,2_,…,*τ*_0,*J*_) in Step 1. For Step 2, ***p***_*m*_ is specified as (*τ*_*m*,1_,*τ*_*m*,2_,…,*τ*_*m*,*J*_), for *m* = 1,…,*M*. The subsequent steps of the ARTP procedure are then carried out routinely to obtain the TA-pathway p-value. Similar to the SNP-centric procedure, the threshold $d_{k^{\prime }}$, where $\xi_{0,k^{\prime }}=\underset{1\leq k\leq K^{\prime }}{\min}\xi_{0,k}$, can be used to identify the most significant subset of genes.

Depending on the method used to derive the TA-gene p-value, we refer to the final procedure as Gene-wFisher or Gene-minP. Under the TAGC assumption, Gene-wFisher is preferred over Gene-minP, as it better aligns with the expectation that a gene is either unrelated or consistently associated with the outcome across each of the populations considered.

### Pathway-centric and composite TA-pathway analysis procedures

One natural pathway-centric strategy is to combine SA-pathway analysis p-values (based on ARTP) across different ancestry populations using the weighted Fisher’s method, referred to as Path-joint. Path-joint is expected to be particularly suitable for scenarios where the TAGC assumption is markedly contravened, such as settings where there are no overlapping causal genes across different ancestry groups. However, as noted in the Introduction, such situations are unlikely to occur in practice.

Additionally, we can employ the Aggregated Cauchy Association Test (ACAT) to construct a composite test that integrates p-values from various pathway analysis methods. The ACAT procedure offers a flexible framework for combining p-values from correlated statistical tests through an analytical formula [44]. This formula evaluates the tail-end distribution of the composite statistic, effectively bypassing the need for computationally intensive permutation procedures. We consider ACAT-IVW-wFisher, which applies ACAT to merge results from the SNP-centric SNP-IVW and the gene-centric Gene-wFisher procedures. Designed to leverage the strengths of both SNP-IVW and Gene-wFisher, ACAT-IVW-wFisher is expected to deliver consistent and robust performance across diverse settings. SNP-IVW is advantageous when all considered ancestry groups share identical functional SNPs with similar effects, whereas Gene-wFisher is likely effective under the broader TAGC assumption. However, a notable limitation of the ACAT test is its inability to provide insights into which specific genes contribute to the detected pathway association, as it only yields the final pathway association p-value without detailed gene-level analysis. To obtain specific gene-level signals, we must still rely on results from Gene-wFisher and SNP-IVW.

## Results

### Simulation study designs

In our simulation study, we used simulated genotype data with realistic LD patterns from five continental populations—African (AFR), American (AMR), East Asian (EAS), European (EUR), and South Asian (SAS). This data, comprising approximately 19.2 million SNPs for 120,000 individuals per population [41], was generated in alignment with the 1000 Genomes Project [43]. These simulated subjects served as the source population for each ancestry group under consideration. Our analysis focused on a pathway involving 100 genes located on chromosome 21, with the assumption that genotypes on SNPs from distinct genes are independent within each study population.

We considered a pathway analysis consisting of five case-control studies, each drawn from one of the five continental populations. We specified their sample sizes as follows: 4,000 from AFR, 6,000 from AMR, 6,000 from EAS, 10,000 from EUR, and 4,000 from SAS, with an equal number of cases and controls. Additionally, we extracted a reference set of 500 samples from each population. Our simulations focused on relatively common SNPs, with minor allele frequencies (MAF) exceeding 1%, within each population. It should be noted that SNP sets analyzed from each population are not identical, as an SNP considered common in one population may be rare in another. Upon generating the genotypes for cases and controls, we applied a standard logistic regression model to each SNP to derive summary statistics, which, along with the reference samples, were used for the pathway analysis.

To assess the Type I error rate of all considered procedures, we randomly drew genotype data from each continental population, assigning it to cases and controls within each case-control study. We generated and analyzed 10,000 replicates of these studies across five continental populations, applying the designated pathway analysis procedures to their summary data. Besides binary outcome, we also conducted similar simulation studies for a continuous outcome, maintaining the same sample size configuration as used in the binary outcome simulation study.

For the power evaluation, we considered a general binary disease model in our simulation studies. For the *l-*th population, where *l* = 1 to 5 corresponds to AFR, AMR, EAS, EUR, and SAS respectively, out of the 100 genes in the considered pathway, we hypothesized that the disease risk was modulated by a subset of 10 causal genes, indexed by *R*^(*l*)^, each harboring a single functional SNP. The disease risk model for *l-*th population was given as,

$$logit[Pr(Y=1|G^{\left(l\right)})]=\alpha^{\left(l\right)}+\sum_{f\in R^{\left(l\right)}}\beta^{\left(l\right)}g_{f}^{\left(l\right)},$$ (11)

where $g_{f}^{\left(l\right)}$ represents the genotype for the functional SNP within the causal gene *f*∈*R*^(*l*)^. In this model, we assumed a consistent effect size, *β*^(*l*)^, for all functional SNPs within each population. The intercept *α*^(*l*)^ was calibrated to reflect a low disease prevalence in the *l*-th population.

We evaluated the power of our proposed methods across four risk model settings: one Common Risk Model and three Distinct Risk Models 1, 2, and 3.

Under the Common Risk Model setting, we assumed all five continental populations shared the same disease risk model. Specifically, this entailed an identical set of causal genes (i.e., *R*^(*l*)^ = {1,2,…,10}*l* = 1,…5), the same functional SNPs centrally located within each of these causal genes, and a uniform effect size (*β*^(*l*)^ = 0.06,*l* = 1,…5), applied across the five populations.

In each of the three Distinct Risk Model settings, every study population followed its unique disease risk model. In Distinct Risk Model 1, the risk models for the five populations shared the same set of causal genes and the same set of functional SNPs, yet they exhibited varied effect sizes, with *β*^(*l*)^ = -0.084, -0.06, -0.06, 0.06, and 0.084 for *l* = 1,…,5, respectively. In Distinct Risk Model 2, while five risk models shared the same set of causal genes, they had different functional SNPs in each causal gene, maintaining the same effect size configuration as in Distinct Risk Model 1. Lastly, Distinct Risk Model 3 introduced a unique set of causal genes for each population ($R^{\left(1\right)}=\{1,2,\ldots ,10\},R^{\left(2\right)}=\{6,7,\ldots ,13\},R^{\left(3\right)}=\{7,8,\ldots ,16\},R^{\left(4\right)}=\{10,11,\ldots ,19\}$, and *R*^(5)^ = {13,14,…,22}). In this model, even when populations shared a causal gene, they had different functional SNPs within that gene, with effect sizes following the same configuration as in Distinct Risk Model 1. Settings under Distinct Risk Models 1 and 2 adhered to the TAGC assumption, as they utilized the same set of functional genes across each population. However, Distinct Risk Model 3 deviated from the TAGC assumption by introducing a partially overlapping set of causal genes, where each population had some unique genes but also shared some with others.

To evaluate the power of the proposed methods under each of the four settings, we simulated 2000 datasets for pathway analysis. Each dataset included five case-control studies, one from each continental population, with the previously mentioned sample sizes. Genotypes for each gene within these studies were generated using the algorithm given by [29], using the simulated genome data provided by [41]. Within each setting, we examined two distinct scenarios: the first assumed that genotypes at the functional SNPs were measured and available for analysis, while the second, more realistic scenario, dealt with situations where genotypes at these functional SNPs were inaccessible, either because they were not measured or were excluded during LD filtering processes.

Furthermore, we conducted two additional series of simulation studies under the four previously described risk models, with some modifications to ensure that the power of the procedures remained within a reasonable range. These simulations considered a pathway comprising 100 genes, including either 20 or 40 causal genes, in contrast to the initial set of 10 causal genes, as detailed in S1 Text.

### Simulation results

In Table 1, we show the performance of various procedures at the nominal Type I error rate of 0.05, for both the binary and continuous outcome. These findings confirm that all procedures properly maintain their Type I errors.

**Table 1:** Assessment of Type I error rates across pathway analysis procedures using 10,000 simulated datasets.

	Binary Outcome		Continuous Outcome	
Method	alpha = 0.05	alpha = 0.01	alpha = 0.05	alpha = 0.01
Path-AFR	0.047	0.010	0.051	0.010
Path-AMR	0.043	0.009	0.052	0.011
Path-EAS	0.047	0.009	0.050	0.009
Path-EUR	0.049	0.009	0.048	0.009
Path-SAS	0.044	0.009	0.053	0.011
Path-joint	0.046	0.009	0.049	0.010
Gene-wFisher	0.045	0.009	0.052	0.010
Gene-minP	0.048	0.009	0.050	0.011
SNP-wFisher	0.049	0.010	0.055	0.010
SNP-max	0.046	0.010	0.051	0.011
SNP-IVW	0.046	0.009	0.052	0.012
ACAT-IVW-wFisher	0.049	0.009	0.056	0.012

Note: Path-AFR, Path-AMR, Path-EAS, Path-EUR, and Path-SAS refer to single-ancestry (SA) pathway analyses of African, American, East Asian, European, and South Asian GWAS, respectively. Path-joint represents a meta-analysis of SA-pathway analysis results. Gene-wFisher and Gene-minP are gene-centric trans-ancestry (TA) pathway analyses utilizing the weighted Fisher’s method and the minimum p-value approach, respectively. SNP-centric TA-pathway analyses are represented by SNP-wFisher, SNP-max, and SNP-IVW, which are based on the weighted Fisher’s method, the maximum of absolute z-score values, and the inverse variance weighting method, respectively. ACAT-IVW-wFisher is a composite test that combines results from SNP-IVW and Gene-wFisher.

**Fig 2:**
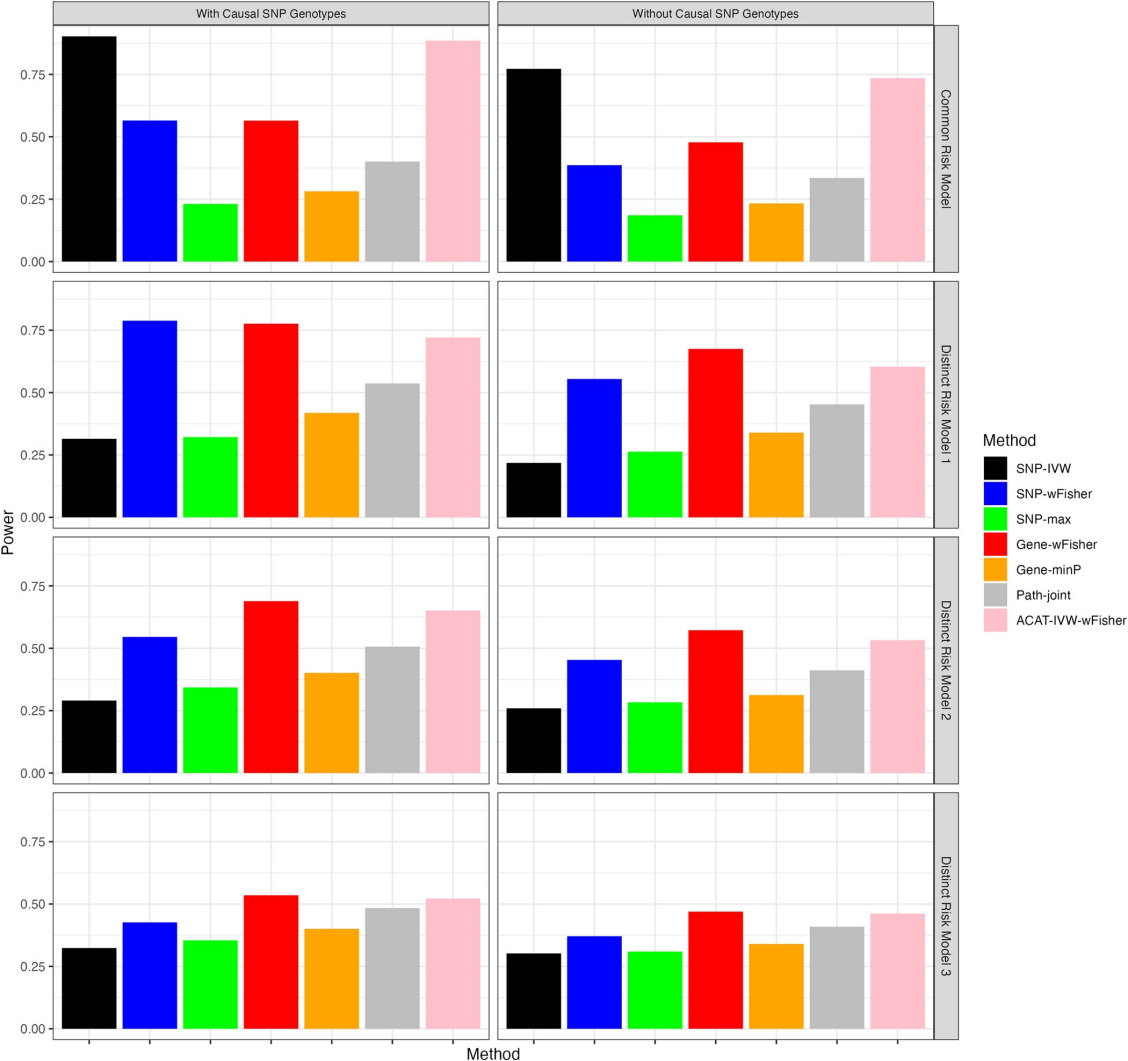
Power comparisons for pathway analyses of a 100-gene pathway with 10 causal genes and a 1:1 case-control ratio. Power is estimated from 2,000 replicates at a type I error rate of 0.05. Detailed method descriptions can be found in the footnotes of Table 1.

Fig 2 presents power comparison results within the setting featuring 10 causal genes within the considered pathway. Under the Common Risk Model, where genotypes at functional SNPs are accessible, Fig 2 shows that the SNP-centric approach, SNP-IVW, significantly outperforms other gene-centric and SNP-centric methods. This superiority is expected as SNP-IVW employs summary statistics from various ancestry-specific populations through the inverse variance-weighted method, which is optimal when SNP effect sizes are consistent across populations. Moreover, even when genotypes at functional SNPs are unavailable, SNP-IVW maintains its efficacy. Notably, under the Common Risk Model, the composite test ACAT-IVW-wFisher achieves performance comparable to SNP-IVW in both scenarios.

Across the three Distinct Risk Model settings, Gene-wFisher and ACAT-IVW-wFisher consistently demonstrate robust performance, particularly when genotypes at functional SNPs are unavailable. In Distinct Risk Model 1, SNP-wFisher demonstrates similar performance to Gene-wFisher when genotypes for shared functional SNPs are available. However, in scenarios where genotypes are not accessible—and in Distinct Risk Models 2 and 3, which involve different sets of functional SNPs—SNP-wFisher’s performance noticeably lags behind that of Gene-wFisher and ACAT-IVW-wFisher. In Distinct Risk Model 3, where the TGAC assumption is violated due to partially overlapping sets of functional genes between populations, Gene-wFisher and ACAT-IVW-wFisher show a slightly advantage over Path-joint.

To evaluate the impact of unequal case-to-control ratios on statistical power, we conducted a new set of simulations with different case and control sizes across each ancestry population. Specifically, the number of cases was set to 1,100 for AFR and SAS, 1,650 for AMR and EAS, and 5,000 for EUR. For each population, the number of controls was set to ten times the number of cases, except for EUR, where the control size matched the case size. As shown in Fig A in S1 Text, the observed power levels of all methods are similar to those in Fig 2, where each GWAS had an equal number of cases and controls. This similarity was anticipated, as both sets of GWAS for each ancestry population had the same effective sample size, calculated as four times the harmonic mean of the number of cases and the number of controls. It is well-established that in a simple logistic regression model with a single risk factor, the power to detect the factor—assuming a constant effect size—is directly proportional to the effective sample size. Our findings confirm that this principle holds true in more complex pathway analyses, reinforcing the importance of effective sample size as a critical determinant of statistical power.

Simulation results for pathways containing 20 and 40 causal genes are presented in Figs B and C in S1 Text. These results support similar conclusions to those drawn from the pathway with 10 causal genes. In summary, our simulation studies have demonstrated that the efficacy of pathway analysis methods is heavily influenced by the inherent risk models present across various populations. In practical settings, where the complete measurement of genotypes for functional SNPs may not be feasible, and considering the diversity in risk models across populations, we recommend the combined use of Gene-wFisher and SNP-IVW methods to address a wide range of practical scenarios effectively. Additionally, we advocate for the use of the composite test, ACAT-IVW-wFisher, as a formal method to integrate the strengths of both approaches, thereby enhancing the robustness and comprehensiveness of the analysis.

### Real data analysis

We performed pathway analyses on multi-ancestry GWAS summary data for schizophrenia [45]. The GWAS summary data was accessed from the Psychiatric Genomics Consortium (PGC) website, comprising 53,386 cases and 77,258 controls of European ancestry, 14,004 cases and 16,757 controls of East Asian ancestry, and 6,152 cases and 3,918 controls of African ancestry. Reference genomes from the 1000 Genomes Project included 503 European, 504 East Asian, and 661 African samples. To uncover novel signals and avoid results being dominated by well-established loci, from each GWAS, we excluded SNPs with genome-wide significant p-values (i.e., p-value less than 5×10^−8^), as well as their neighboring SNPs within a 500 kb radius. Additionally, we adjusted for population stratification by rescaling the variance of the coefficient estimates using the inflation factor *λ*. The values for *λ* were 1.7 for Euroepan, 1.2 for East Asian, and 1.04 for African GWAS, respectively.

Our analysis encompassed a total of 6,970 pathways from the C2 curated gene sets in the Molecular Signatures Database (MsigDB) [46], which includes 1,632 REACTOME pathways [47]. After filtering and merging with the schizophrenia GWAS data, the median number of genes in a pathway is 27, and the 75th percentile is 61. We limited our analysis to pathways containing fewer than 500 genes. The results of the pathway analysis across all methods are summarized in S1 Table. For each method, we applied a global significance threshold of 7.17×10^−6^, as determined by the Bonferroni correction for multiple testing. For each SA-GWAS, we performed pathway analysis using ARTP, identifying 55 significant pathways in the European GWAS (Path-EUR), 11 in the East Asian GWAS (Path-EAS), and none in the African GWAS (Path-AFR). As expected, the number of significant pathways detected correlated with the effective sample size of each SA-GWAS.

In the TA-pathway analysis, various procedures yielded highly consistent results when comparing the log-transformed p-values. For instance, the Pearson correlation coefficients for these log p-values were 0.95 between Gene-wFisher and Path-joint, 0.92 between Gene-wFisher and SNP-IVW, and 0.89 between Path-joint and SNP-IVW. However, due to differences in their statistical power, these methods identified varying numbers of significant pathways. The SNP-centric method, SNP-IVW, detected 179 significant pathways, while the gene-centric approach, Gene-wFisher, identified 207 significant pathways. The pathway-centric approach, Path-joint, found 125 significant pathways. Notably, ACAT-IVW-wFisher, which integrates the strengths of both SNP-centric and gene-centric approaches, identified 214 significant pathways, slightly outperforming Gene-wFisher (214 vs. 207).

**Fig 3:**
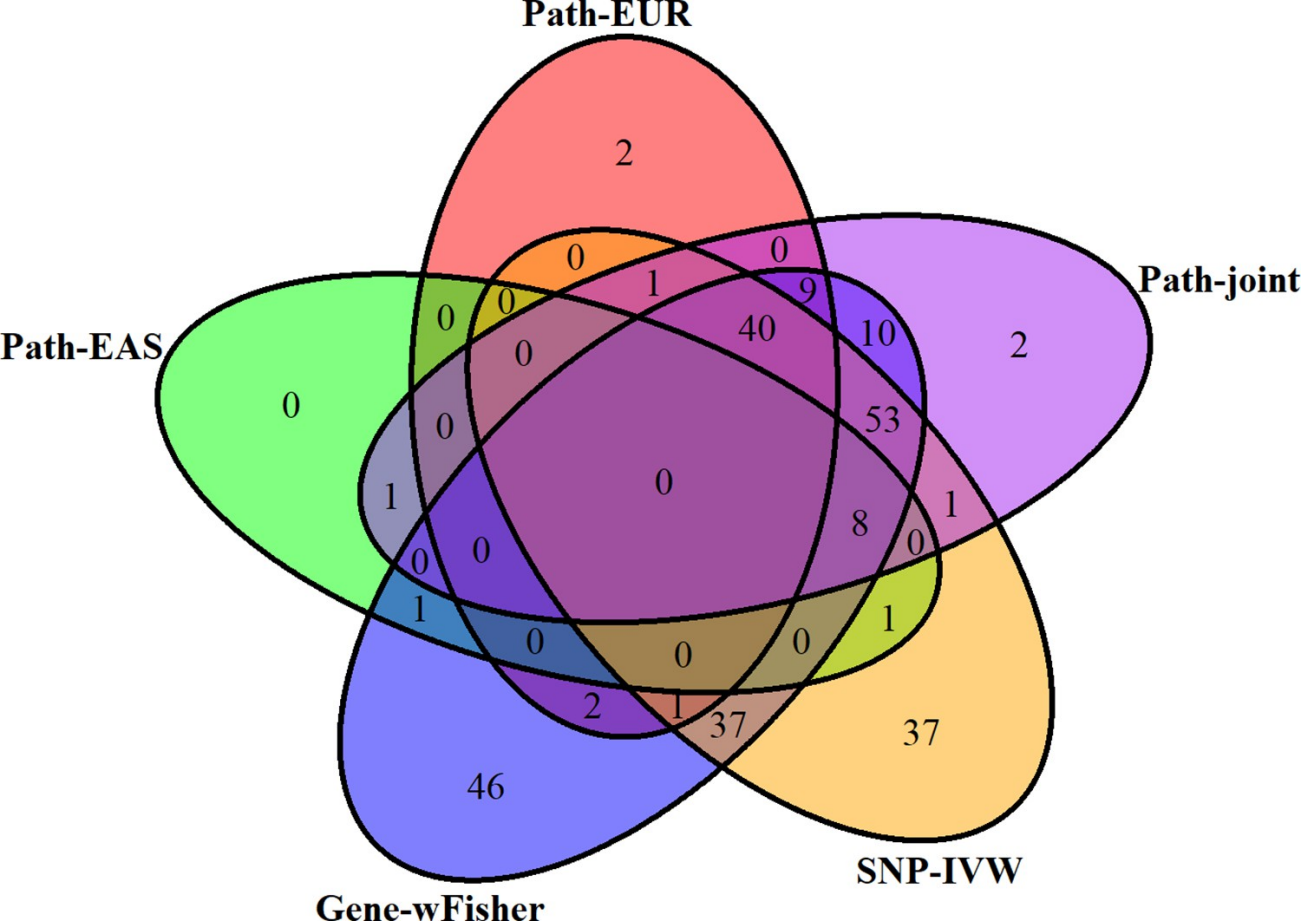
Venn diagram comparing significant pathways associated with schizophrenia identified by five different pathway analysis methods. The global significance threshold is established at 7.17×10^−6^, calculated using the Bonferroni correction to account for multiple testing of 6,970 pathways. Detailed method descriptions can be found in the footnotes of Table 1.

In Fig 3, we illustrate the interrelationships among the sets of significant pathways identified by five pathway analysis approaches (Path-EAS, Path-EUR, Gene-wFisher, SNP-IVW, and Path-joint) using a Venn diagram. Notably, the Gene-wFisher and SNP-IVW methods complement each other, each identifying over 40 unique pathways not detected by the other. Combined, these two methods identified 247 unique pathways, encompassing all 214 pathways detected by ACAT-IVW-wFisher. Additionally, among the 125 significant pathways uncovered by Path-joint, 120 are also identified by Gene-wFisher.

**Fig 4:**
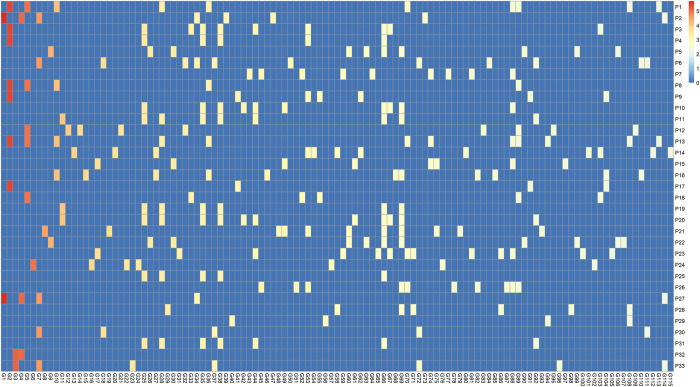
Heatmap of gene-level p-values for selected genes across 33 significant REACTOME pathways associated with schizophrenia detected by the Gene-wFisher method. This heatmap displays gene-level p-values for 115 unique genes, as detailed in S3 Table, across 33 significant REACTOME pathways listed in S2 Table. Each gene, with a p-value below 0.005 as estimated by the Gene-wFisher method, is featured on the x-axis, while pathways are displayed on the y-axis, organized by their respective p-values. Each row in the heatmap corresponds to one significant pathway, with color intensity of each cell reflecting the gene-level p-value on a -log_10_ scale. Cells for genes not included in a pathway are shaded blue.

To facilitate interpretation, we concentrated on significant pathways within the REACTOME database. We identified 37 significant REACTOME pathways using either the Gene-wFisher or SNP-IVW methods (Figs D-AN in S1 Text). The heatmap for the 33 pathways identified by Gene-wFisher is shown in Fig 4. In this heatmap, rows represent pathways ordered by their p-values as provided by Gene-wFisher, while columns represent genes that are included in at least one of these significant pathways and have gene-level p-values less than 0.005. S2 and S3 Tables list the pathways and genes shown in Fig 4. Similarly, the heatmap for the 23 significant pathways detected by SNP-IVW is shown in Fig AO in S1 Text, with corresponding pathways and genes detailed in S4 and S5 Tables.

Among these 37 pathways, ten are associated with the Signal Transduction group as defined by REACTOME. This group is crucial for enabling cells to perceive and respond to internal and external stimuli, facilitating communication within and between cells. An example of these pathways is the Rho GTPase cycle pathway (Fig AF in S1 Text), which includes genes essential for the regulation and activation of Rho GTPases. Studies have demonstrated that Rho GTPases play critical roles in neuronal development, structural plasticity, and cytoskeletal dynamics—processes that are frequently disrupted in schizophrenia. Importantly, several genetic variants in the Rho GTPase cycle pathway are significantly associated with schizophrenia, and experimental models have shown that mice carrying mutations in genes such as Arhgap10 from this pathway exhibit cognitive deficits and morphological abnormalities relevant to schizophrenia [48,49].

Furthermore, nine significant pathways belong to the REACTOME top-level groups: Metabolism, Metabolism of Protein, and Metabolism of RNA. These groups are crucial for the synthesis, modification, and breakdown of vital biomolecules necessary for cellular growth, maintenance, and energy production. Lipid metabolism abnormalities in schizophrenia and other neuropsychiatric disorders have emerged as a mechanism contributing to disease risk [50]. For instance, a concerted synaptic neuron and astrocyte program (SNAP) decline involving abnormal cholesterol synthesis has recently been implicated in aging and schizophrenia [51]. One of the nine detected pathways is the metabolism of carbohydrate pathway (Fig W in S1 Text), which comprises genes encoding enzymes essential for complex carbohydrate metabolic processes. Evidence suggests a strong link between schizophrenia and carbohydrate metabolism [52]. This connection likely stems from imbalances between oxidative and antioxidant processes that impair the brain’s energy production capacity, contributing to early schizophrenia symptoms. The pivotal role of carbohydrate metabolism dysregulation in schizophrenia’s pathophysiology is further supported by a study showing altered metabolite levels linked to lipid and energy metabolism in patients with schizophrenia [53].

Additionally, six pathways within the Cell Cycle group were identified. This group encompasses REACTOME pathways critical for cell progression through various phases of the cell cycle, including G1, S, G2, and M. Noteworthy among these is the Cell Cycle Checkpoints pathway, which comprises a network of 243 genes responsible for monitoring and regulating the cell cycle at specific checkpoints (Fig I in S1 Text). A search through the Schizophrenia Gene Resource (SZGR 2.0) database [54] revealed that more than 68 of these genes are listed, indicating a notable enrichment of schizophrenia-relevant genes in this pathway.

Other significant pathways fall under REACTOME’s top-level groups such as Chromatin Organization, Immune System, and Neuronal System. For example, the chromatin-modifying enzymes pathway (Fig L in S1 Text), crucial for epigenetic alterations particularly in histone methylation, has been linked to schizophrenia through dysfunctions in enzymes like histone methyltransferases and demethylases. These disturbances have been significantly associated with the disease in multiple GWAS analyses, highlighting their role in its development and progression [55–58]. Moreover, the antigen processing ubiquitination proteasome degradation pathway (Fig D in S1 Text), an essential component of the ubiquitin-proteasome system, plays a key role in the targeted degradation of proteins. Research has shown that antipsychotic medications modulate the expression of specific proteins within this pathway, such as PSMD12, UBFD1, and COPS8. Disruptions in this system can lead to the accumulation of damaged or dysfunctional proteins, contributing to the pathogenesis of schizophrenia [59].

In the analyses described above, we assigned SNPs to their respective genes if they were within 50 kb of the gene boundary. However, this distance-based SNP-gene assignment strategy may not capture certain SNPs that influence gene expression from more than 50 kb away. To address this limitation, we employed an alternative SNP-gene assignment strategy that leverages tissue-specific genotype-expression relationships [60].

For our schizophrenia study, we utilized pre-established genotype-expression models derived from GWAS and cortex-specific gene expression data from the Genotype-Tissue Expression (GTEx) project [61]. Given the limited availability of cortex tissue samples (n = 205), the database provided only 6073 genes whose expression could be reliably predicted by their respective sets of SNPs, each with heritability estimate p-values below 0.01. These gene expression-based SNP-gene assignments were then incorporated into our pathway analysis procedures, enabling a focused reevaluation of each pathway based solely on the 6073 identified genes.

This alternative approach proved less effective than the distance-based strategy. For instance, the ACAT-IVW-wFisher method identified 214 significant pathways using the distance-based rule, but only 64 pathways (with 30 overlapping) under the gene expression-based assignment rule. Similarly, SNP-IVW identified 179 versus 71 pathways, and Gene-wFisher identified 207 versus 38 pathways using the two different assignment rules. Detailed results are available in S6 Table. The primary limitation is the restricted set of genes available for pathway analysis, which may become more impactful as more data from the cortex becomes available.

## Discussion

We have developed a comprehensive framework for conducting pathway analysis using summary data from multi-ancestry GWAS. Within this framework, we evaluated various TA-pathway analysis strategies, including SNP-centric, gene-centric, and pathway-centric approaches. Through extensive simulation studies, we found that, within the SNP-centric approaches, SNP-IVW, and within the gene-centric approaches, Gene-wFisher, are particularly effective at detecting pathway associations under certain conditions. The composite approach, ACAT-IVW-wFisher, which integrates results from SNP-IVW and Gene-wFisher, demonstrates the most robust performance across a wide range of underlying phenotype models where the TAGC assumption holds or is partially met. We applied these new procedures to analyze multi-ancestry GWAS data on schizophrenia, detecting significantly more pathways than traditional methods.

Our analysis identified 37 significant REACTOME pathways associated with schizophrenia. Among these, ten pathways involve signal transduction, essential for cells to respond to environmental changes through internal and intercellular signaling. Nine pathways belong to the metabolism process, crucial for synthesizing, modifying, and breaking down vital biomolecules necessary for cellular functions and energy production. Additionally, six pathways pertain to the cell cycle, which is integral for the proper progression and division of cells. We also identified significant pathways related to chromatin organization and the immune and neuronal systems. As discussed in the Real Data Analysis Section, a number of those pathways are supported by additional evidence linking them to schizophrenia. Beyond those REACTOME pathways, our analysis also detected over 100 other pathways from various sources, including those from KEGG. Notably, the KEGG Axon Guidance Pathway (p-value = 2.25×10^−7^ by ACAT-IVW-wFisher), critical for neuron development and synaptic function, is pivotal in understanding schizophrenia pathology. These findings enhance our understanding of the biological underpinnings of schizophrenia.

While the proposed TA-pathway analysis methods are tailored for scenarios where the TAGC assumption is generally valid, as evidenced in the Distinct Risk Model 3 setting, both ACAT-IVW-wFisher and Gene-wFisher remain effective even when this assumption is not fully met. In extreme cases, such as when no overlapping causal genes exist across different ancestry groups, the pathway-centric method, Path-joint, appears to be more appropriate. For instance, in a simulation resembling Distinct Risk Model 3 but with completely distinct gene sets for each population, Path-joint slightly outperformed ACAT-IVW-wFisher. Although it is feasible to further enhance the composite test by incorporating results from Path-joint, along with those from SNP-IVW and Gene-wFisher for even more robust performance, we believe such modifications are generally unnecessary given the overall reliability of the TAGC assumption in typical applications.

Our procedures are nonparametric in nature, as they do not rely on any underlying model for the trait under study. On the other hand, the linear mixed model has become a widely adopted tool for modeling the collective effects of genetic variants on complex traits. This model has been extended to jointly model the polygenic effects across multi-ancestry populations, establishing a foundation for trans-ancestry single-SNP analysis. Moreover, it provides a mechanism to enhance single-ancestry single-SNP analysis in underrepresented populations (e.g., South Asian) by leveraging large-scale GWAS data from well-studied ancestry populations (e.g., European). The linear mixed model framework is also suitable for developing TA-pathway analysis procedures. However, this transition to a model-based approach introduces several unknown parameters, most critically the variance-covariance matrix of SNP effect sizes across different populations. These matrices are typically assumed to be consistent or to exhibit a local structure. The success of TA-pathway analysis procedures that utilize this model hinges on the precise estimation of these parameters. Therefore, further investigations are crucial to assess the robustness of such parametric procedures in pathway analysis settings, focusing on their sensitivity to the underlying model assumptions and errors in parameter estimation, and examining how these factors affect their performance across diverse genetic backgrounds.

In our TA-pathway analysis, we utilize testing statistics designed for the self-contained null hypothesis. Another commonly used null hypothesis is the competitive null hypothesis, which asserts that genes within the pathway are no more associated with the outcome than those outside it. The choice of the most appropriate null hypothesis for genomic studies remains a subject of debate [62–64]. Recently, a novel null hypothesis was proposed that integrates the self-contained and competitive hypotheses. This unified hypothesis stipulates that the proportion of truly associated genes within a pathway must be less than a certain threshold, *c*, which can be determined post hoc [65]. Given the merits of different null hypotheses, it would be advantageous to develop TA-pathway analysis procedures tailored to each hypothesis and assess the performance of SNP-centric, gene-centric, and pathway-centric strategies within these varied frameworks. This area represents a promising direction for future research.

In summary, we have developed a suite of flexible procedures for TA-pathway analysis. Building upon the original ARTP2, which was designed for SA-pathway analysis, we have expanded its capabilities to include these new procedures. The upgraded package, now called ARTP3, supports both SA-pathway and TA-pathway analyses through a user-friendly interface. As multi-ancestry GWAS data become increasingly available, we anticipate that ARTP3 will prove to be an invaluable tool for exploiting data from diverse populations to identify pathways that contribute to disease susceptibility.

## Supporting information

S1 Text Supplementary notes and figures. (DOCX)

S1 Table Results of pathway analyses across 6,970 pathways. (XLS)

S2 Table Thirty-three significant REACTOME pathways identified by the Gene-wFisher method. (XLS)

S3 Table One hundred fifteen genes with gene-level P-values below 0.005 in the thirty-three significant REACTOME pathways identified by the Gene-wFisher method. (XLS)

S4 Table Twenty-three significant REACTOME pathways identified by the SNP-IVW method. (XLS)

S5 Table Ninety-one genes with gene-level P-values below 0.005 in twenty-three significant REACTOME pathways identified by the SNP-IVW method. (XLS)

S6 Table Results of pathway analyses across 6,476 pathways using SNP-gene assignments derived from GTEx gene expression models. (XLS)
