# Supplementary material for: A comprehensive framework for trans-ancestry pathway analysis using GWAS summary data from diverse populations
Source: PLoS Genet. 2024 Oct 23;20(10):e1011322. doi: 10.1371/journal.pgen.1011322 (PMC11534268; doi:10.1371/journal.pgen.1011322)

**S1 Text. Supplementary Note and Figures**

**Supplementary Note. Setup for additional simulaiton studies**

We conducted two additional series of simulation studies by considering pathways of 100 genes, with 20 or 40 causal genes, in contrast to the 10 causal genes described in the main text. We specified their sample sizes as the following: 4,000 from AFR, 6,000 from AMR, 6,000 from EAS, 10,000 from EUR, and 4,000 from SAS, with an equal number of cases and controls. To ensure that the powers of the considered pathway analysis procedures remained within a reasonable range, we adjusted the effect sizes of functional SNPs accordingly across all disease models.

For the pathway with 20 causal genes, in the Common Risk Model, we designated $R^{\left( l \right)}=\left\{ 1,2,\ldots, 20 \right\}$, and set $\beta^{\left( l \right)}=0.05,$ for $l=1,\ldots,5$. In Distinct Risk Models 1 and 2, the effect sizes were adjusted to $\beta^{\left( l \right)}$= -0.07, -0.05, -0.05, 0.05, and 0.07 for $l=1,\ldots,5$, respectively. In Distinct Risk Model 3, causal gene sets were defined as $R^{\left( 1 \right)}=\left\{ 1,2,\ldots, 20 \right\}$, $R^{\left( 2 \right)}=\left\{ 6,7,\ldots, 25 \right\}$, $R^{\left( 3 \right)}=\left\{ 11,12,\ldots, 30 \right\}$, $R^{\left( 4 \right)}=\left\{ 16,17,\ldots, 35 \right\}$, and $R^{\left( 5 \right)}=\left\{ 21,22,\ldots, 40 \right\}$, with different functional SNPs within shared causal genes, following the same configuration as in Distinct Risk Model 1.

Similarly, for the pathway with 40 causal genes, we adjusted the effect size to 75% of those used in the pathway with 20 causal genes. In the Common Risk Model, we designated $R^{\left( l \right)}=\left\{ 1,2,\ldots, 40 \right\}$, and set $\beta^{\left( l \right)}=0.038,$ for $l=1,\ldots,5$. The effect sizes for Distinct Risk Models 1 and 2 were adjusted to $\beta^{\left( l \right)}$= -0.053, -0.038, -0.038, 0.0038, and 0.053 for $l=1,\ldots,5$, respectively. For Distinct Risk Model 3, we defined sets of causal genes as $R^{\left( 1 \right)}=\left\{ 1,2,\ldots, 40 \right\}$, $R^{\left( 2 \right)}=\left\{ 11,12,\ldots, 50 \right\}$, $R^{\left( 3 \right)}=\left\{ 21,22,\ldots, 60 \right\}$, $R^{\left( 4 \right)}=\left\{ 31,32,\ldots, 70 \right\}$, and $R^{\left( 5 \right)}=\left\{ 41,42,\ldots, 80 \right\}$. When populations shared a causal gene in this model, they had different functional SNPs within that gene, with effect sizes following the same configuration as in Distinct Risk Model 1.

**Fig A. Power Comparison in Pathway Analysis for a 100-Gene Pathway with 10 Causal Genes and a 1:10 Case-Control Ratio.** Power is estimated from 2,000 replicates at a type I error rate of 0.05. Detailed method descriptions can be found in the footnotes of Table 1 in the main text.


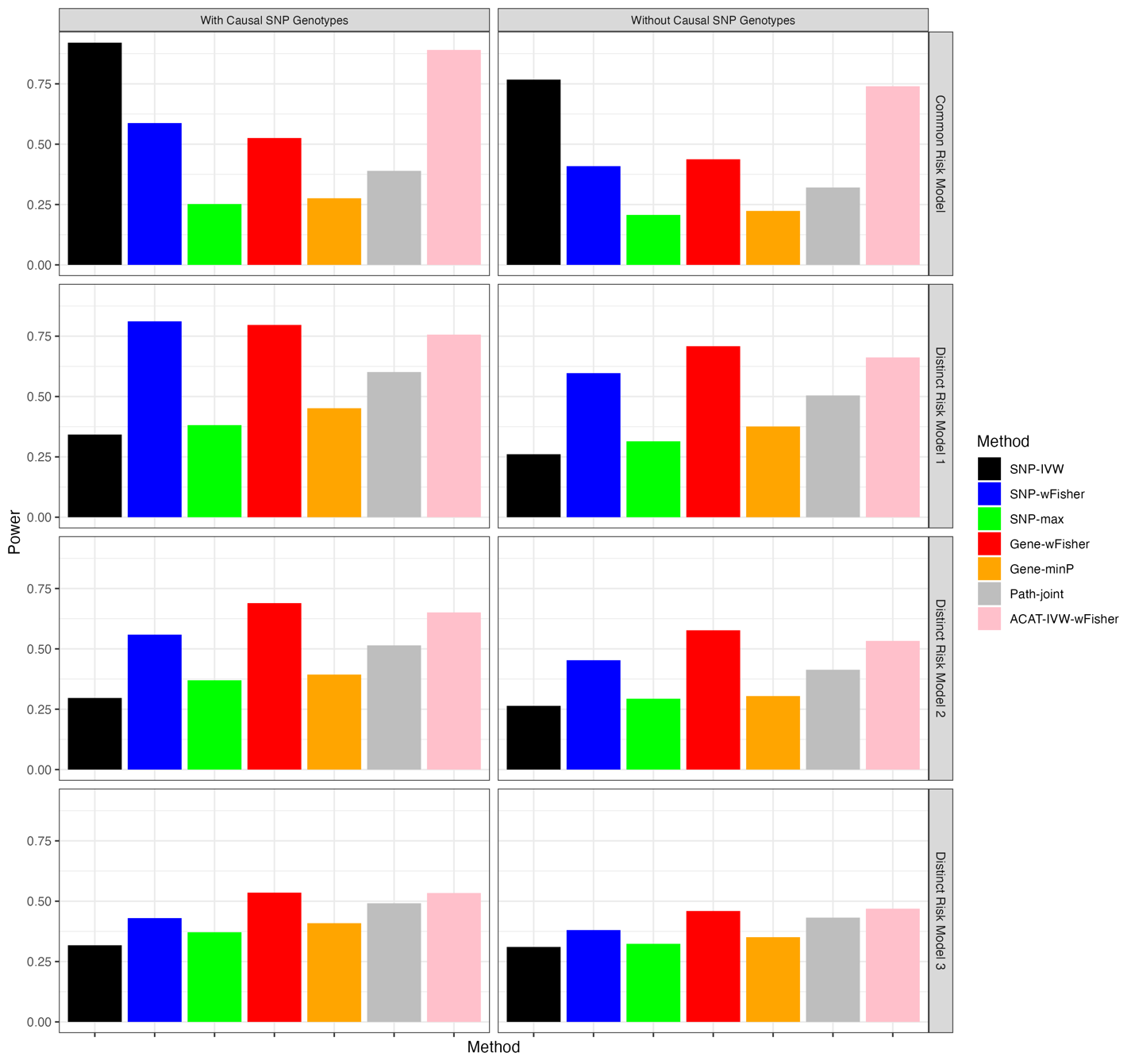


**Fig B. Power Comparison in Pathway Analysis for a 100-Gene Pathway with 20 Causal Genes and a 1:1 Case-Control Ratio.** Power is estimated from 2,000 replicates at a type I error rate of 0.05. Detailed method descriptions can be found in the footnotes of Table 1 in the main text.

**
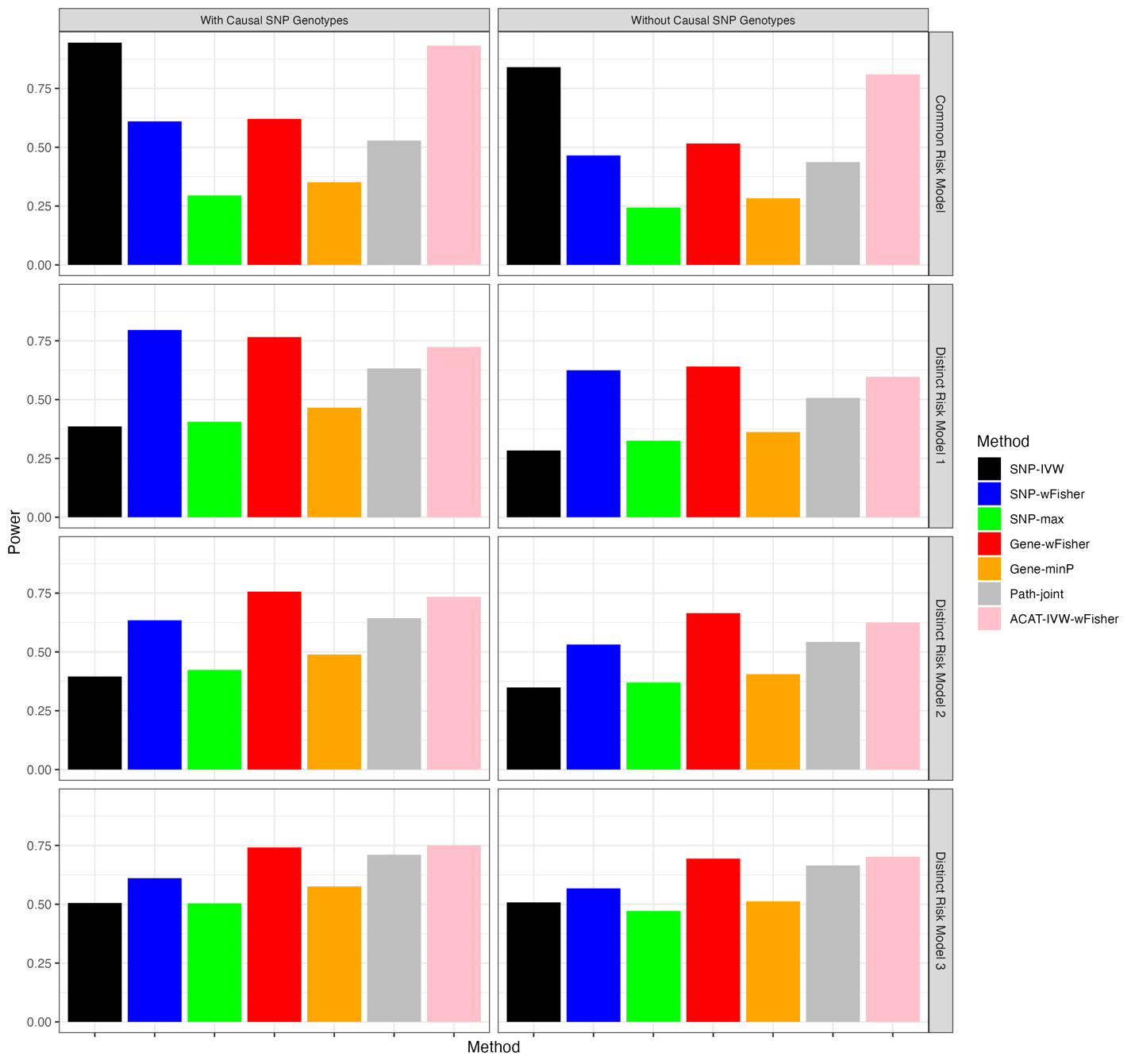
**

**Fig C. Power Comparison in Pathway Analysis for a 100-Gene Pathway with 40 Causal Genes and a 1:1 Case-Control Ratio.** Power is estimated from 2,000 replicates at a type I error rate of 0.05. Detailed method descriptions can be found in the footnotes of Table 1 in the main text.

**
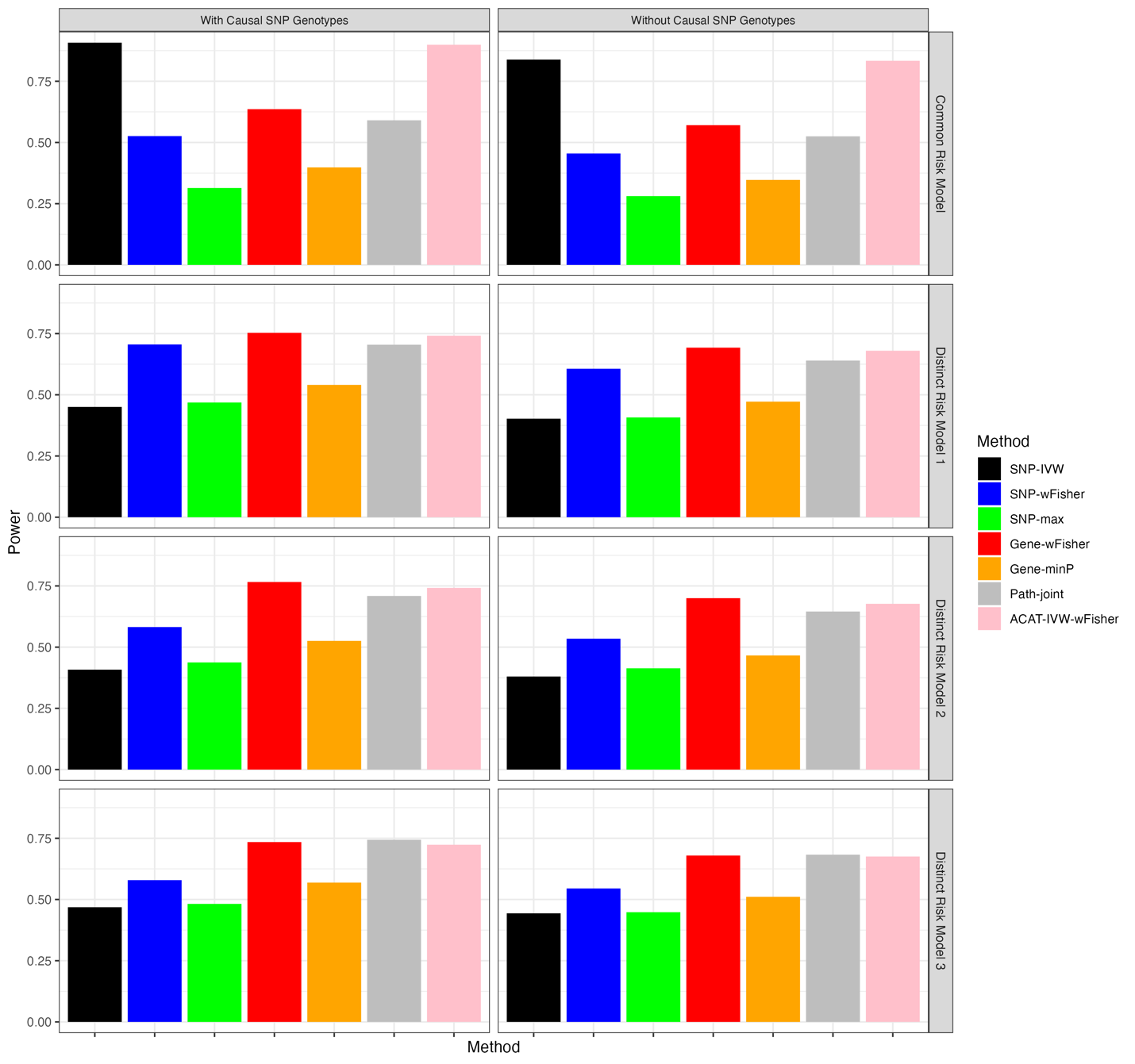
**

**Fig D. Pathway Analysis Results for the Association Between the REACTOME Antigen Processing Ubiquitination Proteasome Degradation Pathway and Schizophrenia**. Each panel shows the gene-level p-values based on different pathway analysis procedures. Path-AFR, Path-EAS, and Path-EUR refer to SA-pathway analyses of African, East Asian, and European GWAS, respectively. Gene-wFisher represents the gene-centric TA-pathway analysis based on the weighted Fisher's method, while SNP-IVW denotes the SNP-centric TA-pathway analysis based on the inverse variance weighting method. The pathway association p-value is indicated beneath the name of each method. The y-axis represents the gene-level p-value in a $-\log_{10}$ scale, and the x-axis represents the chromosomes indicating the genomic locations of the genes.

**
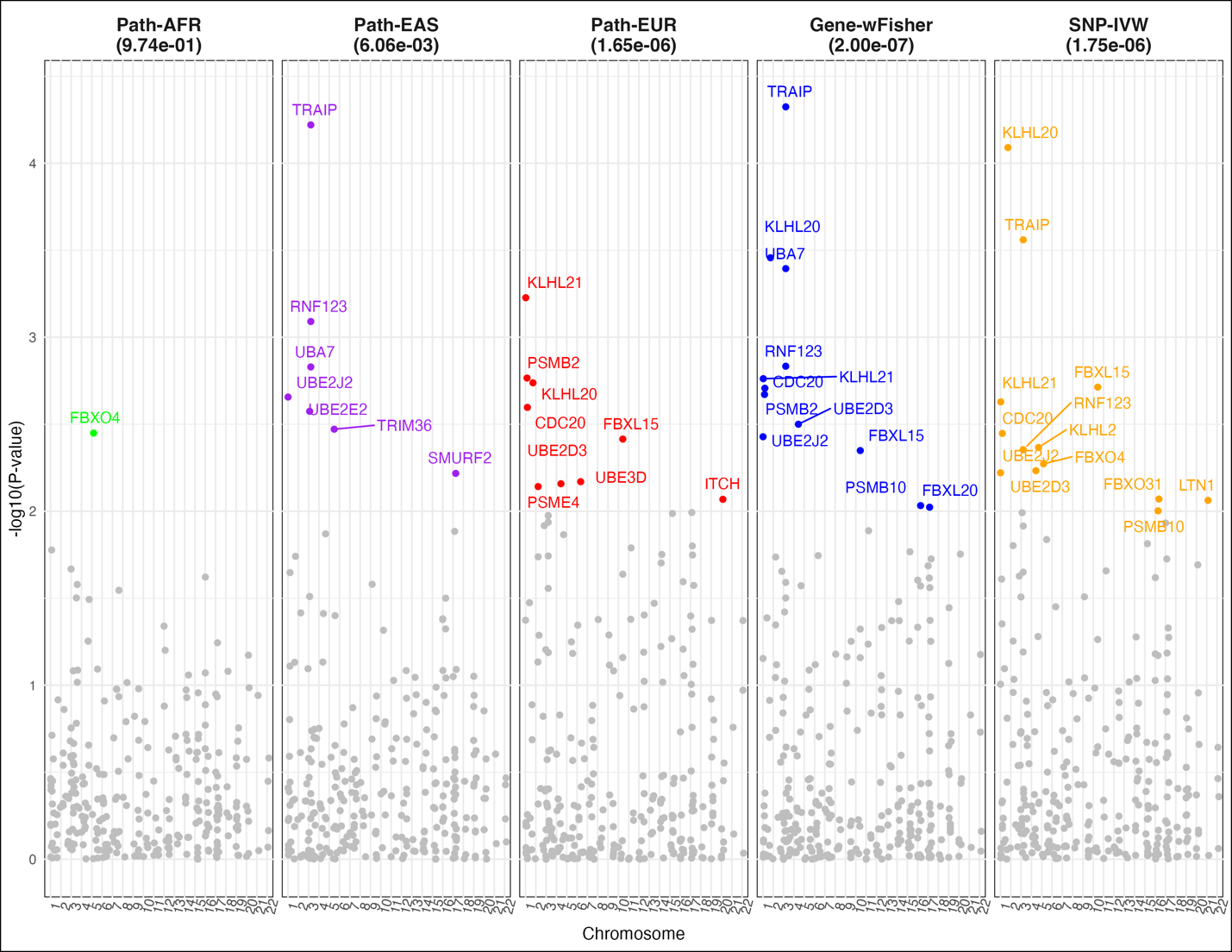
**

**Fig E. Pathway Analysis Results for the Association Between the REACTOME Arms Mediated Activation Pathway and Schizophrenia.**

**
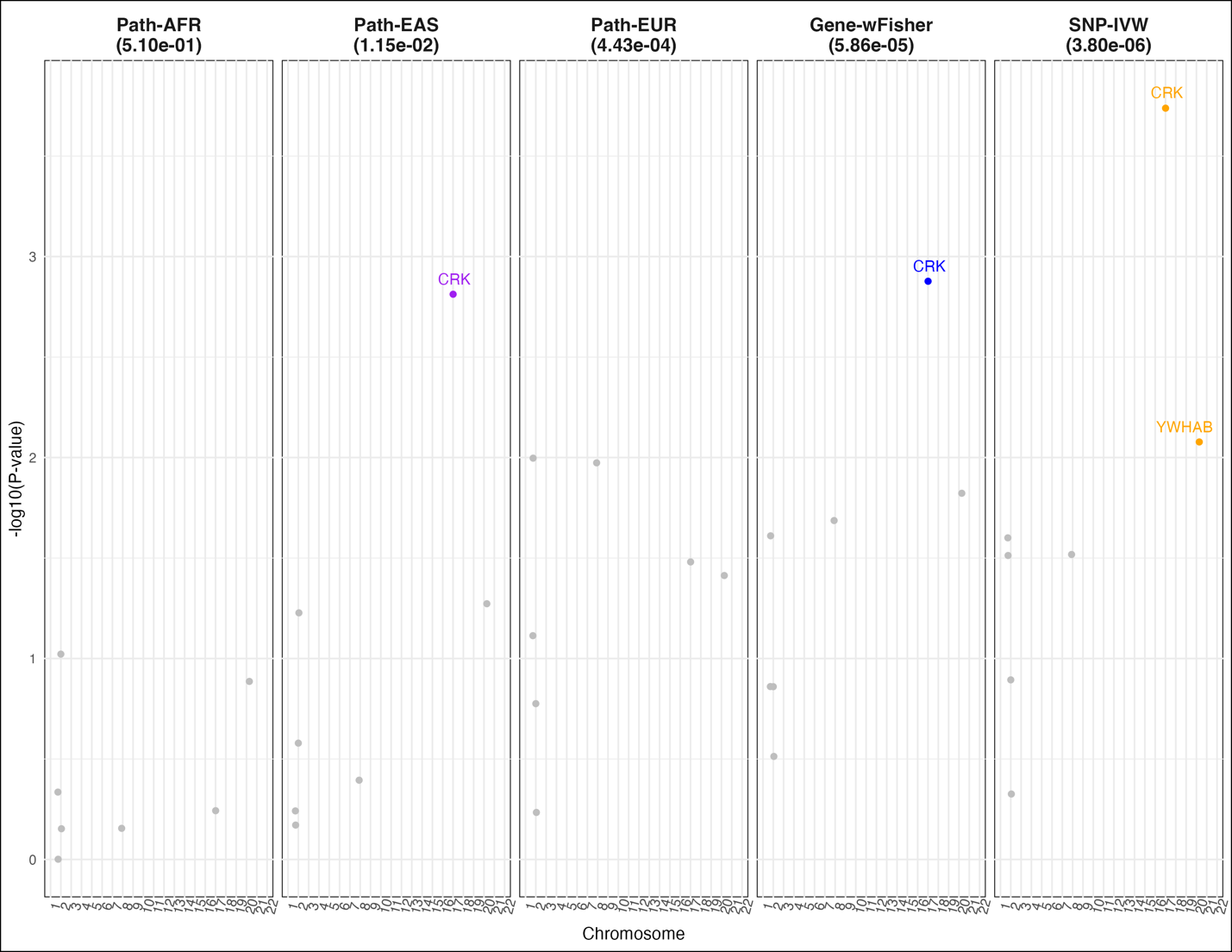
**

**Fig F. Pathway Analysis Results for the Association Between the REACTOME Asparagine N Linked Glycosylation Pathway and Schizophrenia.**

**
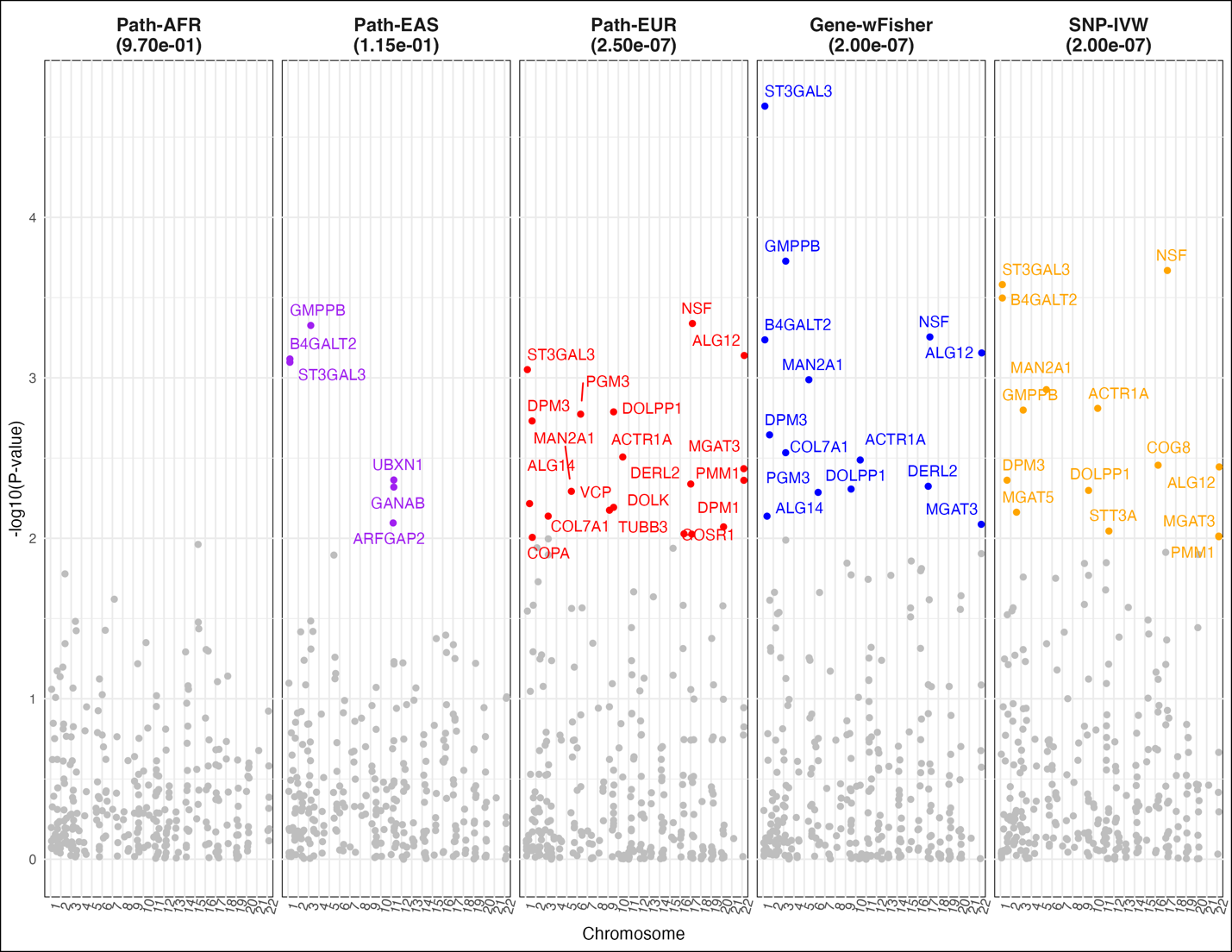
**

**Fig G. Pathway Analysis Results for the Association Between the REACTOME Biosynthesis of the N Glycan Precursor Dolichol Lipid Linked Oligosaccharide Llo and Transfer to a Nascent Protein Pathway and Schizophrenia.**

**
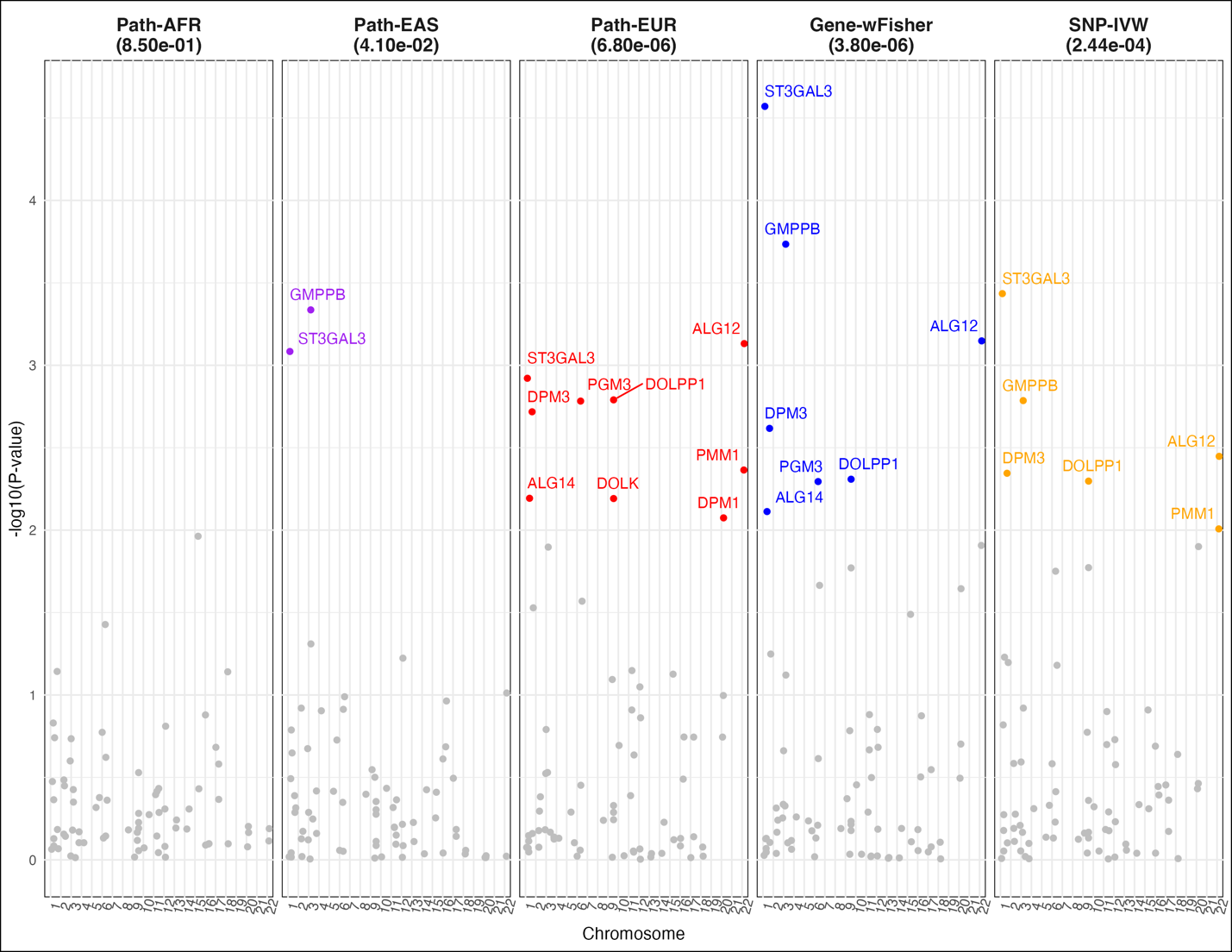
**

**Fig H. Pathway Analysis Results for the Association Between the REACTOME Cellular Response to Chemical Stress Pathway and Schizophrenia.**

**
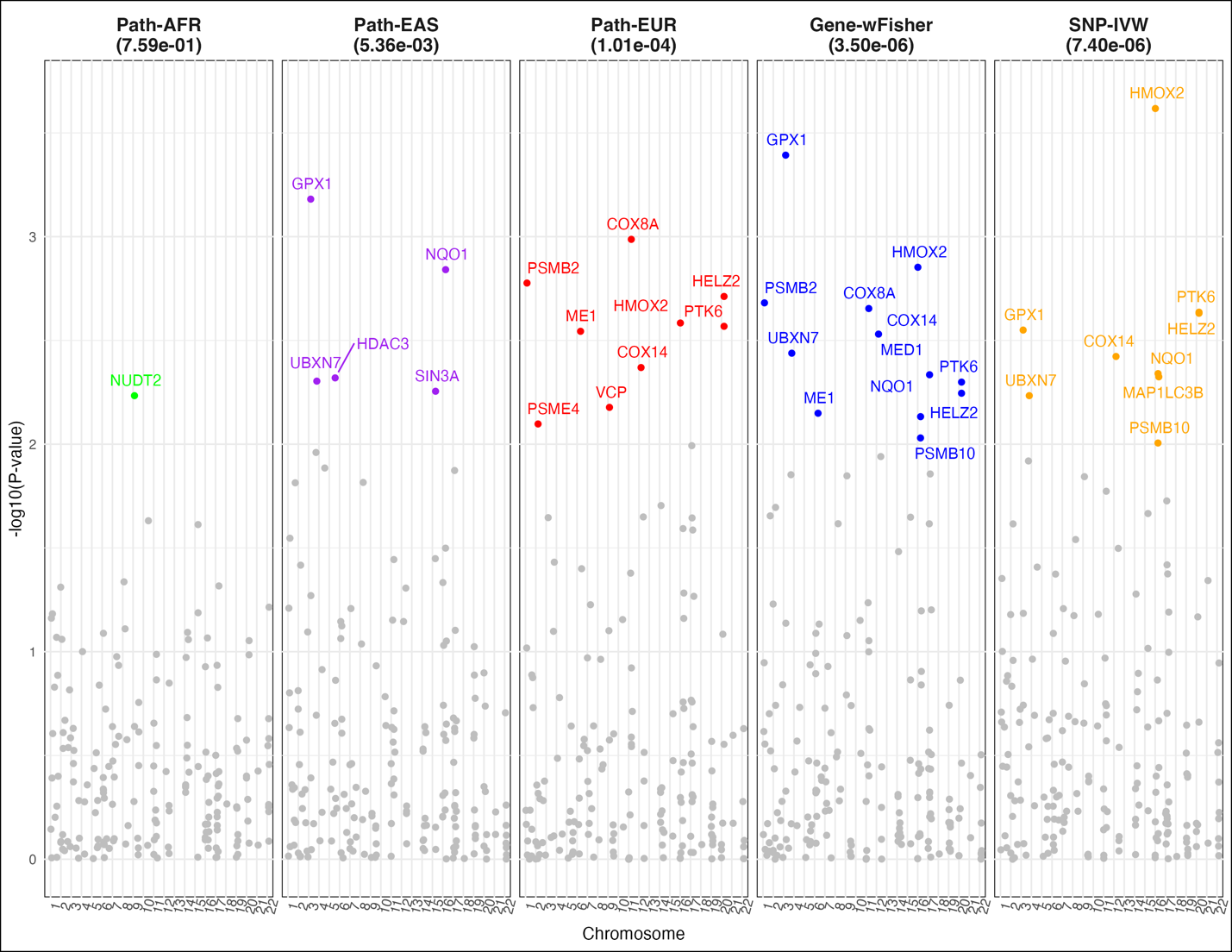
**

**Fig I. Pathway Analysis Results for the Association Between the REACTOME Cell Cycle Checkpoints Pathway and Schizophrenia.**

**
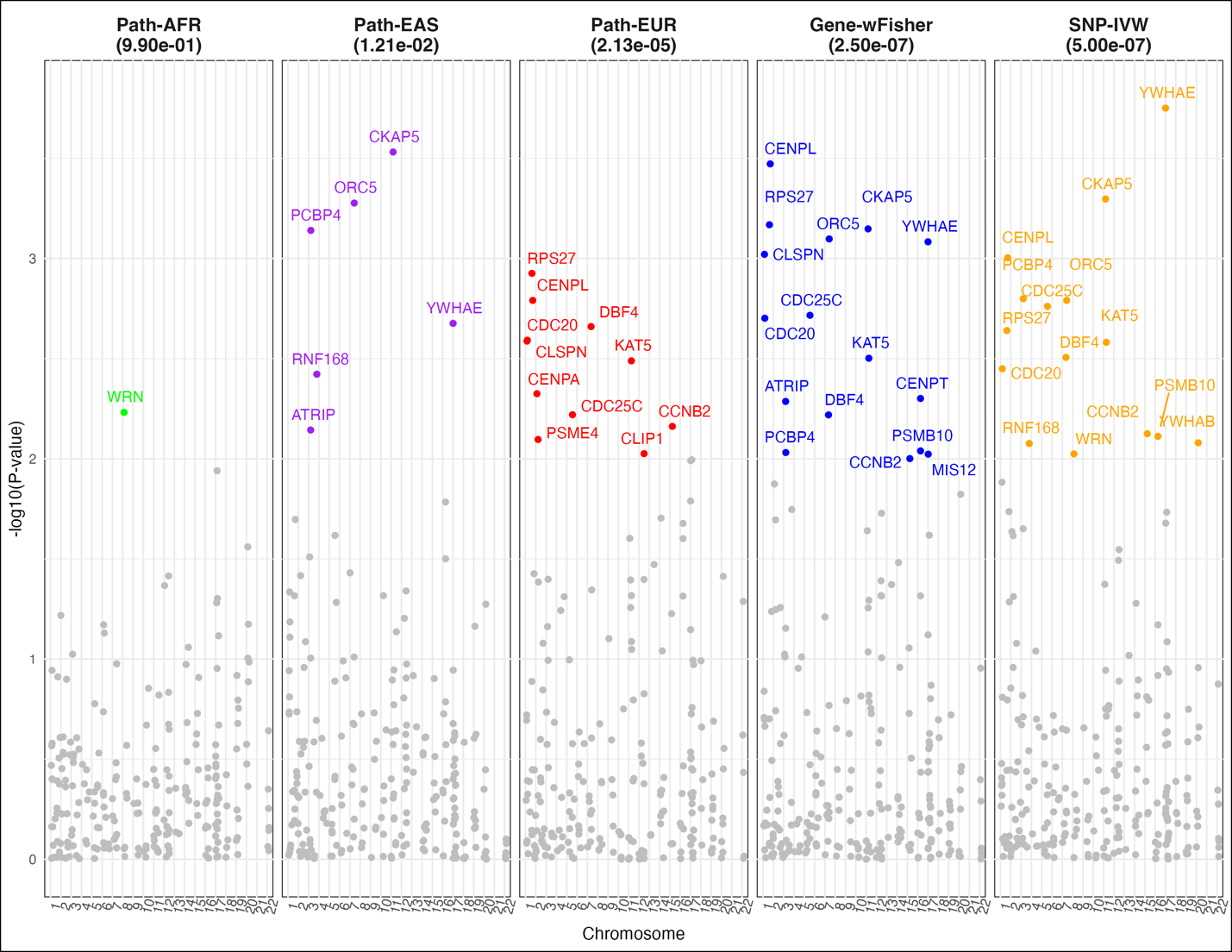
**

**Fig J. Pathway Analysis Results for the Association Between the REACTOME Cell Cycle Mitotic Pathway and Schizophrenia.**

**
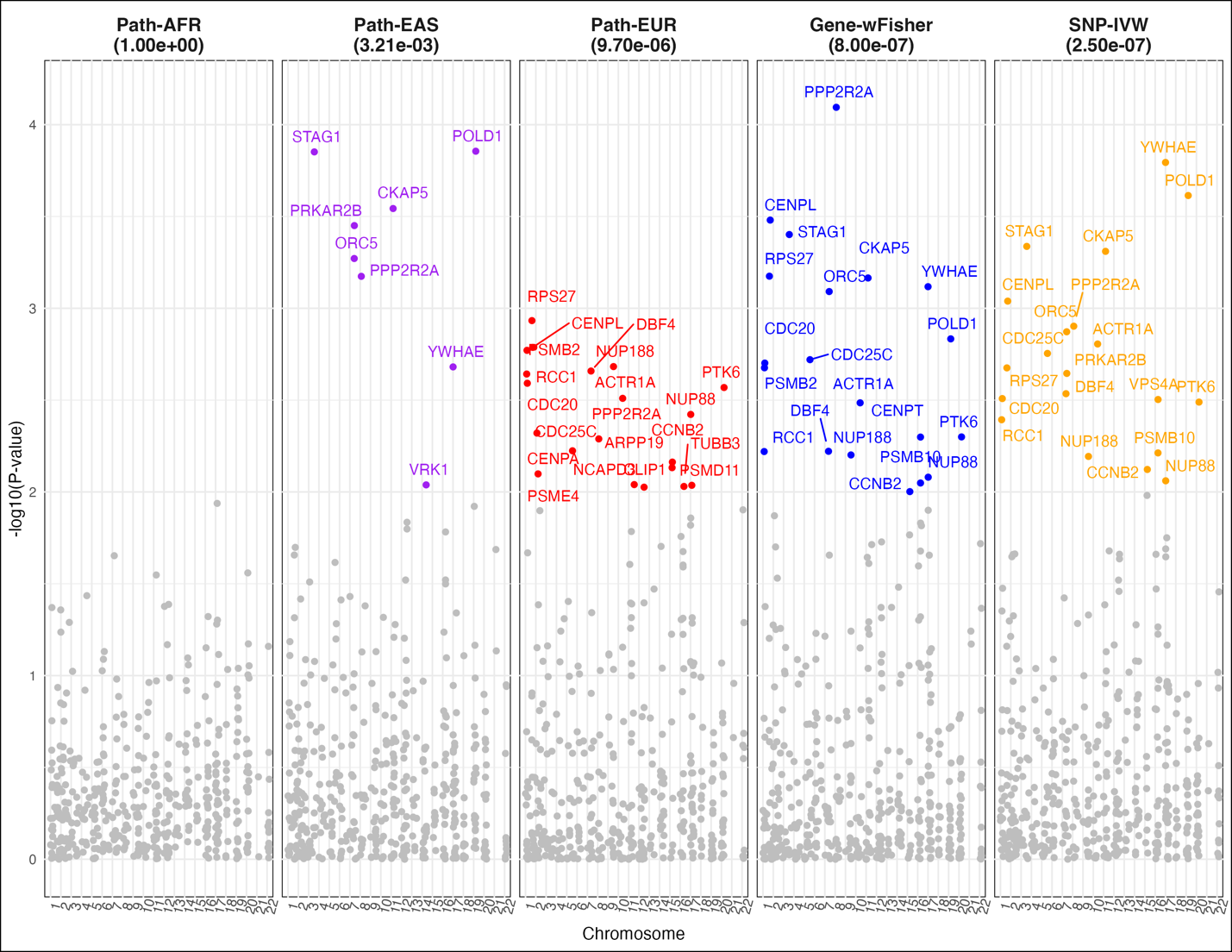
**

**Fig K. Pathway Analysis Results for the Association Between the REACTOME Chondroitin Sulfate Dermatan Sulfate Metabolism Pathway and Schizophrenia.**

**
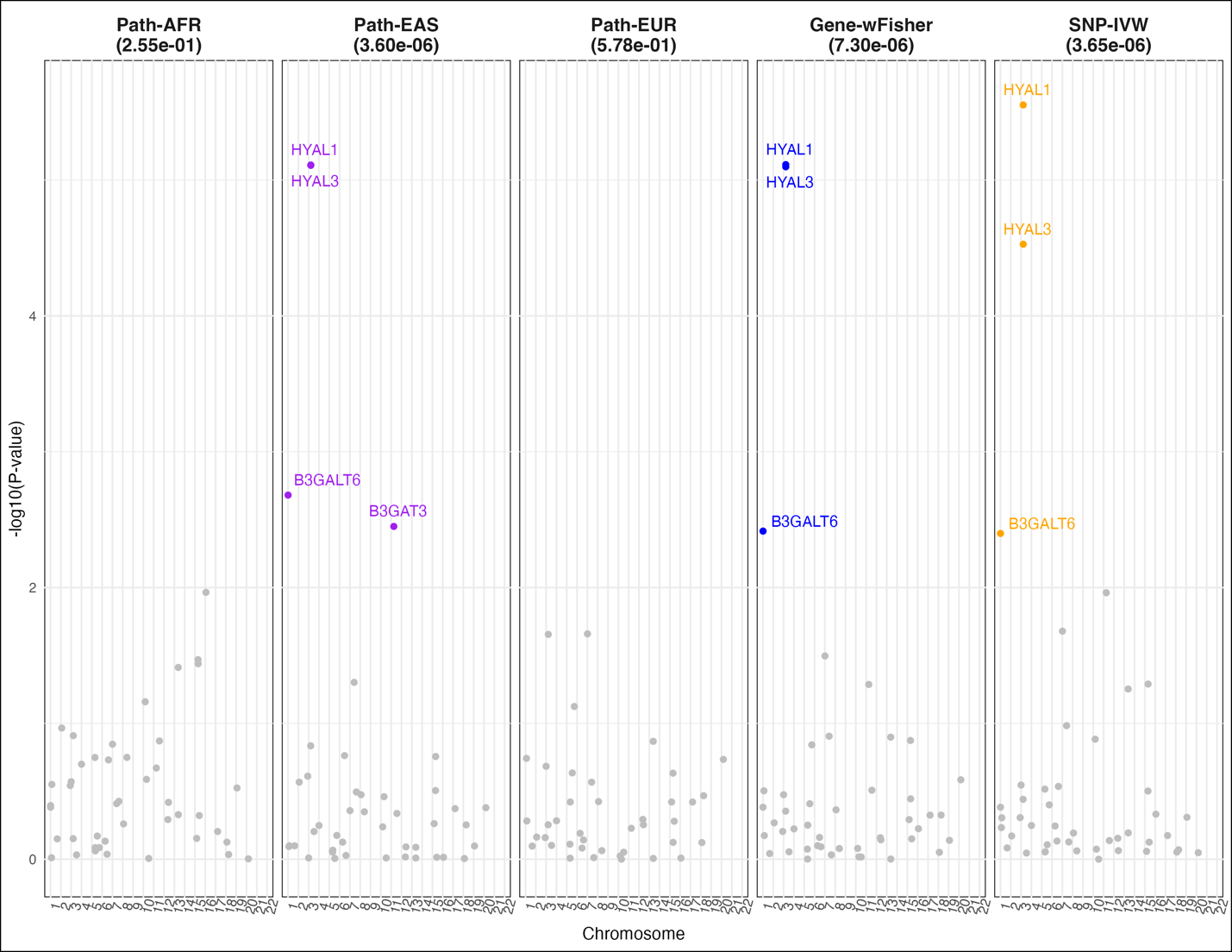
**

**Fig L. Pathway Analysis Results for the Association Between the REACTOME Chromatin Modifying Pathway and Schizophrenia.**

**
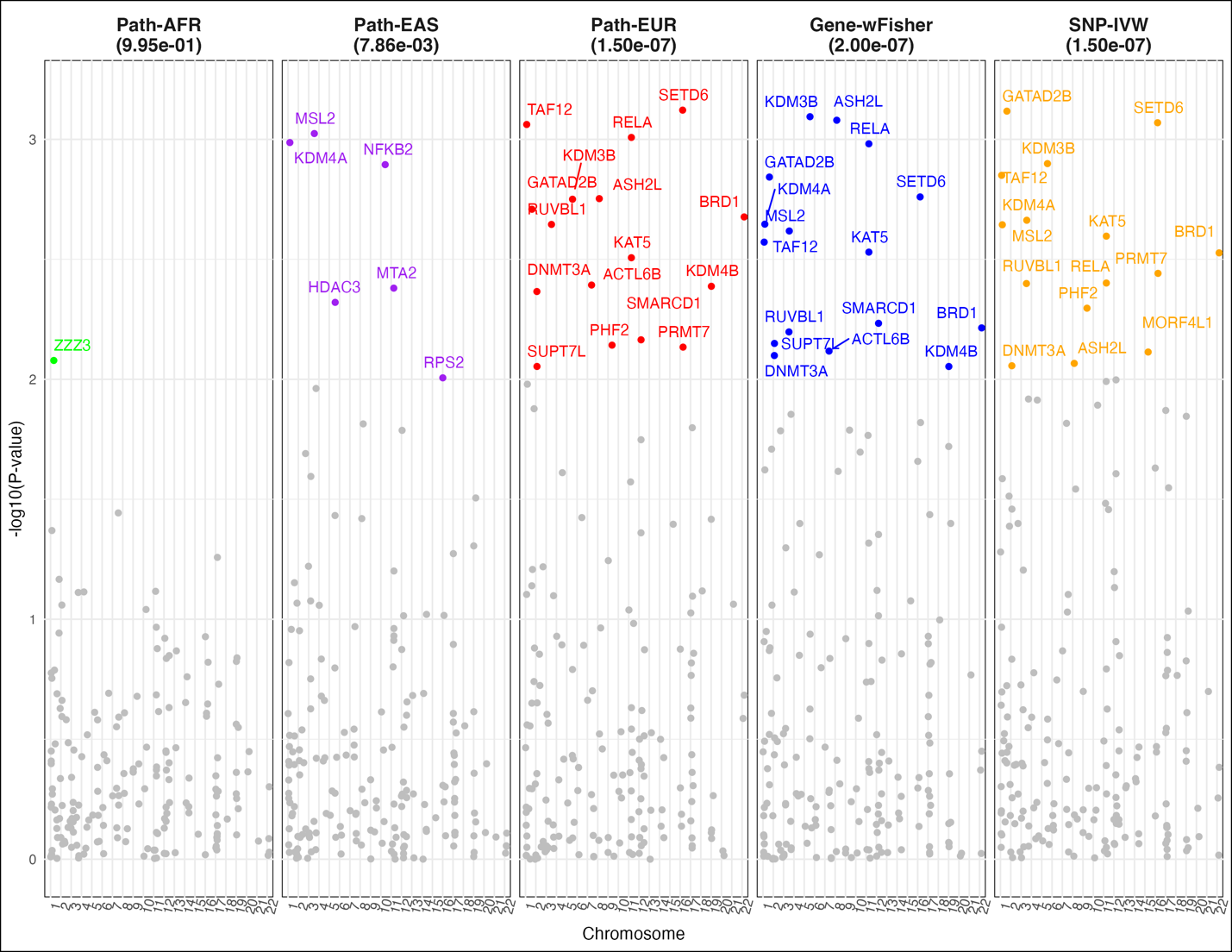
**

**Fig M. Pathway Analysis Results for the Association Between the REACTOME Class I MHC Mediated Antigen Processing Presentation Pathway and Schizophrenia.**

**
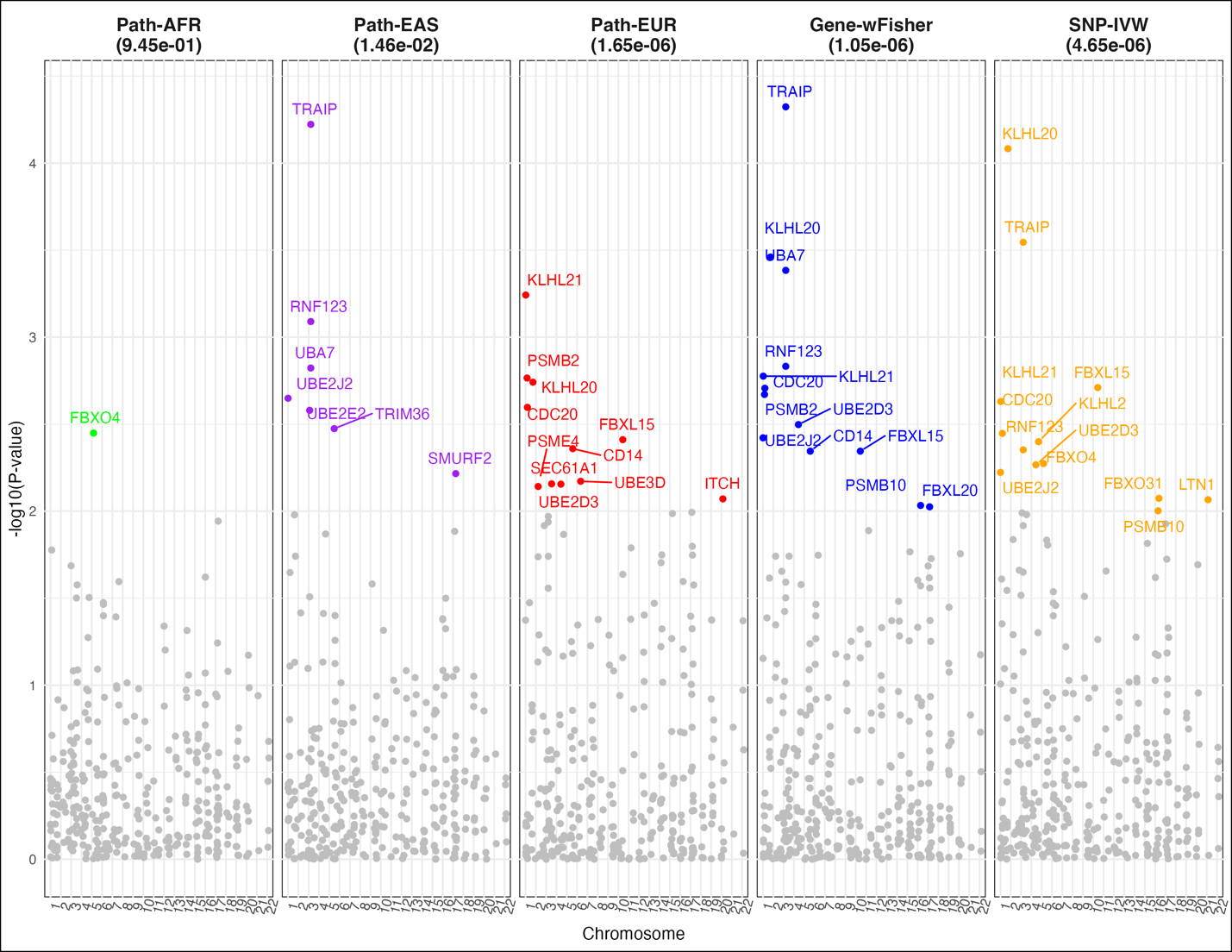
**

**Fig N. Pathway Analysis Results for the Association Between the REACTOME Cs Ds Degradation Pathway and Schizophrenia.**

**
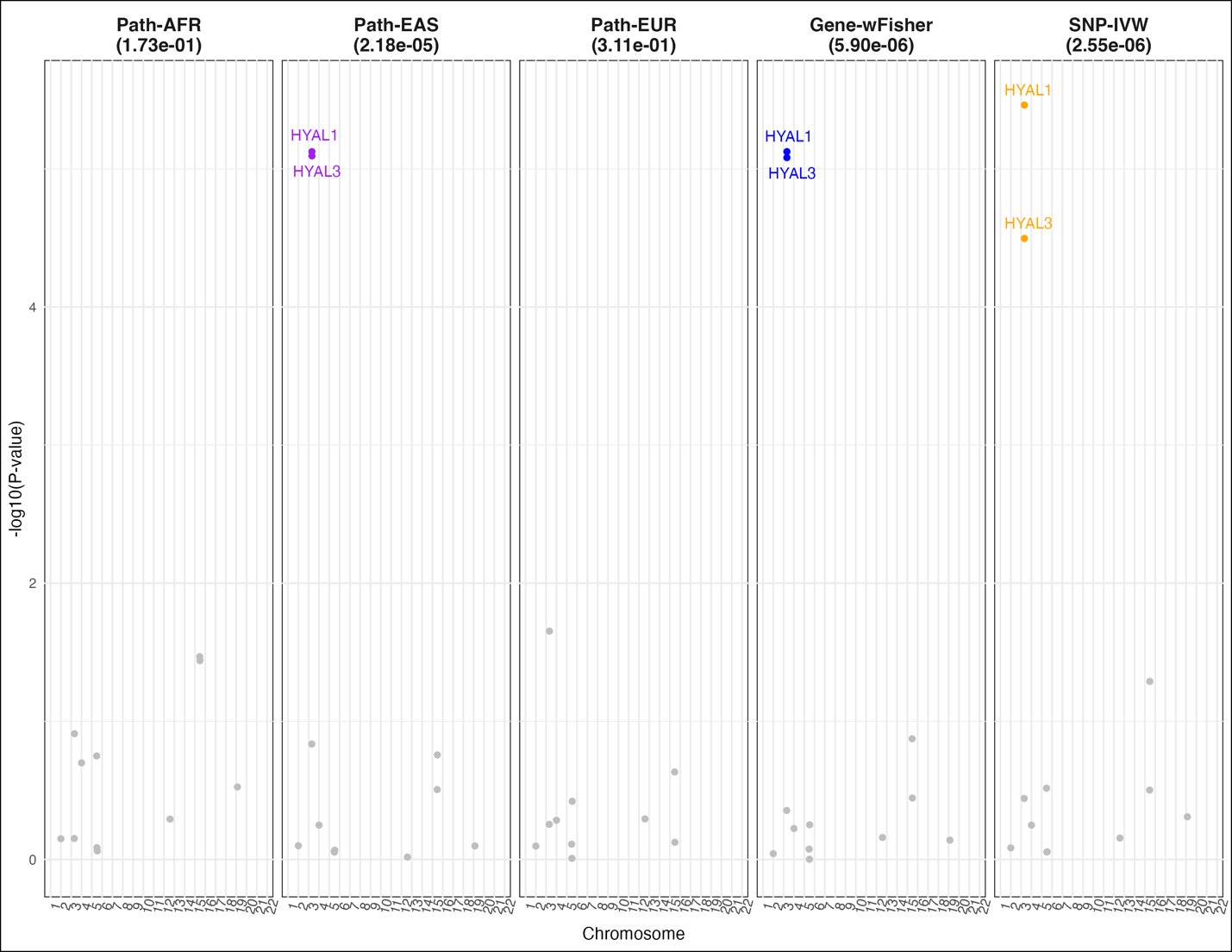
**

**Fig O. Pathway Analysis Results for the Association Between the REACTOME Deubiquitination Pathway and Schizophrenia.**

**
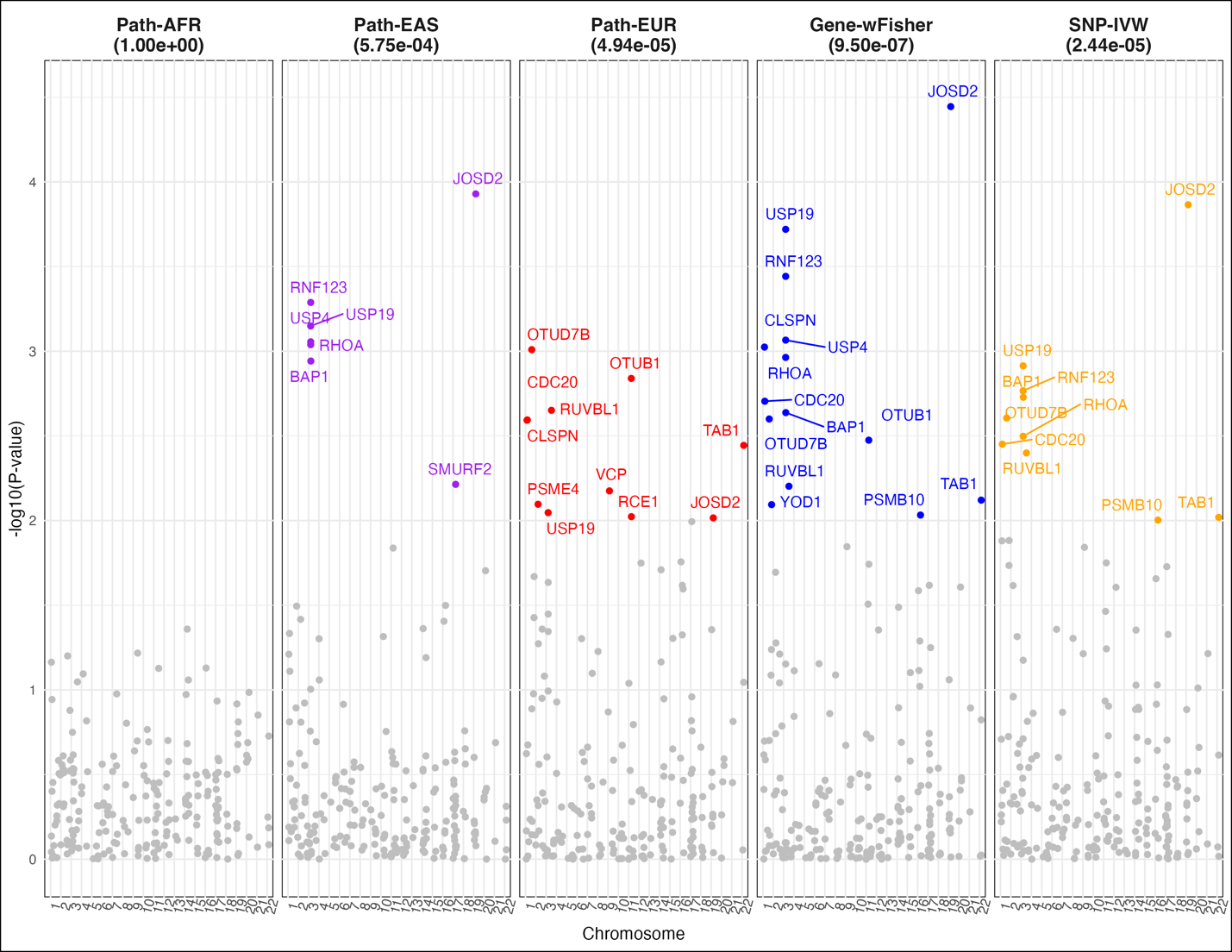
**

**Fig P. Pathway Analysis Results for the Association Between the REACTOME Diseases of Metabolism Pathway and Schizophrenia.**

**
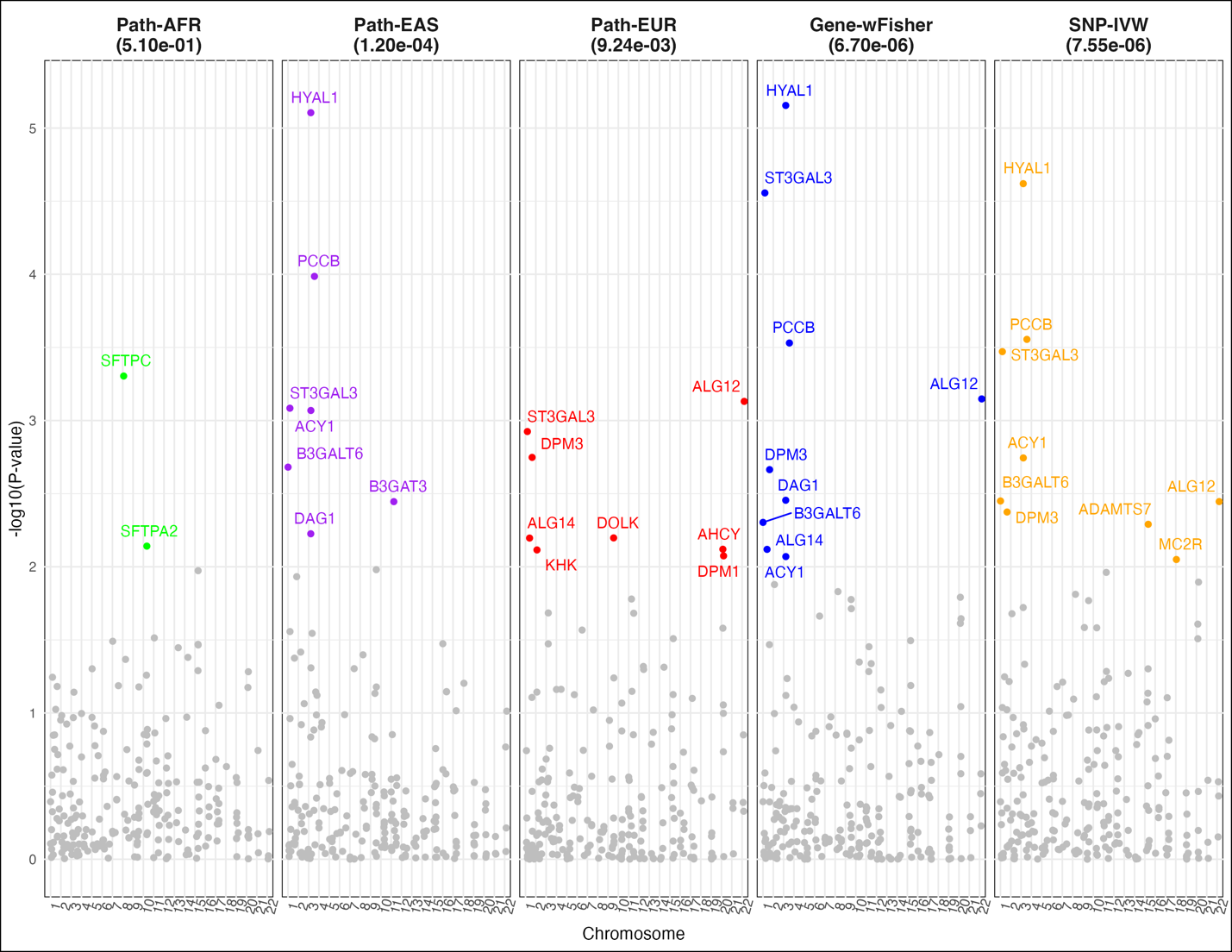
**

**Fig Q. Pathway Analysis Results for the Association Between the REACTOME Diseases of Signal Transduction by Growth Factor Receptors and Second Messengers Pathway and Schizophrenia.**

**
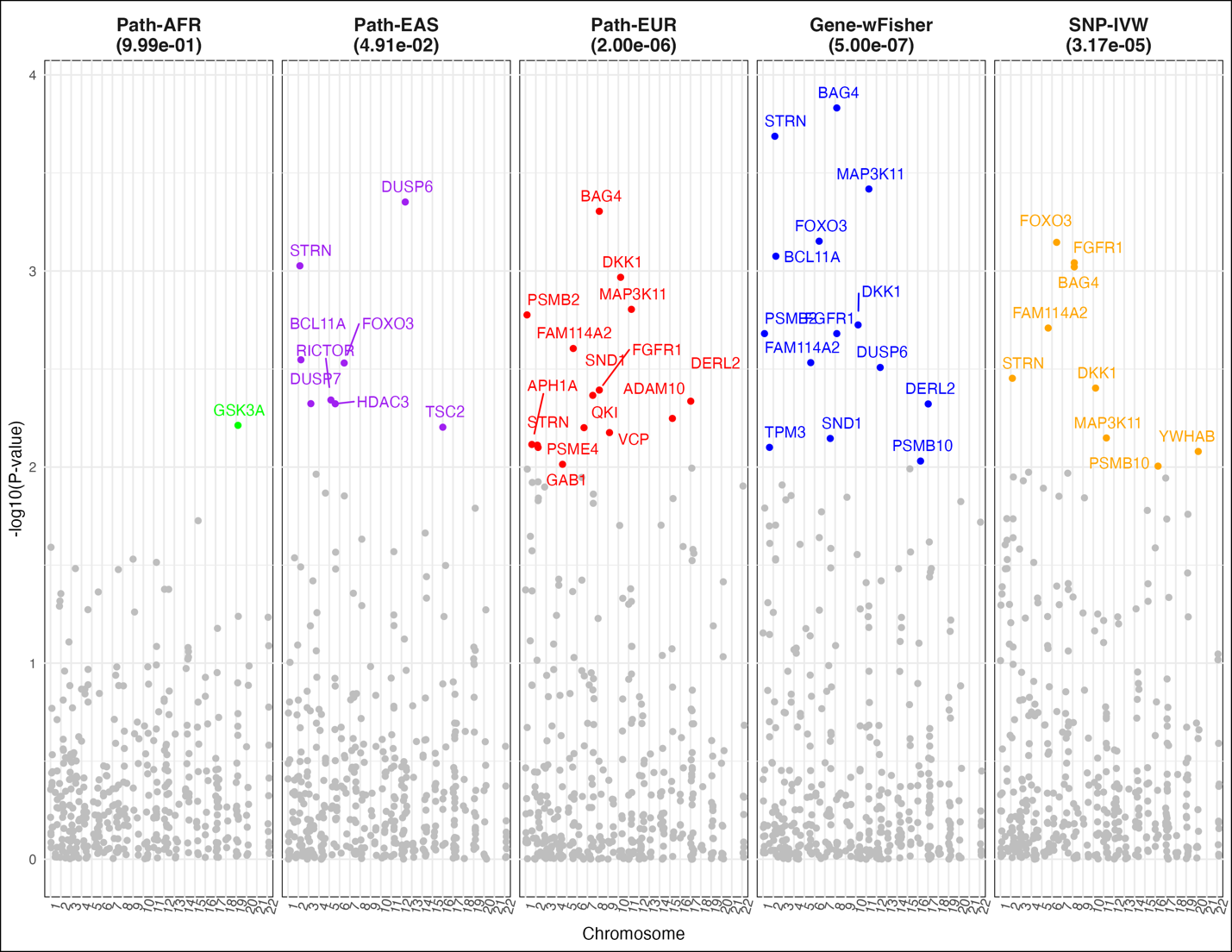
**

**Fig R. Pathway Analysis Results for the Association Between the REACTOME DNA Repair Pathway and Schizophrenia.**

**
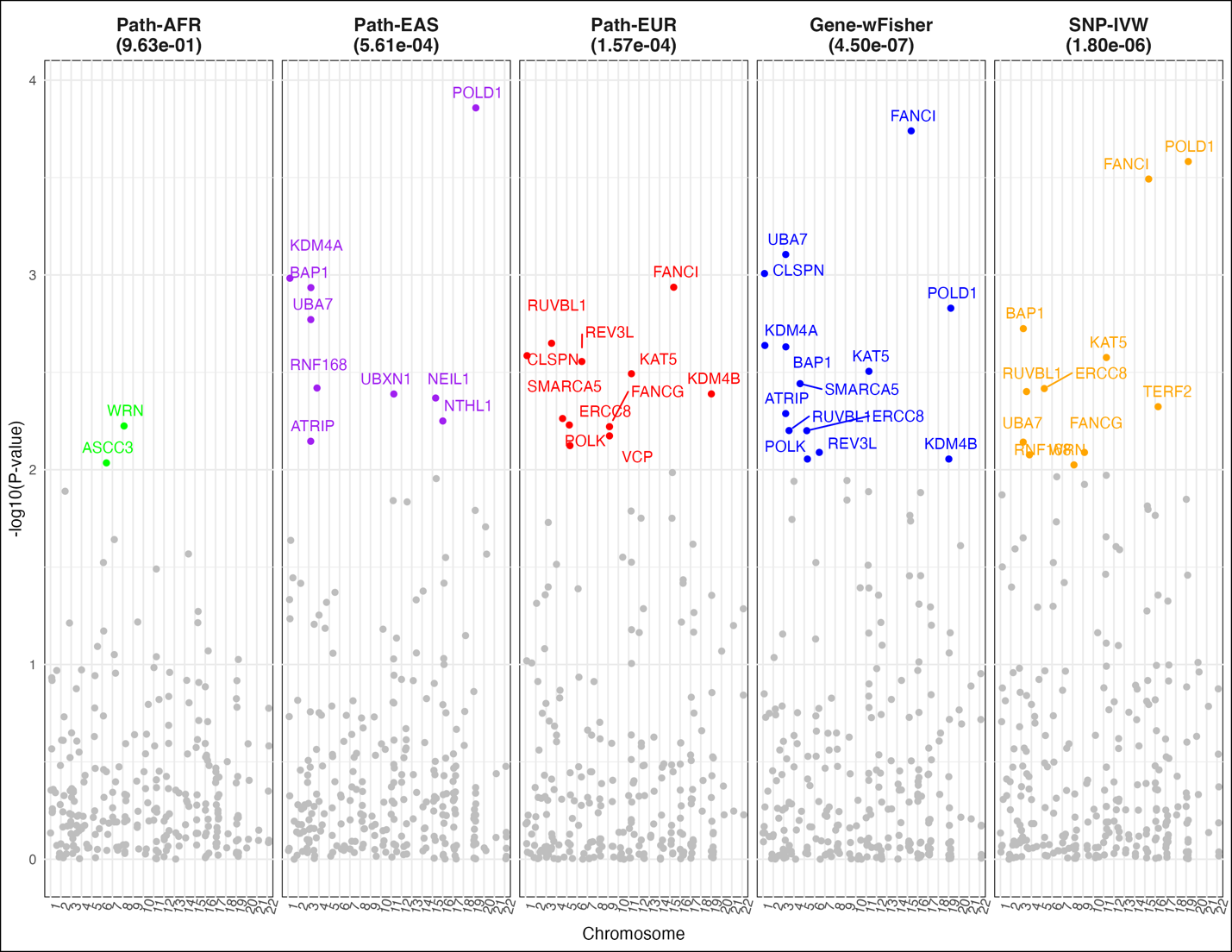
**

**Fig S. Pathway Analysis Results for the Association Between the REACTOME Esr Mediated Signaling Pathway and Schizophrenia.**

**
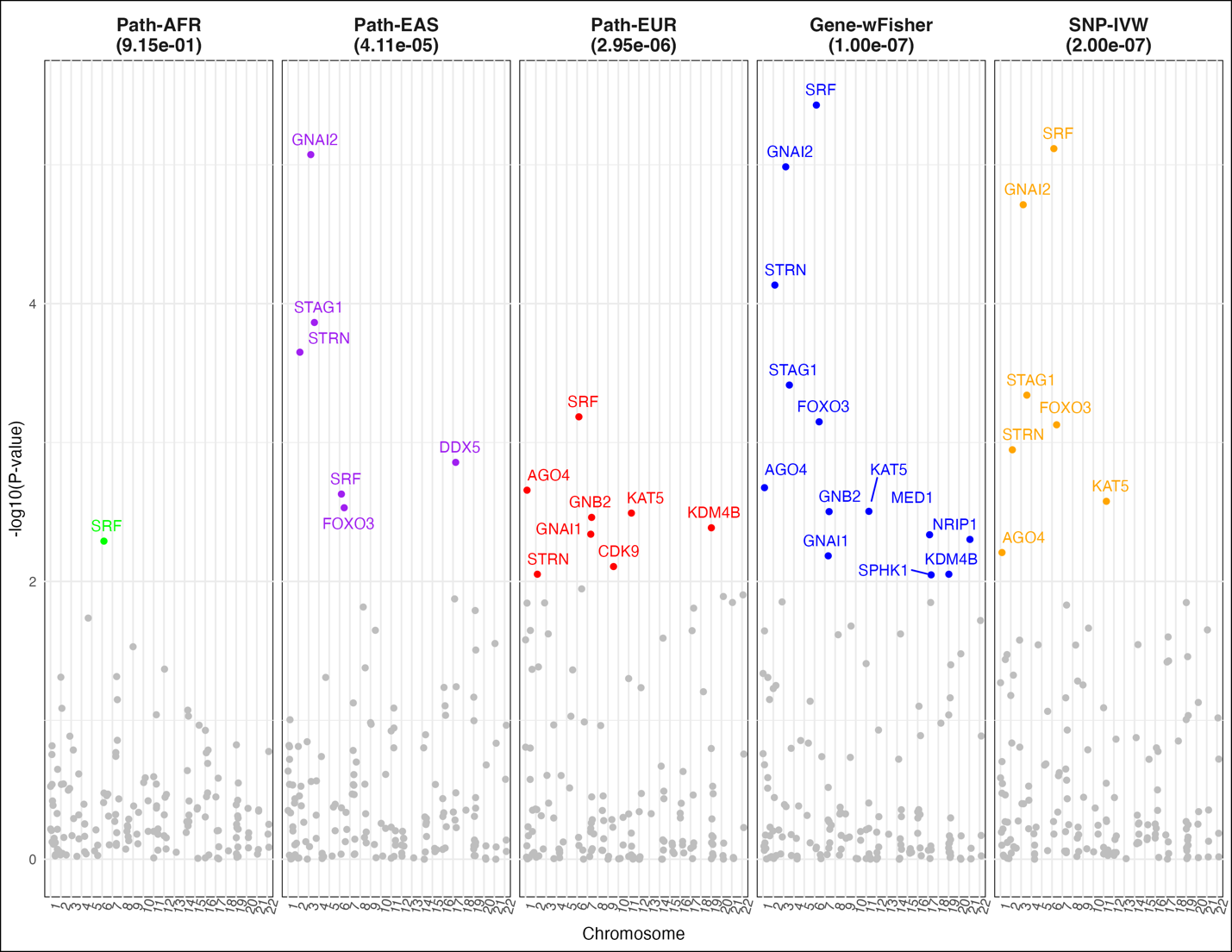
**

**Fig T. Pathway Analysis Results for the Association Between the REACTOME Extra Nuclear Estrogen Signaling Pathway and Schizophrenia.**

**
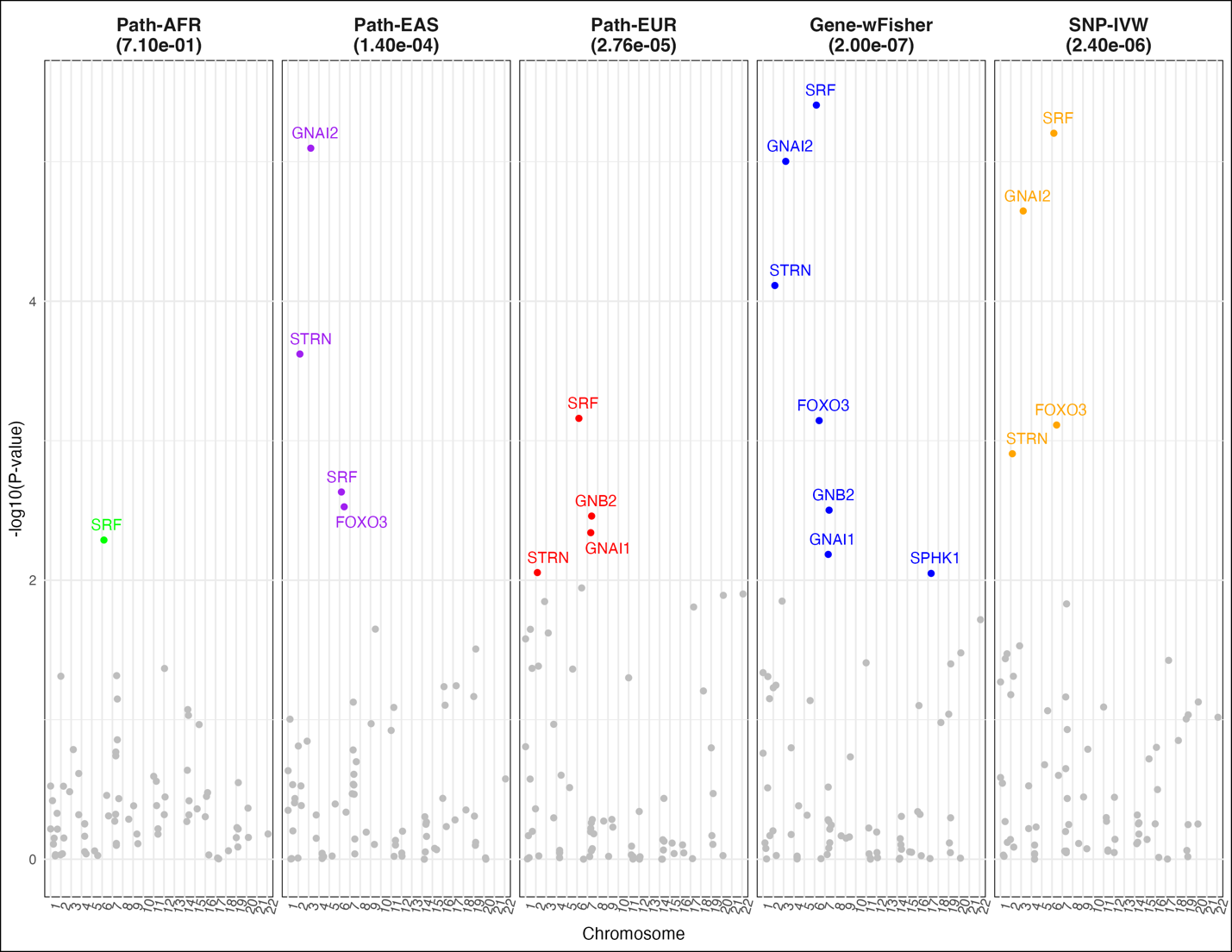
**

**Fig U. Pathway Analysis Results for the Association Between the REACTOME Glycosaminoglycan Metabolism Pathway and Schizophrenia.**

**
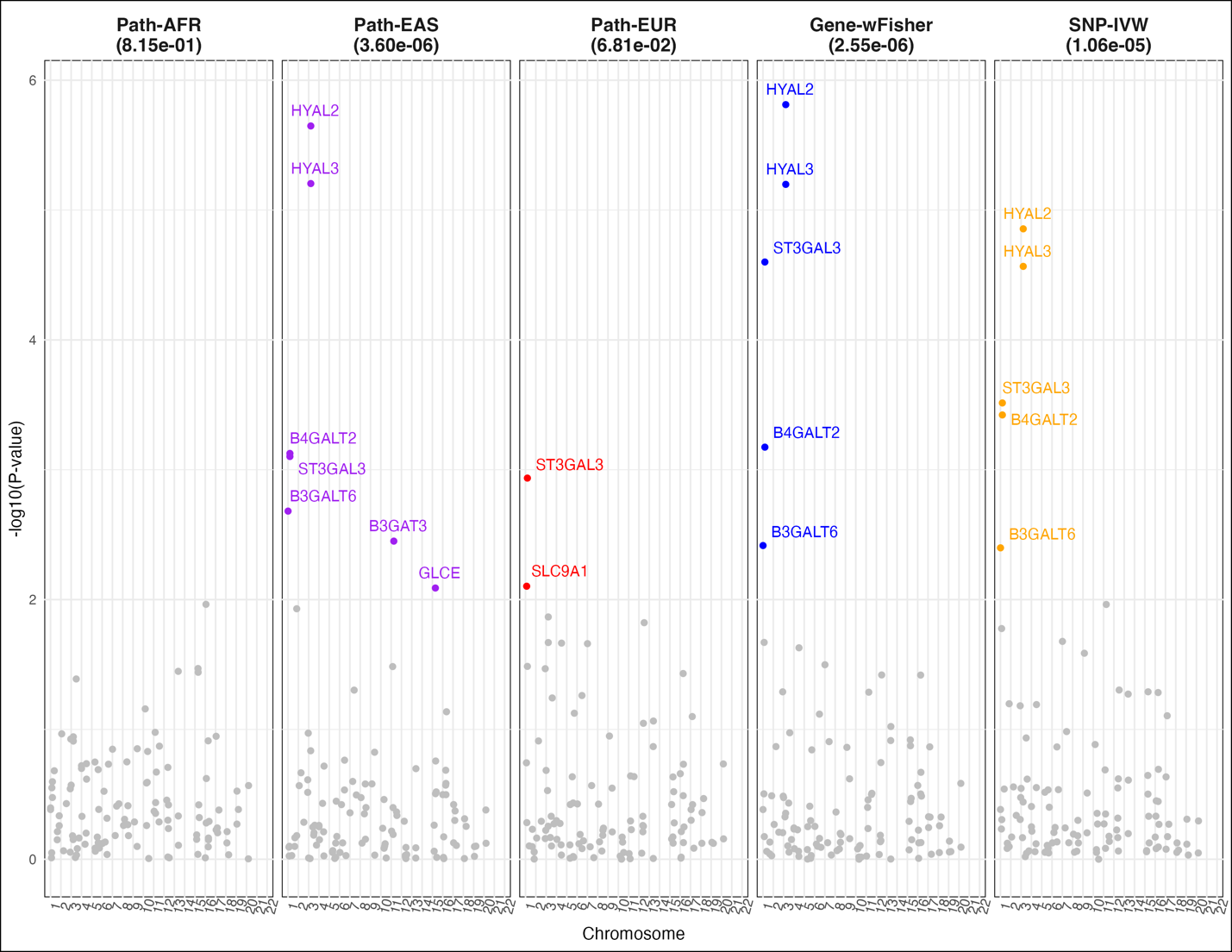
**

**Fig V. Pathway Analysis Results for the Association Between the REACTOME Leishmania Infection Pathway and Schizophrenia.**

**
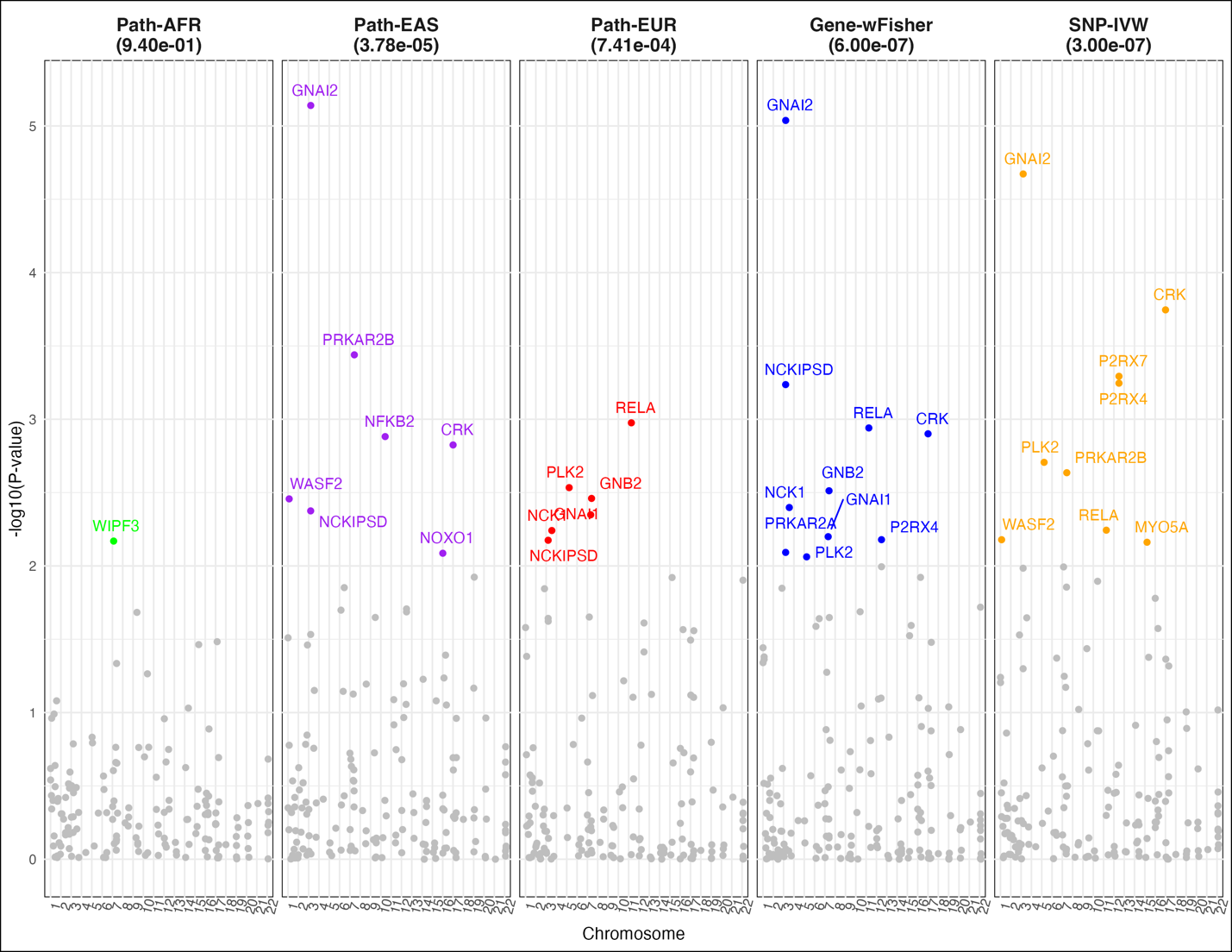
**

**Fig W. Pathway Analysis Results for the Association Between the REACTOME Metabolism of Carbohydrates Pathway and Schizophrenia.**

**
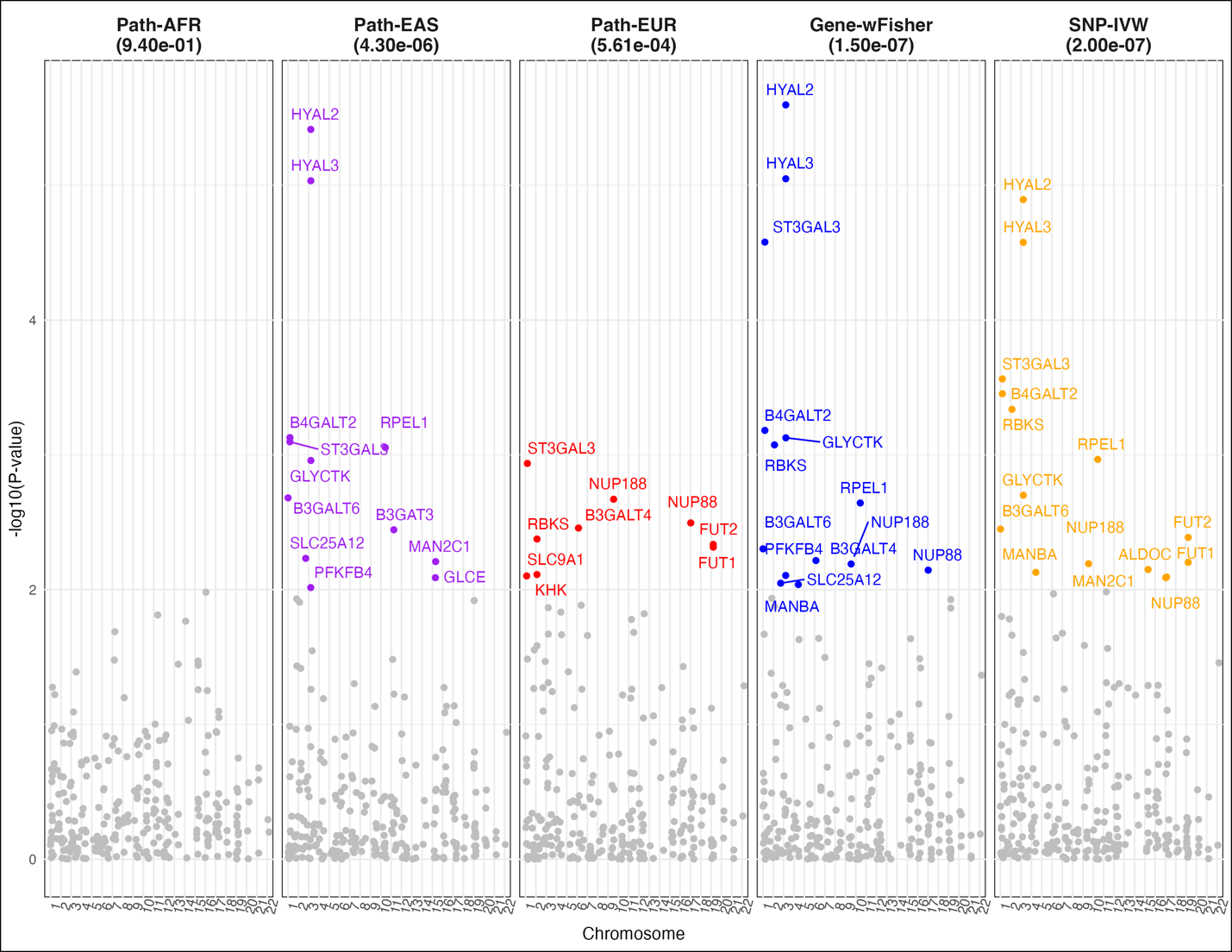
**

**Fig X. Pathway Analysis Results for the Association Between the REACTOME Mitotic Metaphase and Anaphase Pathway and Schizophrenia.**

**
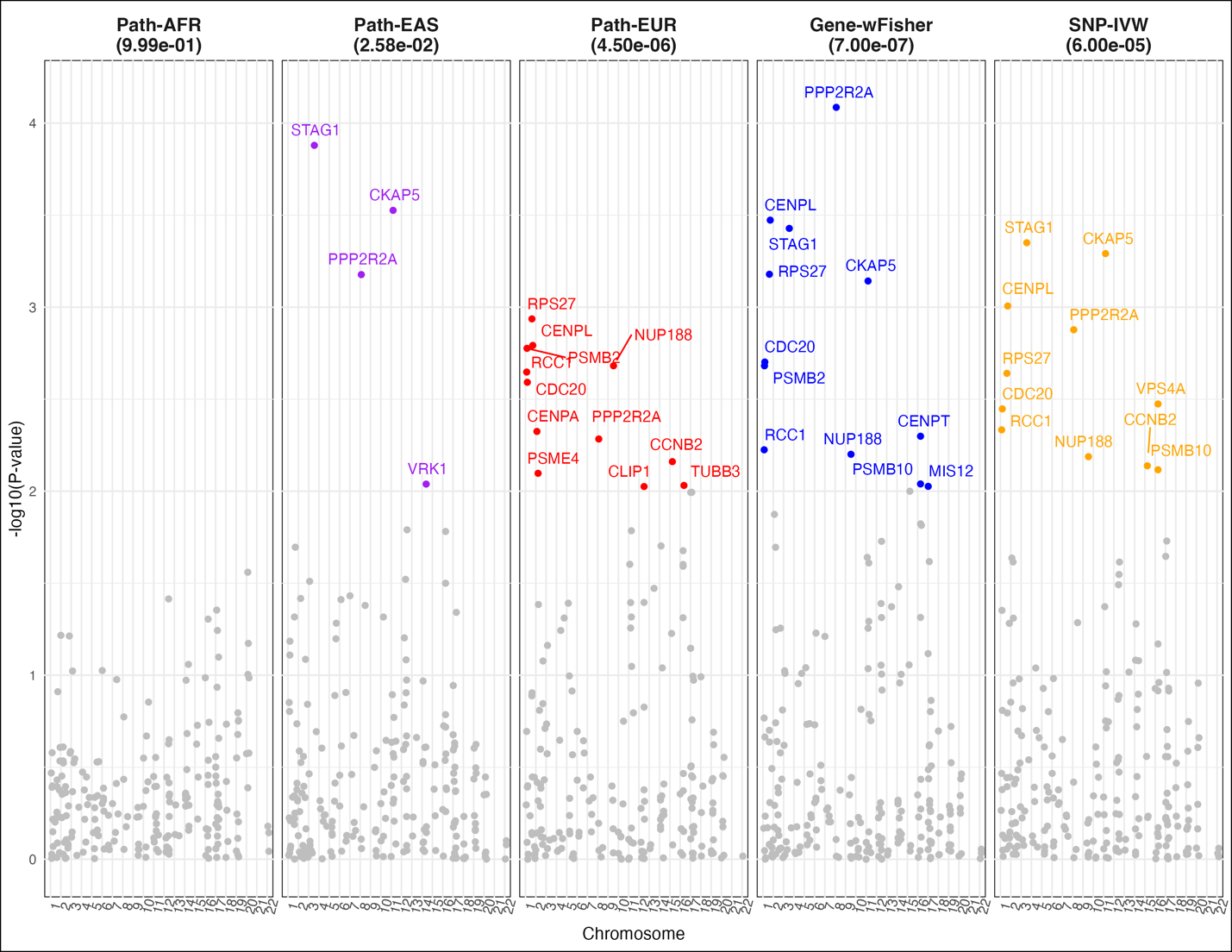
**

**Fig Y. Pathway Analysis Results for the Association Between the REACTOME Mitotic Prometaphase Pathway and Schizophrenia.**

**
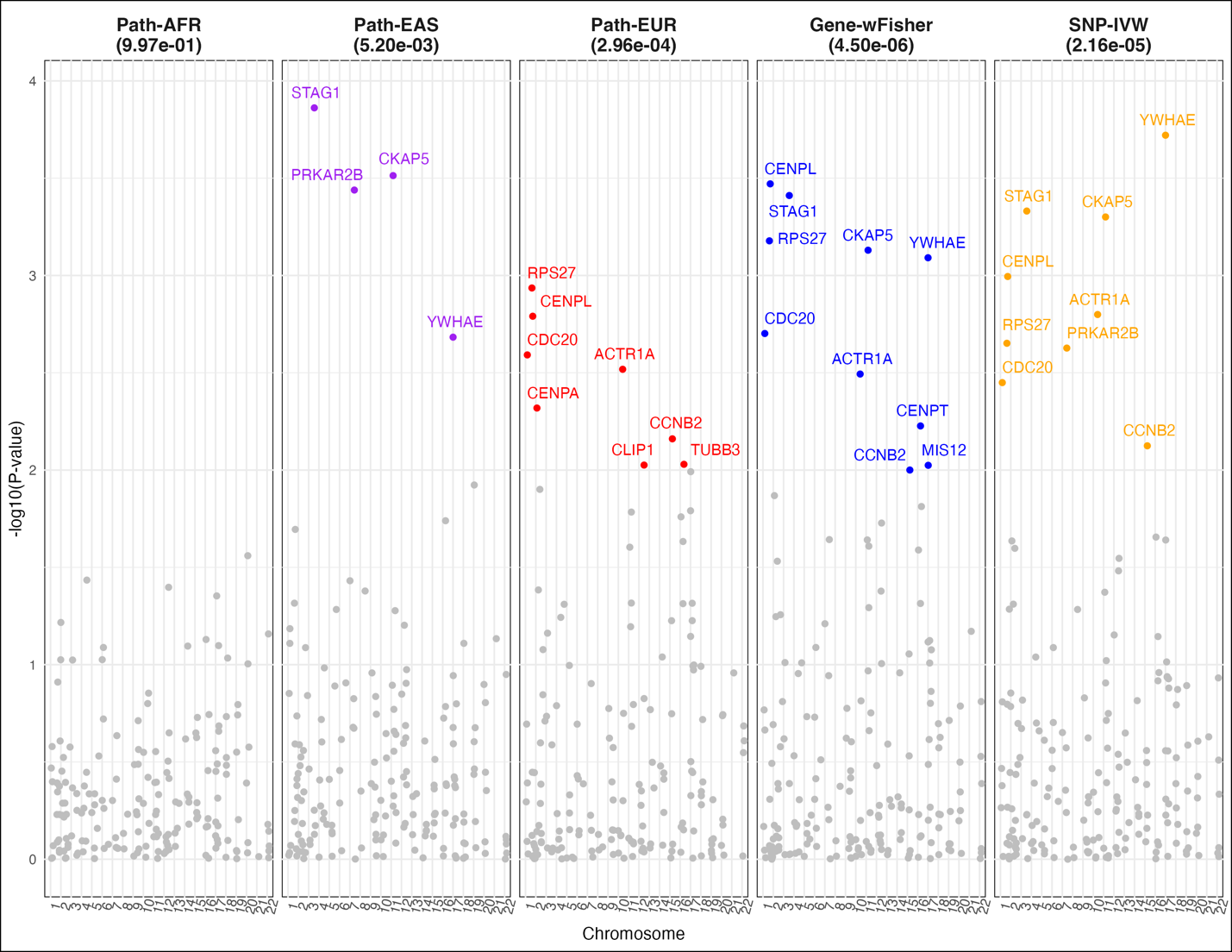
**

**Fig Z. Pathway Analysis Results for the Association Between the REACTOME M Phase Pathway and Schizophrenia.**

**
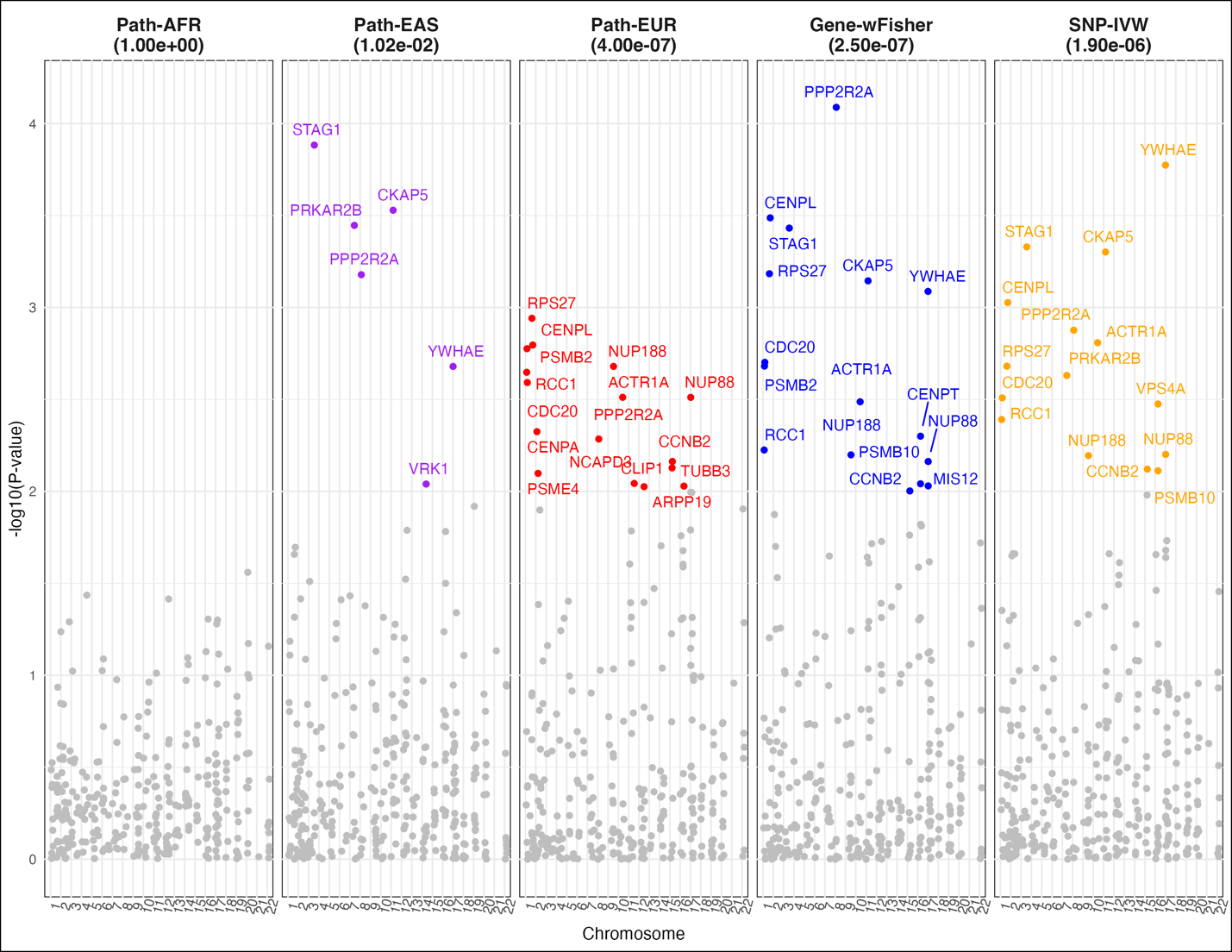
**

**Fig AA. Pathway Analysis Results for the Association Between the REACTOME Neuronal System Pathway and Schizophrenia.**

**
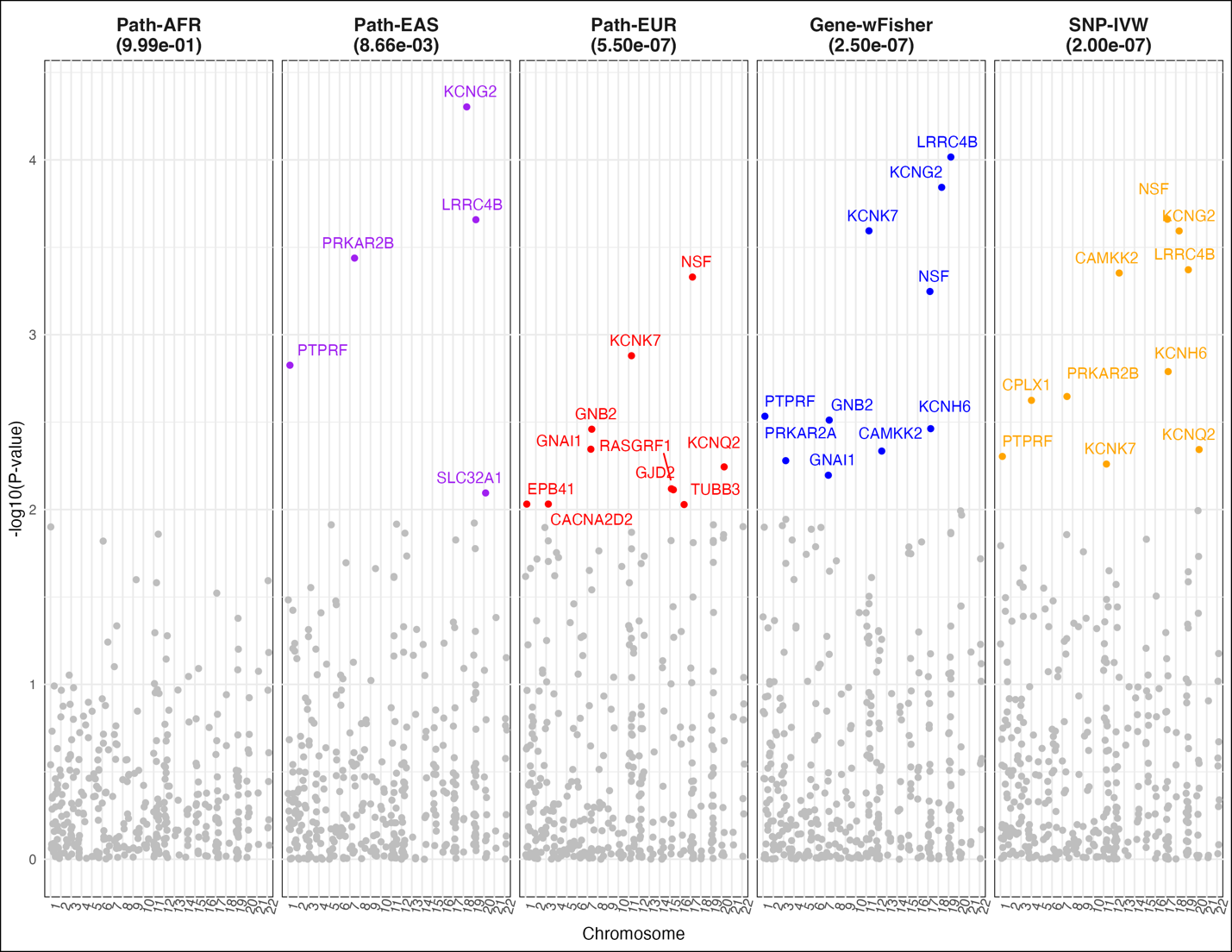
**

**Fig AB. Pathway Analysis Results for the Association Between the REACTOME Nuclear Events Kinase and Transcription Factor Activation Pathway and Schizophrenia.**

**
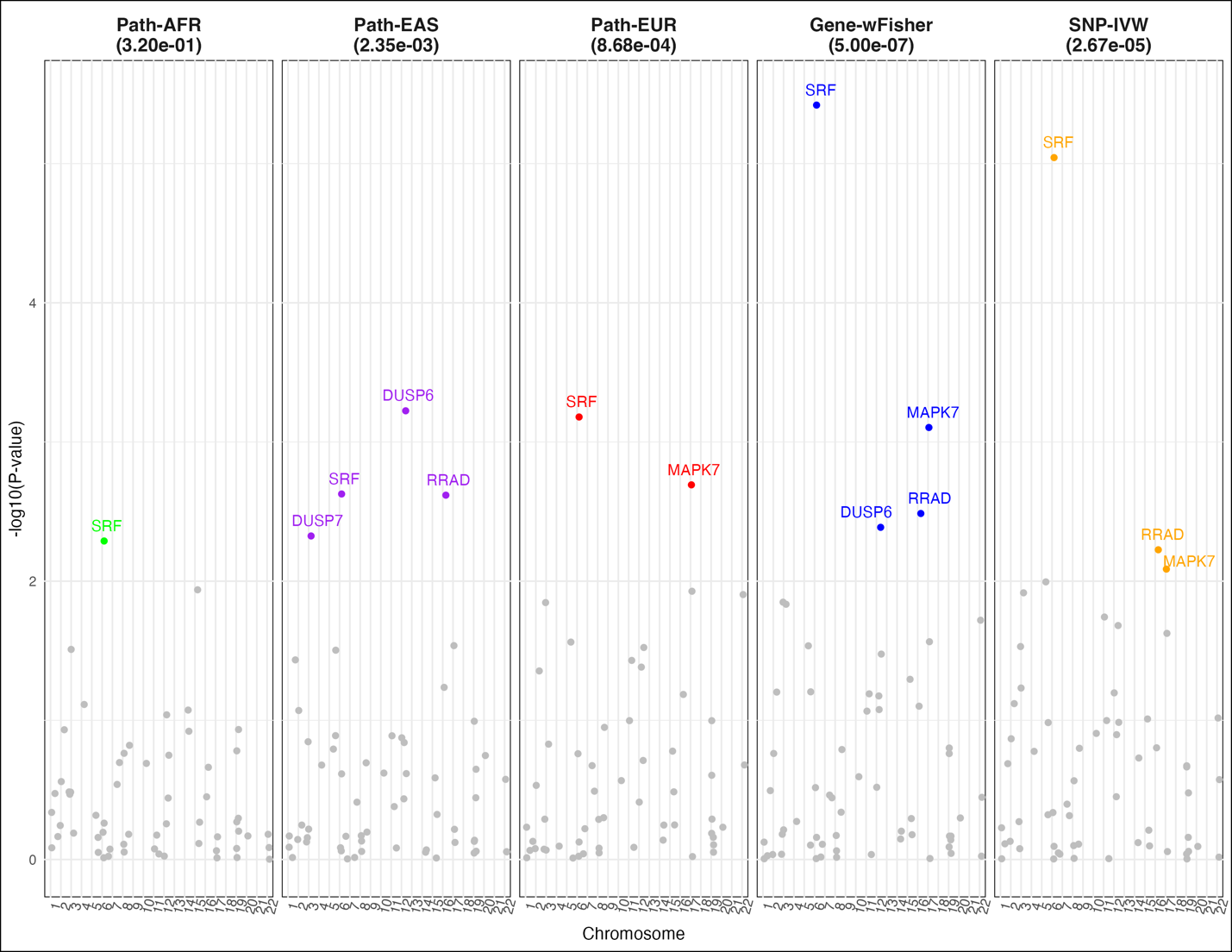
**

**Fig AC. Pathway Analysis Results for the Association Between the REACTOME Regulation of Lipid Metabolism by PPARalpha Pathway and Schizophrenia.**

**
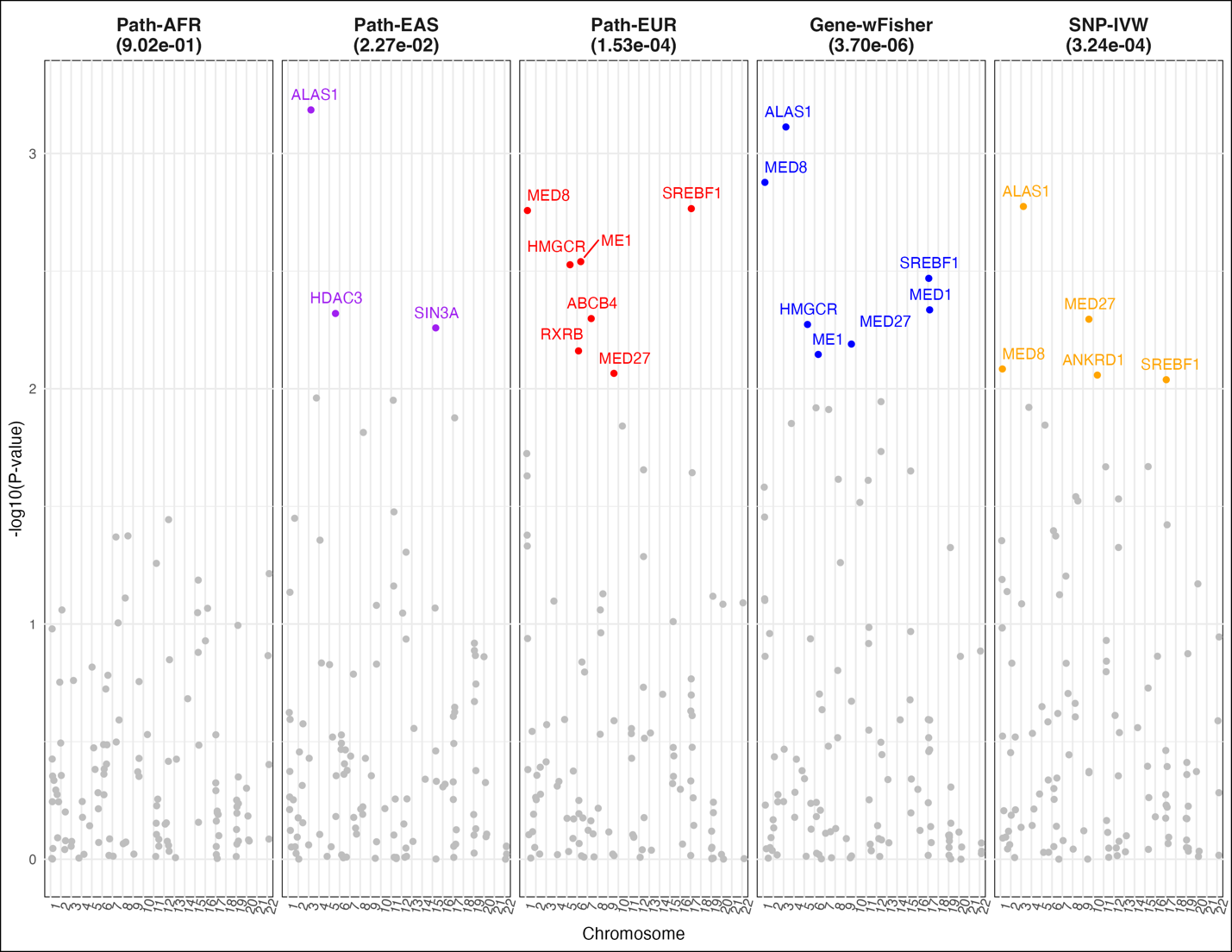
**

**Fig AD. Pathway Analysis Results for the Association Between the REACTOME Resolution of Sister Chromatid Cohesion Pathway and Schizophrenia.**

**
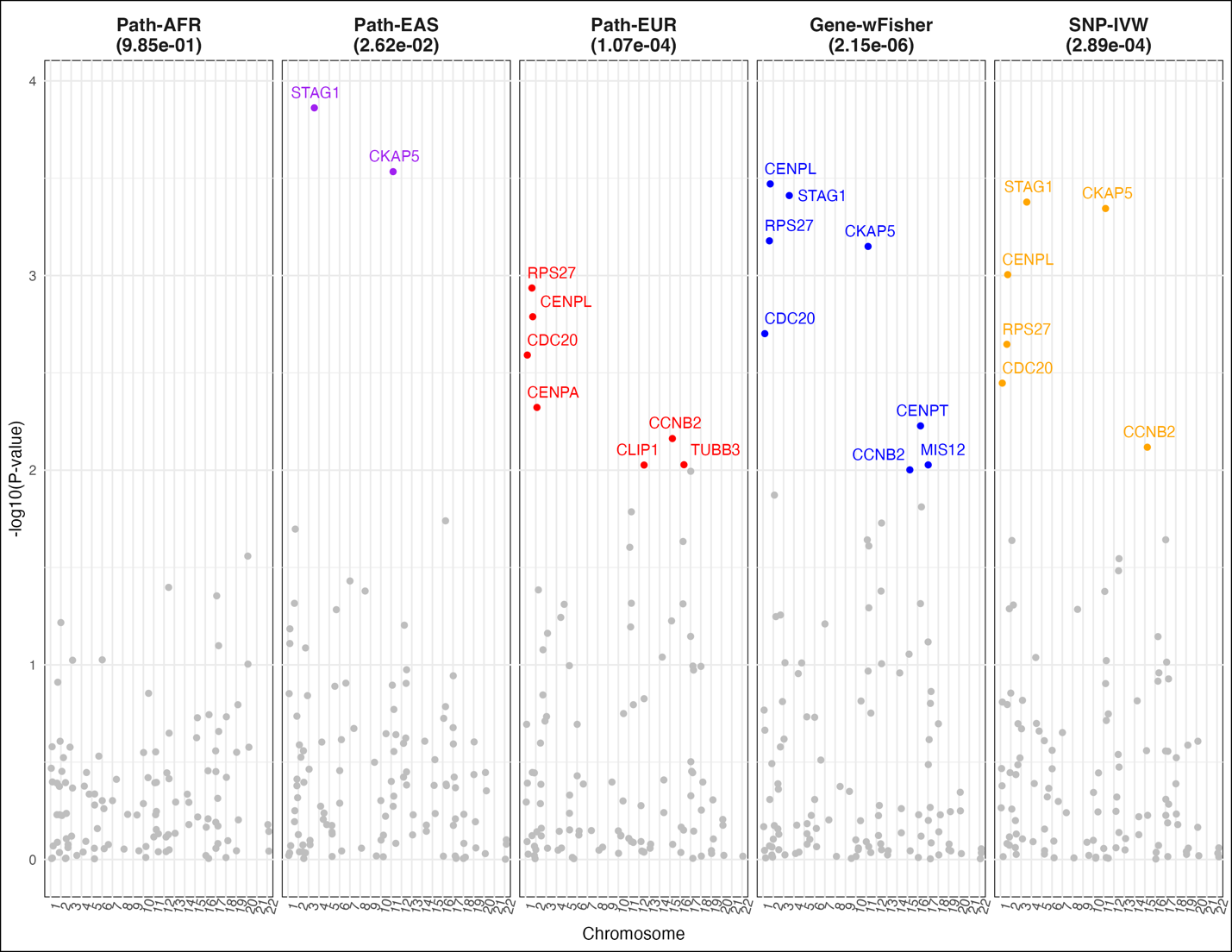
**

**Fig AE. Pathway Analysis Results for the Association Between the REACTOME Rho GTPases Activate Formins Pathway and Schizophrenia.**

**
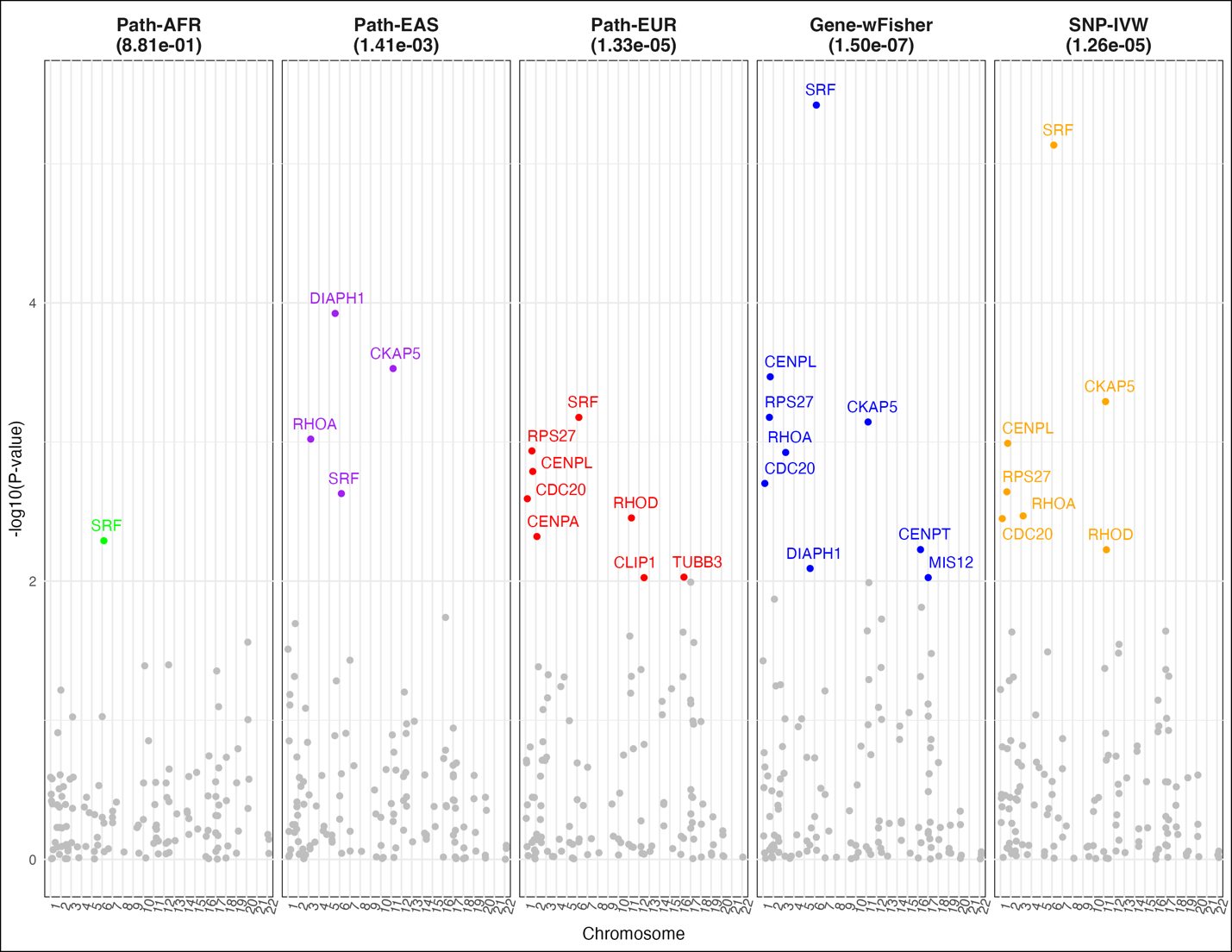
**

**Fig AF. Pathway Analysis Results for the Association Between the REACTOME Rho GTPase Cycle Pathway and Schizophrenia.**

**
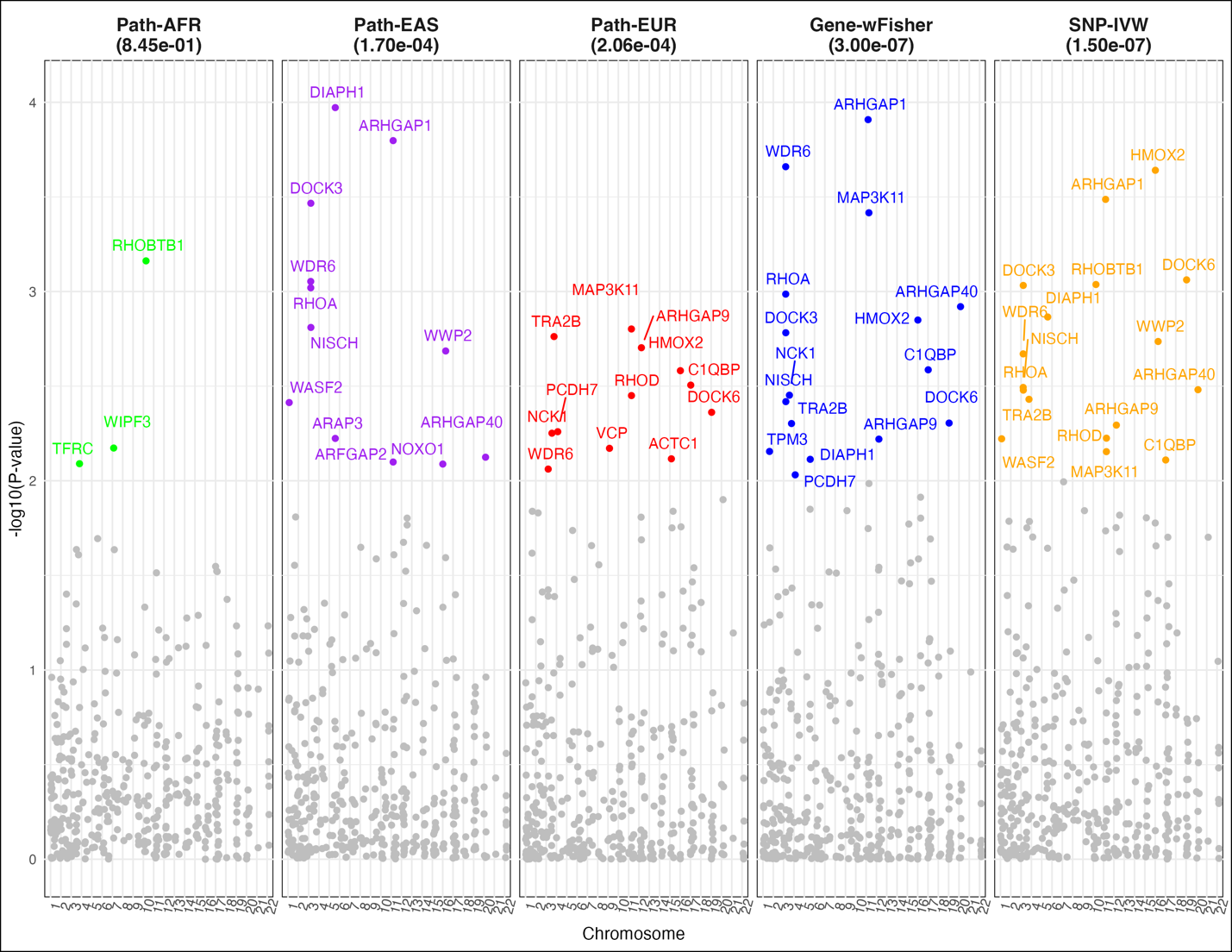
**

**Fig AG. Pathway Analysis Results for the Association Between the REACTOME Rho GTPase Effectors Pathway and Schizophrenia.**

**
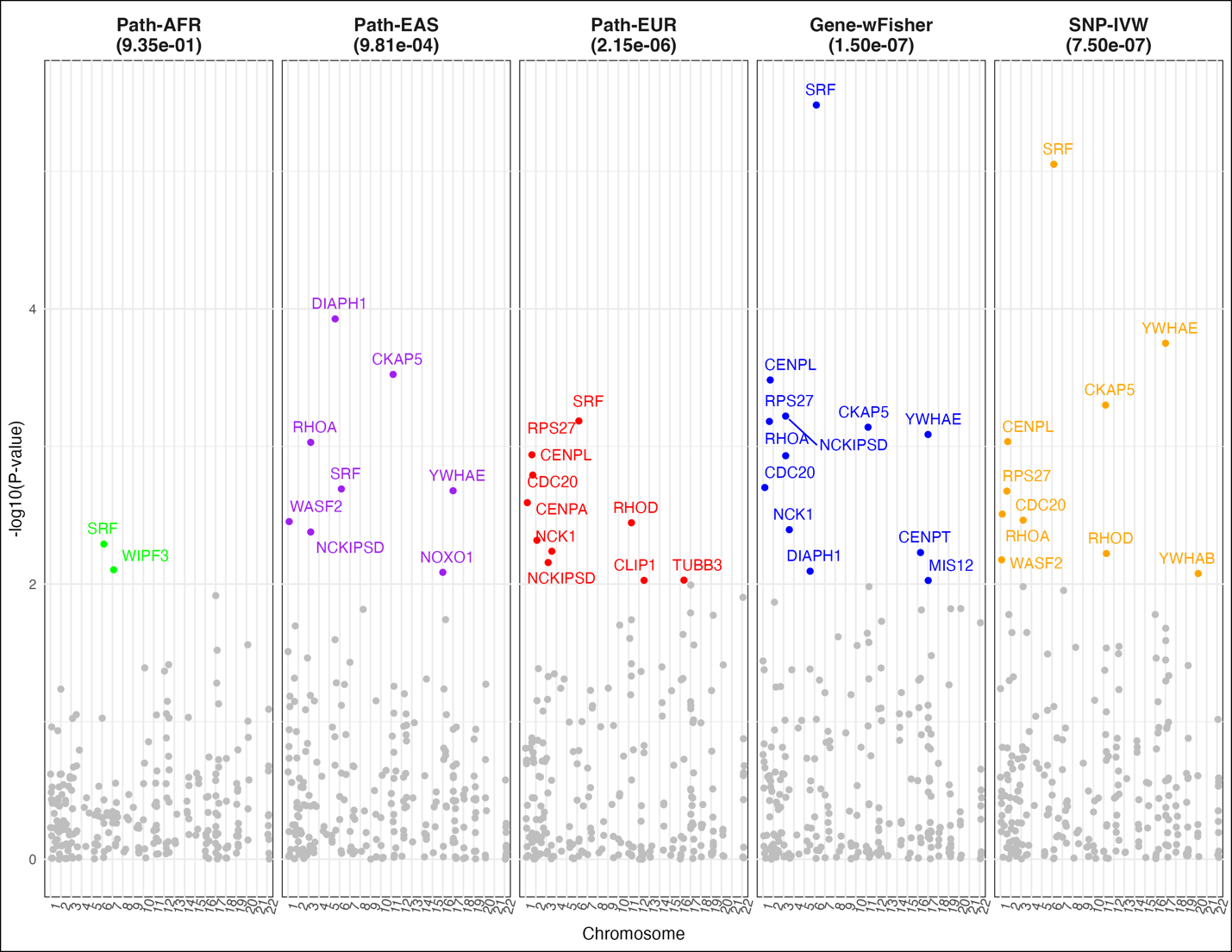
**

**Fig AH. Pathway Analysis Results for the Association Between the REACTOME rRNA Processing Pathway and Schizophrenia.**

**
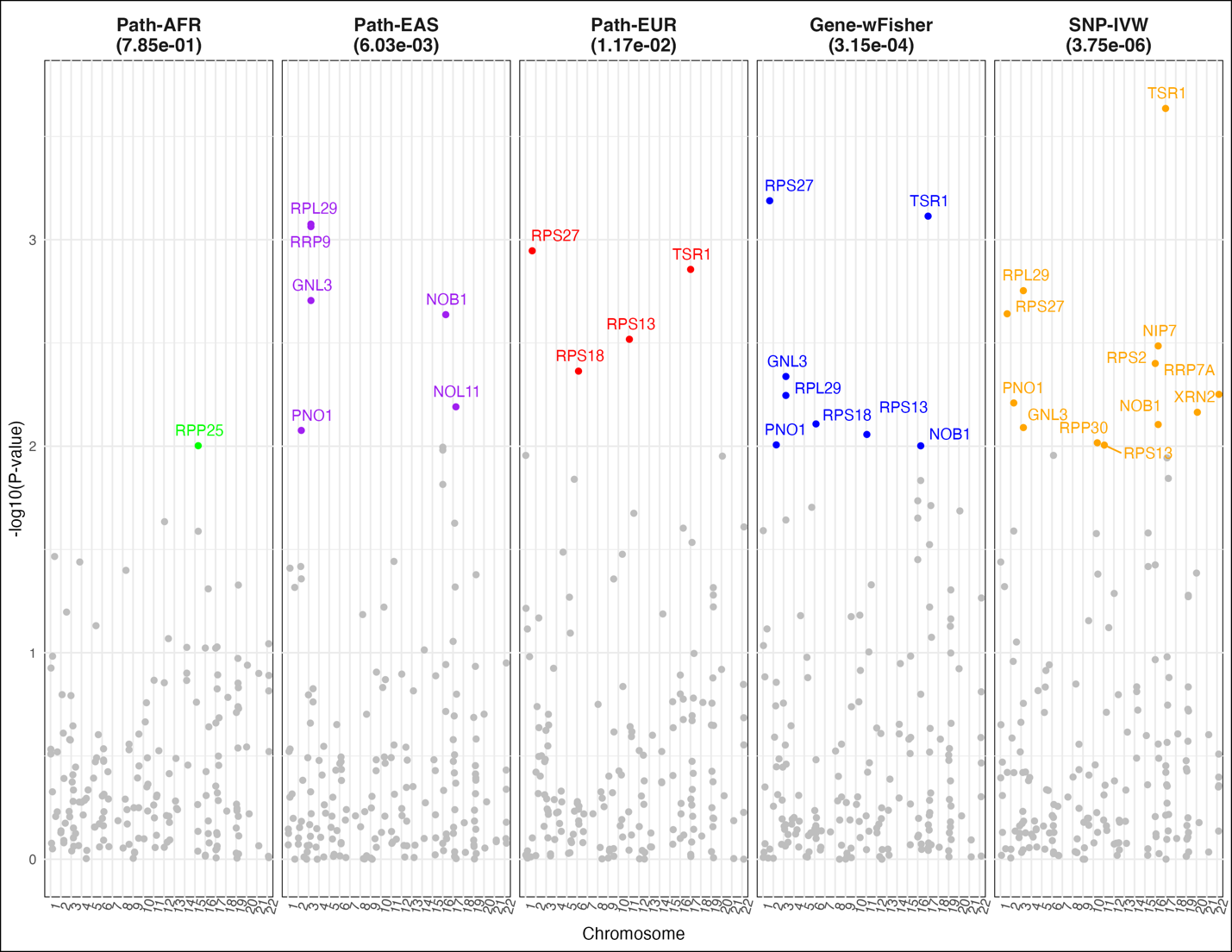
**

**Fig AI. Pathway Analysis Results for the Association Between the REACTOME Signaling by NTRKs Pathway and Schizophrenia.**

**
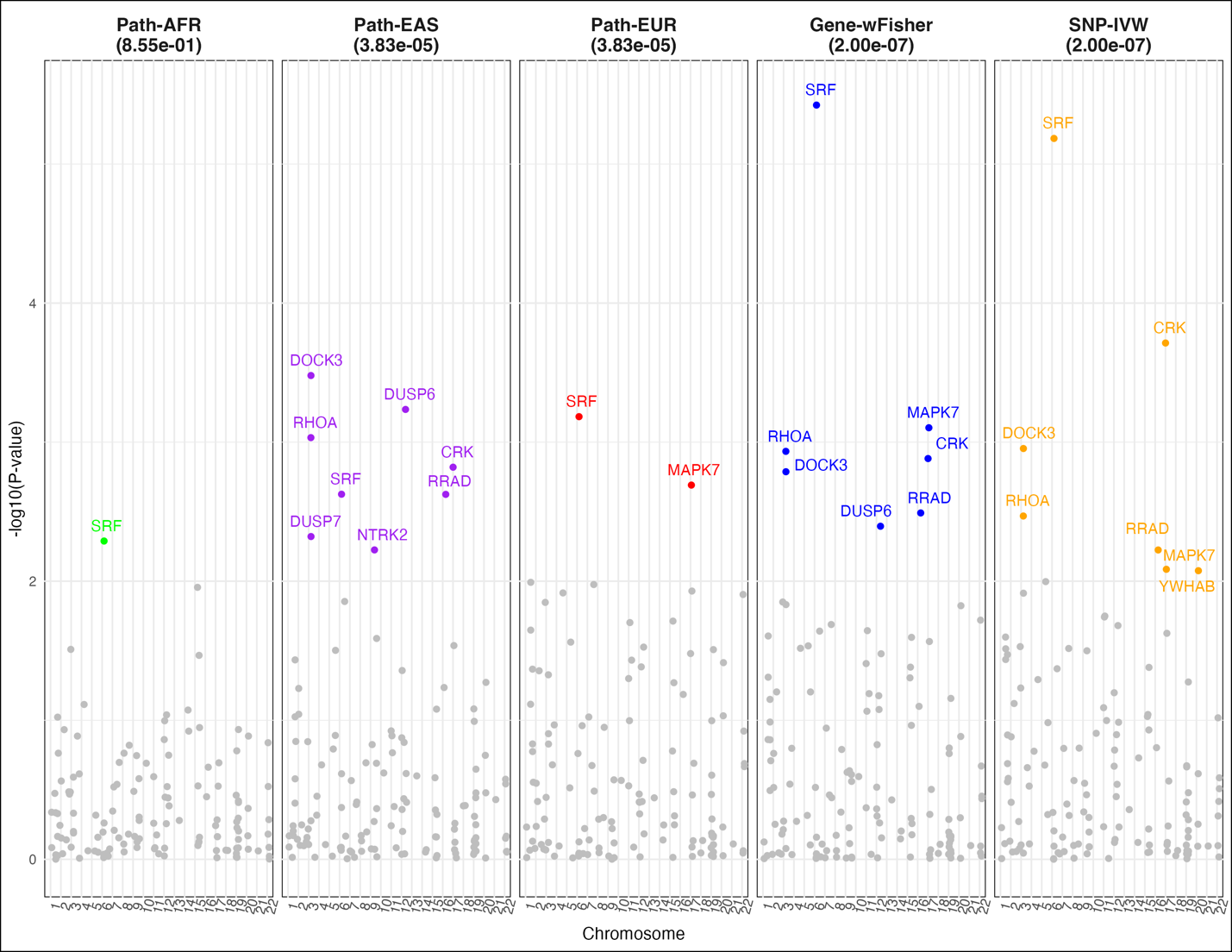
**

**Fig AJ. Pathway Analysis Results for the Association Between the REACTOME Signaling by Nuclear Receptors Pathway and Schizophrenia.**

**
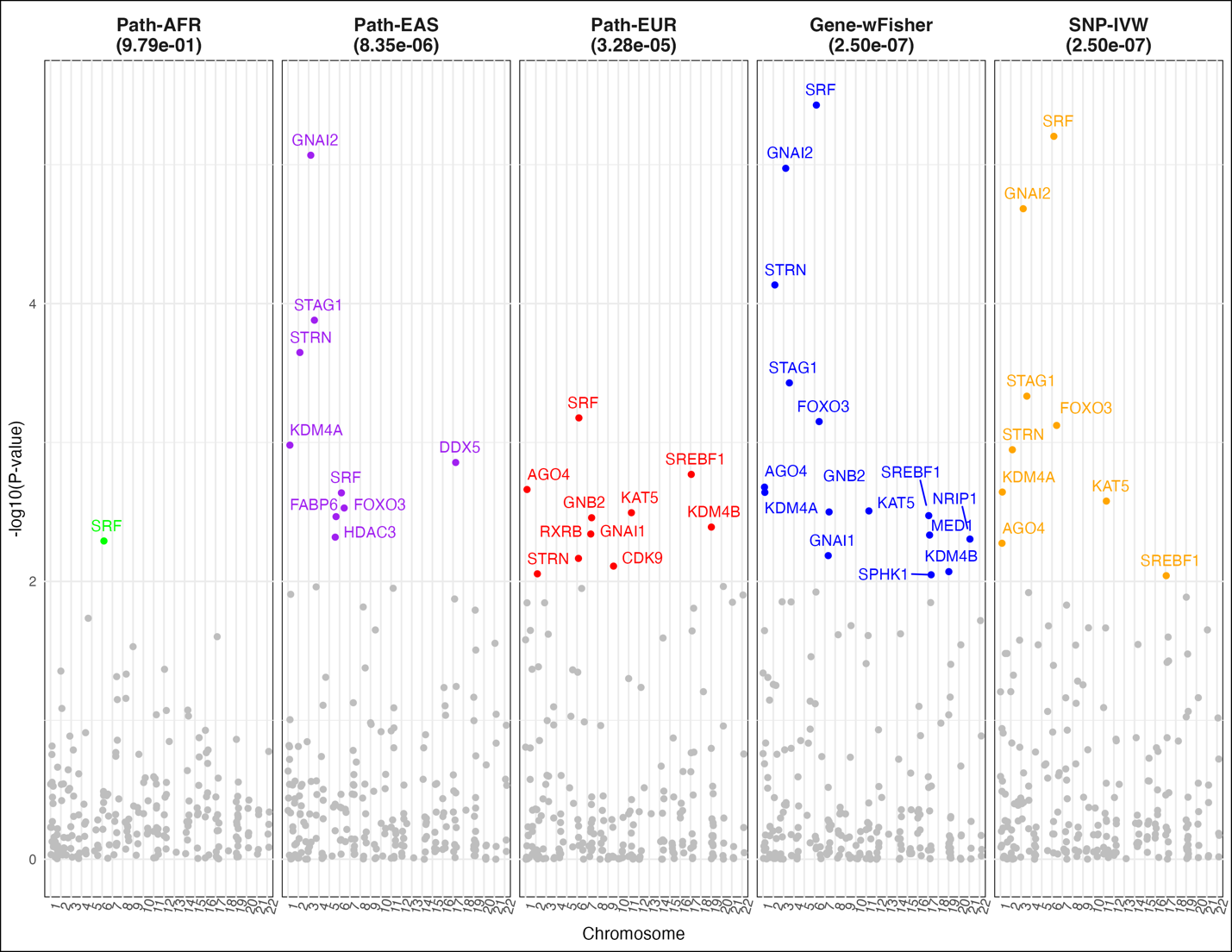
**

**Fig AK. Pathway Analysis Results for the Association Between the REACTOME Signaling by WNT Pathway and Schizophrenia.**

**
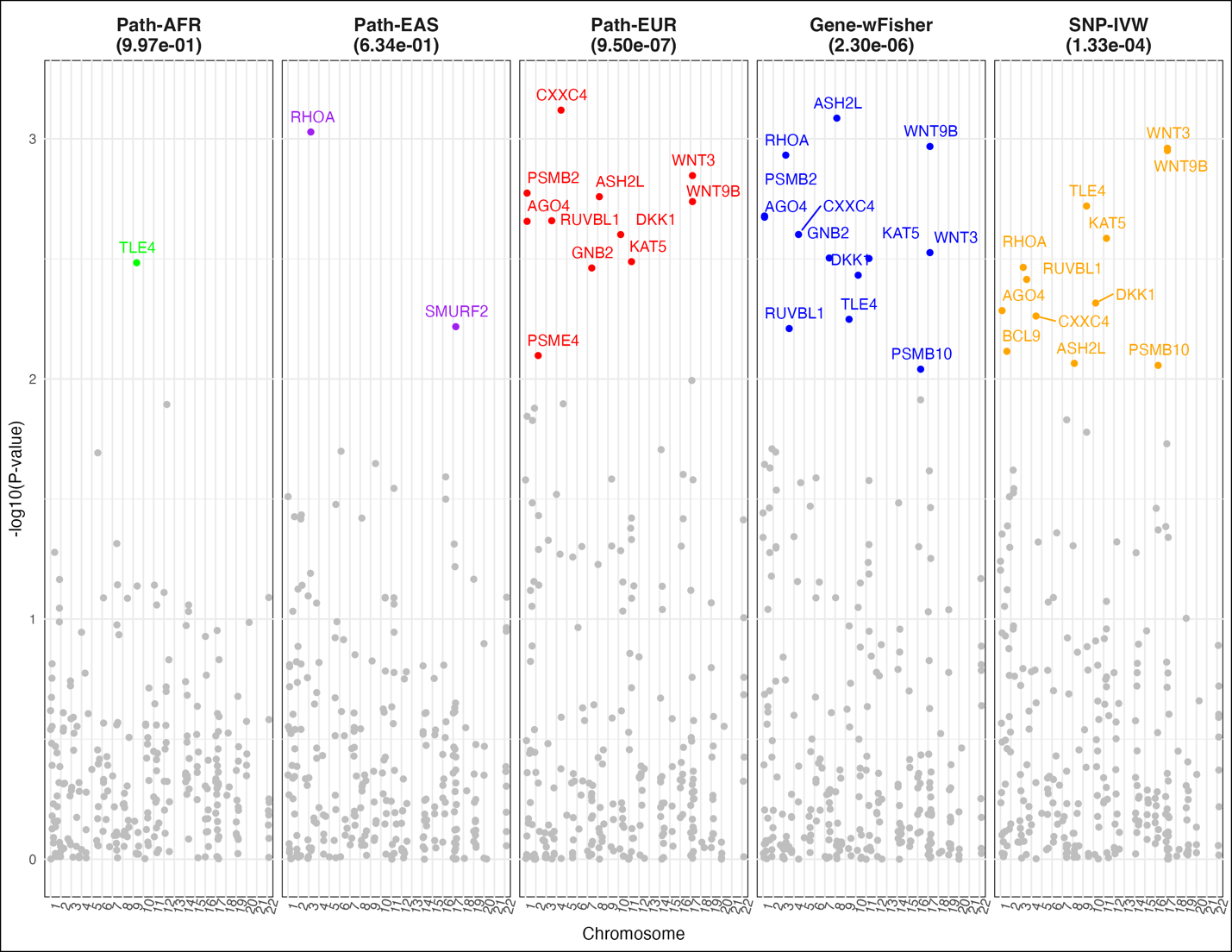
**

**Fig AL. Pathway Analysis Results for the Association Between the REACTOME SLC Mediated Transmembrane Transport Pathway and Schizophrenia.**

**
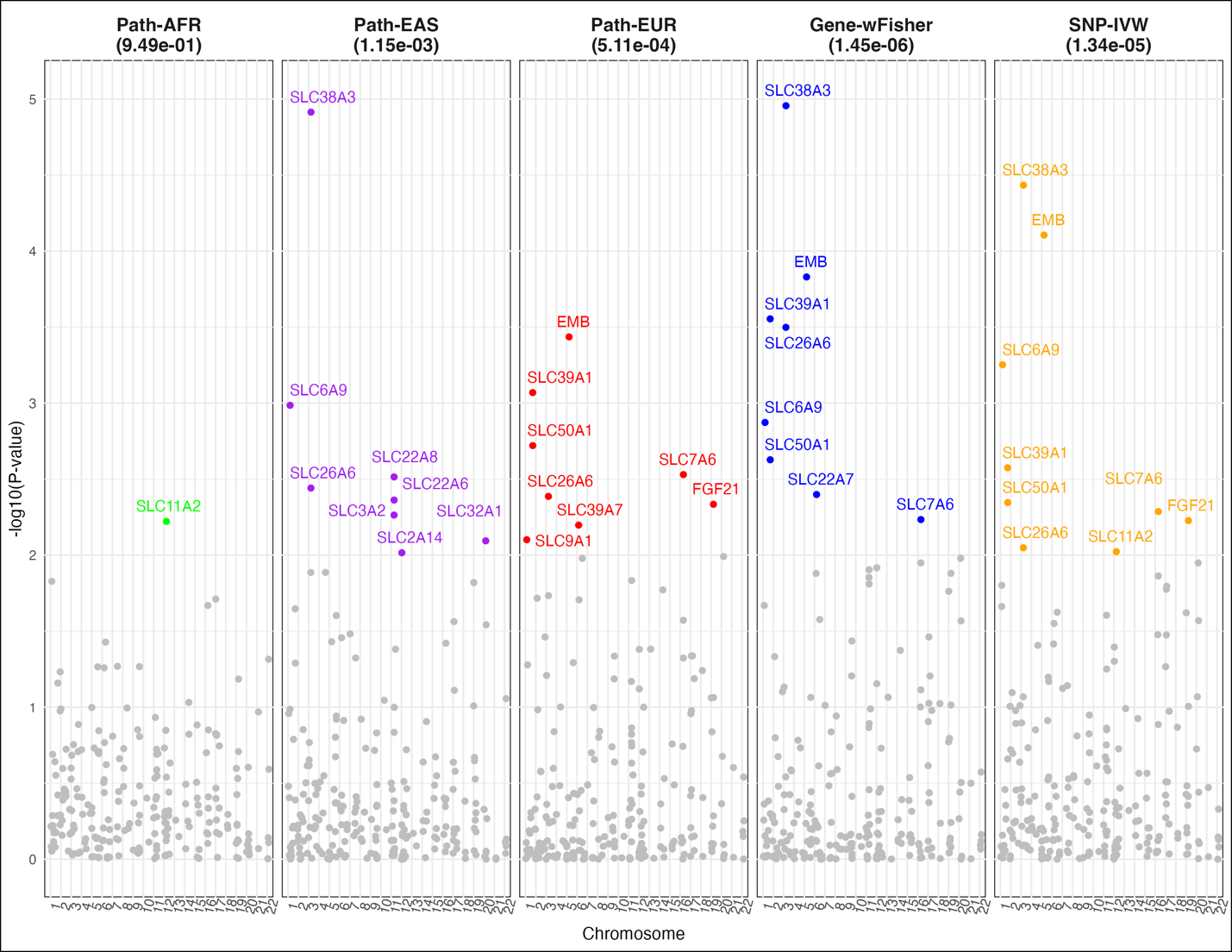
**

**Fig AM. Pathway Analysis Results for the Association Between the REACTOME Transcriptional Regulation by TP53 Pathway and Schizophrenia.**

**
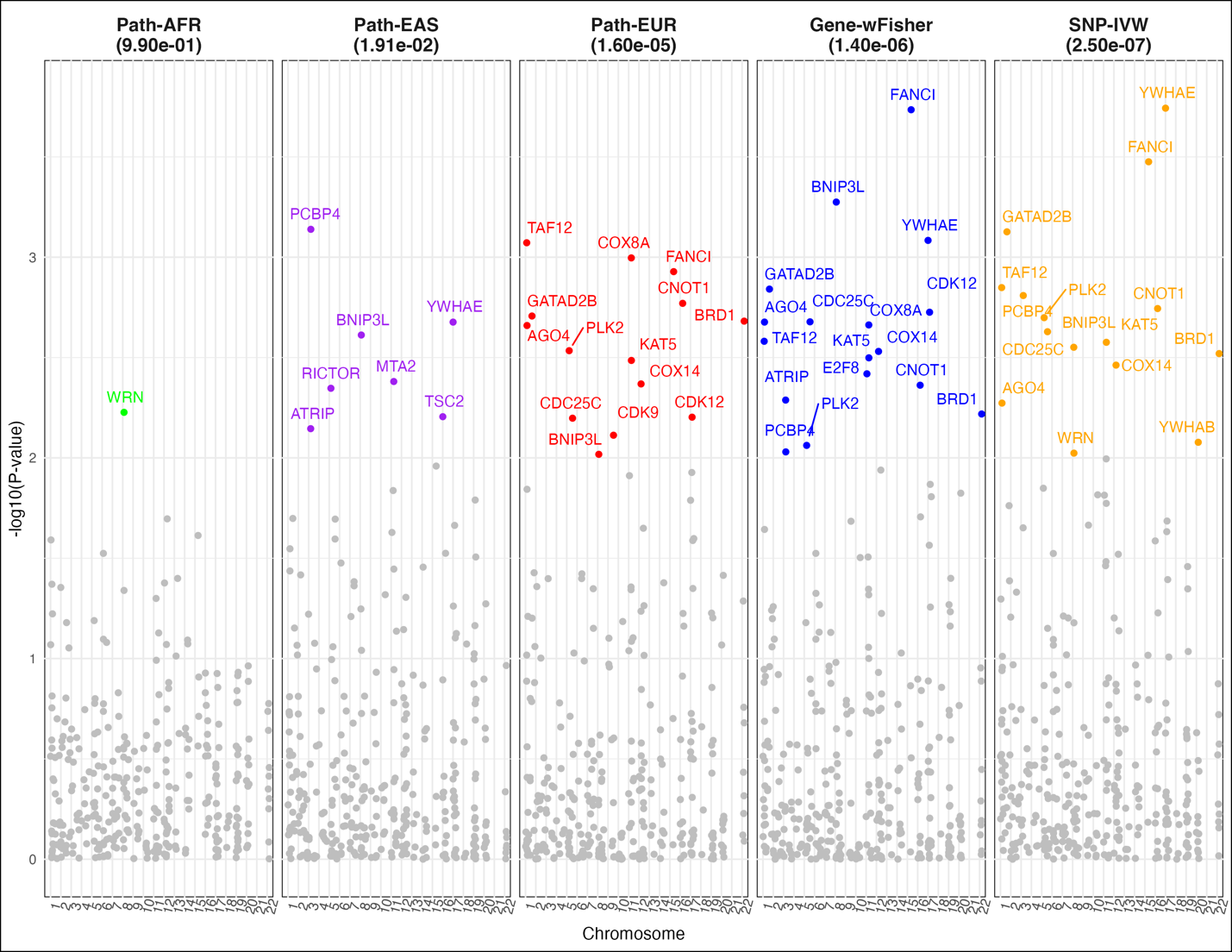
**

**Fig AN. Pathway Analysis Results for the Association Between the REACTOME Translation Pathway and Schizophrenia.**

**
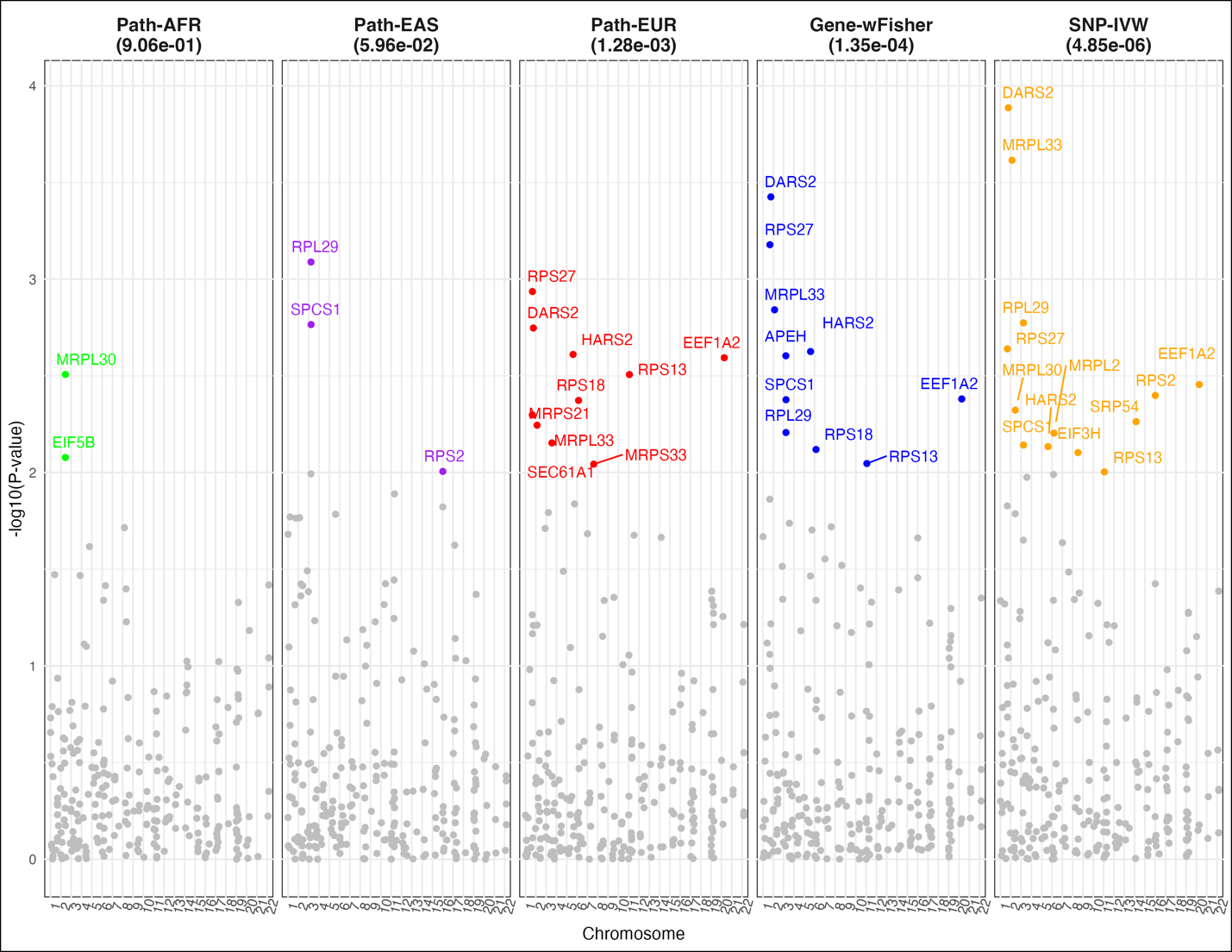
**

**Fig AO. Heatmap of Gene-Level P-Values for Selected Genes Across 23 Significant REACTOME Pathways Associated with Schizophrenia Detected by the SNP-IVW Method.** This heatmap displays gene-level p-values for 91 unique genes, as detailed in Supplementary Table 4, across 23 significant pathways listed in Supplementary Table 3. Each gene, with a p-value below 0.005 as estimated by the Gene-wFisher method, is featured on the x-axis, while pathways are displayed on the y-axis, organized by their respective p-values. Each row in the heatmap corresponds to one significant pathway, with the color intensity of each cell reflecting the gene-level p-value on a -log_10_ scale. Cells for genes not included in a pathway are shaded blue.


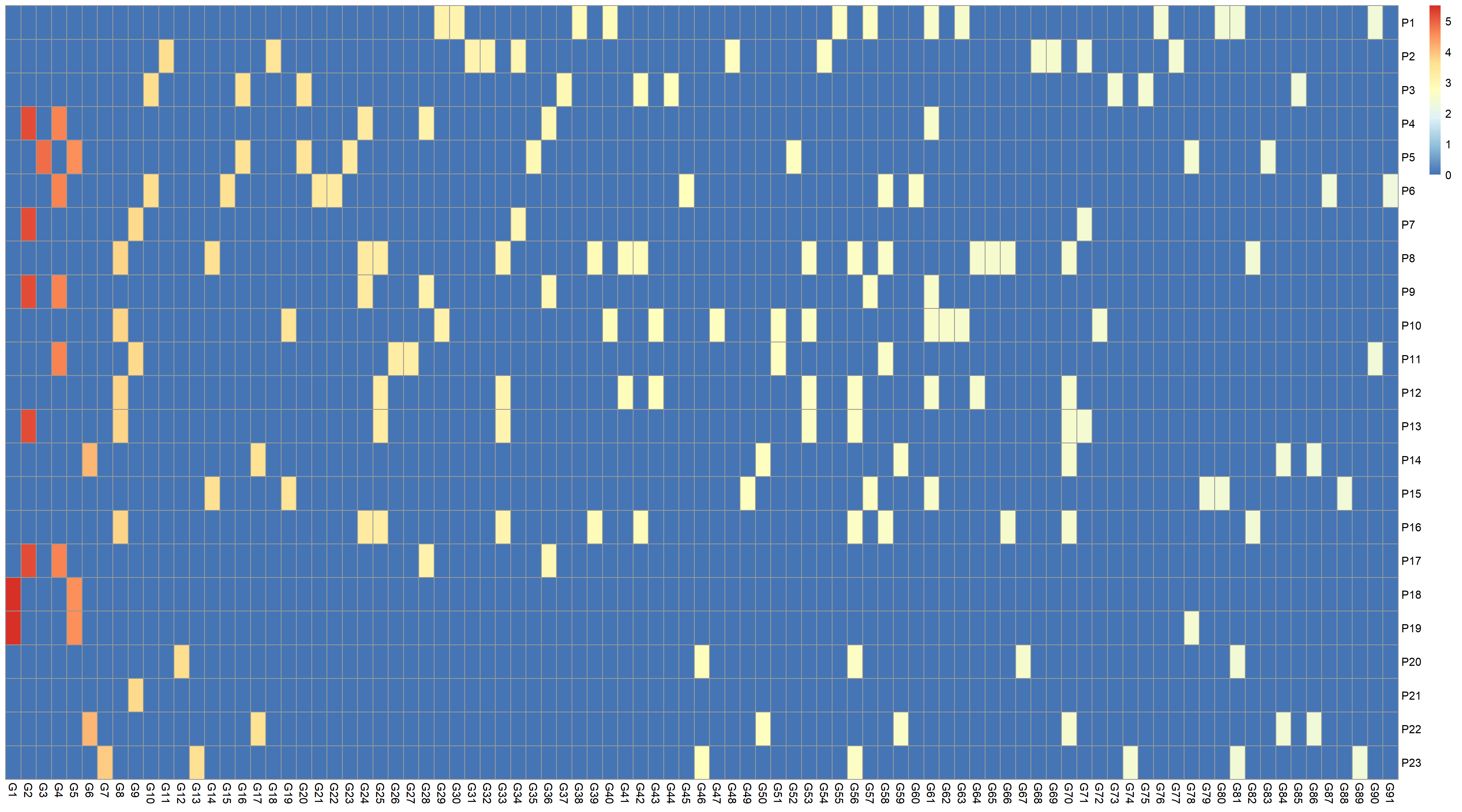

Supplement: S1 Text — (DOCX) [file pgen.1011322.s001.docx]
